# Unravelling Differences in Candidate Genes for Drought Tolerance in Potato (*Solanum tuberosum* L.) by Use of New Functional Microsatellite Markers

**DOI:** 10.3390/genes12040494

**Published:** 2021-03-28

**Authors:** Christina Schumacher, Christoph Tim Krannich, Lisa Maletzki, Karin Köhl, Joachim Kopka, Heike Sprenger, Dirk Karl Hincha, Sylvia Seddig, Rolf Peters, Sadia Hamera, Ellen Zuther, Manuela Haas, Renate Horn

**Affiliations:** 1 Institute of Biological Sciences, Plant Genetics, University of Rostock, Albert-Einstein-Str. 3, 18059 Rostock, Germany; christina.schumacher2017@outlook.com (C.S.); christoph.krannich@gmx.de (C.T.K.); lisa.maletzki@uni-greifswald.de (L.M.); sadia.hamera@uni-rostock.de (S.H.); 2 MPI für Molekulare Pflanzenphysiologie, Am Mühlenberg 1, 14476 Potsdam, Germany; koehl@mpimp-golm.mpg.de (K.K.); kopka@mpimp-golm.mpg.de (J.K.); Heike.Sprenger@bfr.bund.de (H.S.); Zuther@mpimp-golm.mpg.de (E.Z.); manuela.haas@mluk.brandenburg.de (M.H.); 3 Department of Food Safety, German Federal Institute for Risk Assessment, Max-Dohrn Straße 8-10, 10589 Berlin, Germany; 4 Deceased, Formerly MPI für Molekulare Pflanzenphysiologie, Am Mühlenberg 1, 14476 Potsdam, Germany; Hincha@mpimp-golm.mpg.de; 5 Julius Kühn-Institut, Rudolf-Schick-Platz 3, 18190 Sanitz, Germany; sylvia.seddig@julius-kuehn.de; 6 Landwirtschaftskammer Niedersachsen, Dethlingen 14, 29633 Munster, Germany; peters@docpotato.de; 7 PotatoConsult UG, Hiddinger Straße 33, 27374 Visselhövede, Germany; 8 Ministry of Agriculture, Environment and Climate Protection, Henning-Von-Tresckow-Straße 2-13, 14467 Potsdam, Germany

**Keywords:** potato, *Solanum tuberosum*, drought tolerance, microsatellite, candidate gene, aldehyde dehydrogenase, protein phosphatase 2C, 1-aminocyclopropane-1-carboxylate synthase, ethylene responsive transcription factor, poly(ADP-ribose) glycohydrolase

## Abstract

Potato is regarded as drought sensitive and most vulnerable to climate changes. Its cultivation in drought prone regions or under conditions of more frequent drought periods, especially in subtropical areas, requires intensive research to improve drought tolerance in order to guarantee high yields under limited water supplies. A candidate gene approach was used to develop functional simple sequence repeat (SSR) markers for association studies in potato with the aim to enhance breeding for drought tolerance. SSR primer combinations, mostly surrounding interrupted complex and compound repeats, were derived from 103 candidate genes for drought tolerance. Validation of the SSRs was performed in an association panel representing 34 mainly starch potato cultivars. Seventy-five out of 154 SSR primer combinations (49%) resulted in polymorphic, highly reproducible banding patterns with polymorphic information content (PIC) values between 0.11 and 0.90. Five SSR markers identified allelic differences between the potato cultivars that showed significant associations with drought sensitivity. In all cases, the group of drought-sensitive cultivars showed predominantly an additional allele, indicating that selection against these alleles by marker-assisted breeding might confer drought tolerance. Further studies of these differences in the candidate genes will elucidate their role for an improved performance of potatoes under water-limited conditions.

## 1. Introduction

Drought has become the predominant abiotic stress and major yield limiting factor in crops in the past years [1,2,3]. Severe implications for food production have been observed [4,5,6] confronting plant breeding with the challenge of securing food supplies at a global level under these changing growth conditions. As an effect, research into drought tolerance mechanisms has been intensified in most important crops, such as maize [7,8,9], wheat [2,10], and rice [11,12,13]. Drought tolerance involves coordination of heavily interwoven networks of biosynthesis and signaling pathways of phytohormones such as abscisic acid (ABA), ethylene, jasmonate and the production of osmoprotective compounds such as proline, glycine betaine and trehalose [14]. Numerous transcription factors like ABA-responsive element (ABRE) binding proteins, dehydration responsive element binding (DREB) proteins, and ethylene response factors (ERFs) were identified as major players in controlling the expression of genes involved in drought response in plants [15,16,17].

As a temperate-zone crop with a shallow active root zone, potato is sensitive to drought [18,19] and heavy yield losses are predicted based on climate change prognosis [20,21,22]. Morphological and physiological traits such as shoot height, leaf size, number of leaves, photosynthetic rate, biomass as well as tuber yield are affected by water shortage [23]. Damage caused by water deficit is dependent on the developmental stage, the duration and the intensity of the drought period [18,24]. Potato tuber bulking stage (early stress) is the most critical phase with regard to water demands and an inadequate water supply can severely affect tuber yield and tuber quality [18,25]. Evaluation of marketable tuber yield in a panel of 103 European potato cultivars with different maturity identified cultivars with better performance under non-irrigated conditions and pointed at a single nucleotide polymorphism (SNP) PotVar0030768 from the 14 Infinium SNP marker array to be significantly associated with this trait [26]. Potato cultivars with a higher root to shoot ratio due to deeper roots show a better performance under drought conditions [27]. Change of plant morphology towards small, open stem-type canopies combined with shallow but dense root systems may be an interesting approach for improving drought tolerance in potato [28].

Different systems have been used to study drought stress in potato: field trials (irrigated and non-irrigated), rain-out shelters and/or trials under fully controlled conditions [29,30,31] as well as in vitro systems using proxies, such as sorbitol or polyethylene glycol [32,33,34]. Drought stress indices such as the stress susceptibility index (SSI) [35], the stress tolerance index (STI) or the geometric mean productivity (GMP) [36] have been successfully applied to define stress levels in experimental designs. Using an artificial data set, a new drought stress index calculated as deviation of the relative starch yield under drought and control conditions from the experimental median (DRYM) outperformed the other three stress indices in the ability to differentiate between drought sensitive and drought tolerant potato cultivars detached from the yield potential [24].

Transcriptome analyses identified differentially upregulated drought response genes involving ABA and ethylene signaling transduction like protein phosphatase 2C (PP2C), homolog of abscisic acid receptor PYL4 (PYRABACTIN RESISTANCE-LIKE 4), 1-aminocyclopropane-1-carboxylate oxidase (ACO) and ethylene responsive transcription factors in potato [19,37,38,39]. Moreover, expansin and genes involved in osmotic adjustment and protection such as delta-1-pyrroline-5-carboxylate synthase (P5CS), galactinol synthase, inositol-3-phosphate synthase, raffinose synthase (RFS), osmotin-like (OSML) and late embryogenesis abundant proteins (LEA) showed increased expression levels under drought stress. Differentially expressed transcription factors including 10 gene families such as bHLH, bZIP, ERF, HD-ZIP, MYB, NAC and WRKY represented 4.7% of the drought responsive genes in potato [37]. Investigating drought response in stolon tips significant differences in the expression of heat shock 70 kDa protein, aquaporins, bidirectional sugar transporter, peroxidase and pectinesterase/pectinesterase inhibitor as well as lipid-transfer proteins (LTP) were observed [38]. Among genes that showed the highest induction or repression comparing drought stress response with re-watering and control treatment were those coding for TAS14, WRKY, ERF and bHLHL transcription factors, auxin response protein, and abscisic acid 8′-hydroxylases [39]. In addition, protein kinases, mostly receptor-like kinases, but also kinases like mitogen-activated protein kinase kinase (MAPKK), histidine kinases, cyclin-dependent kinases and Sucrose non-fermenting 1-related protein kinase 2 (SnRK2) were differentially expressed under drought stress in potato [39,40]. Comparing Gwaizda/Oberon and Taijun/Owacja as two pairs of drought tolerant and drought sensitive potato cultivars 22 novel drought response genes were identified [41]. For six genes (armadillo/β-catenin-like repeat-containing protein, carbohydrate transporter, MAPKKK 15, nitrate transporter 2.7, nonspecific LTP and serine carboxypeptidase-like 19 protein), corresponding Arabidopsis mutants showed altered gene expressions and improved drought tolerance [41]. Constitutive differences in gene expression under both control and drought stress conditions have also been observed between drought tolerant and sensitive potato cultivars [40]. These included among others S-adenosyl-L-methionine-dependent methyltransferase superfamily, cytochrome P450, UDP-glycosyl transferase family proteins, disease-related genes and receptor kinases [40]. A random forest model allowed prediction of drought tolerance for different potato cultivars based on eight metabolite (e.g., 2-oxo-glutaric acid, ribonic acid, galactonic acid) and 19 transcript markers (e.g., glucosyltransferase, O-methyltransferase, extensin, poly(ADP-ribose) glycohydrolase, lipoxygenase) with an accuracy of 82.6% [31].

Transcriptome studies in potato identified drought response genes by differential gene expression, but have so far not distinguished between possible different alleles of the individual genes. Potato cultivars are highly heterozygous tetraploids allowing the presence of up to four different alleles per gene. A rapid and easy conversion of identified drought response candidate genes into molecular markers detecting variation in these genes will be essential for successful drought tolerance breeding. Simple sequence repeats (SSRs), also known as microsatellites, are the most frequently used markers for genotyping potato landraces [42]. Apart from pure forms, SSRs can also occur in complex, compound, interrupted pure, interrupted compound and interrupted complex forms [43,44]. In potato, SSR markers based on pure, compound and complex repeats have been successfully developed [45,46,47,48,49,50]. A new potato genetic identity kit for fingerprinting consisting of 51 SSR markers was finally assembled out of previously published SSR markers and new ones [51]. In addition, a genetic reference map based on SSR markers was developed [51]. Another set of 277 SSR markers based on pure repeats was derived from the whole potato genome sequence [43]. SSRs have been successfully applied in genetic diversity studies in potato cultivars, landraces and germplasms [51,52,53,54], in population structure analyses of association panels [55,56] and for the development of linkage maps for QTL analyses [47,57,58].

In this study, we present the identification of new functional SSR markers derived from potential candidate genes for drought tolerance in potato as well as association studies performed with these SSRs in a panel of 34 mainly starch potato cultivars, which were ranked for drought tolerance using DRYM [24] as drought tolerance index. The approach allowed the development of 75 new SSR markers that represent a valuable tool for mapping and further genetic studies in potato. Five of the SSR markers detected allelic variations in the potato cultivars that were significantly associated with drought sensitivity. Future functional studies of these differences in the candidate genes will reveal the role in drought tolerance. Combined application of three of these SSR markers allowed the identification of drought-sensitive cultivars in potato.

## 2. Methods

### 2.1. Extraction of Genomic DNA from Potato

Fully developed leaves of 34 tetraploid potato cultivars (Supplementary Table S1) were harvested from a polytunnel trial at the Max Planck Institute of Molecular Plant Physiology in Potsdam. This association panel consisting of 34 mainly starch potato cultivars was provided by German breeding companies (Böhm-Nordkartoffel-Agrarproduktion, Strehlow; Norika Nordring-Kartoffelzucht- u. Vermehrungs-GmbH, Groß-Lüsewitz; SaKa Pflanzenzucht, Hamburg). Genomic DNA was isolated by the protocol of Doyle and Doyle [59]. 

### 2.2. Drought Tolerance Assessment

Drought tolerance of 34 European potato cultivars was assessed based on performance data from drought tolerance trials on three managed field sites in Germany (Max Planck Institute of Molecular Plant Physiology Potsdam-Golm [F1, F3] and Potato Research Station Dethlingen [F2, F5] in 2011 and 2012; Julius Kühn-Institute Groß-Lüsewitz [F4], only in 2012) as described in Sprenger et al. [24], but the field trials of 2013 were omitted. All five investigated field trials represent early stress and corresponding watered controls, whereas from the three omitted field trials in 2013 two field trials represented late stress scenarios. Plants were grown from seed tubers supplied by the respective breeding companies (Supplementary Table S1, [24]) and cultivated under agricultural conditions with additional irrigation for the optimally watered control plants and without or reduced irrigation for the drought stress treated plants (Supplementary Table S2). All treatments received additional water from precipitation, with the exception of the drought treatment in experiment F4, which was grown under a rain-out shelter. Plant performance was assessed based on the tuber starch yield. Tubers were harvested and weighted at the end of the experiment and tuber starch content was measured gravimetrically. Starch yield is the product of tuber mass and starch content for each replicate.

Data evaluation was performed in SAS (SAS 9.3 Level 1MO, Cary, NC, USA). The effect of cultivar and treatment on starch yield was assessed by analysis of variance with procedure GLM. Based on the results, data from the treatment “30% Field Capacity (FC)” at the field site Dethlingen 2011 were excluded from the analysis as they were not significantly different from the control treatment “50% FC”. In 2012, data from treatment “30% FC” and “no irrigation” were taken together as stress treatment. Drought tolerance was calculated as deviation of the relative starch yield of a genotype from the median relative starch yield of the respective experiment (*DRYM*) (see Equation (1)). Relative starch yield (*RelSY_GxEi_*) is the ratio of the starch yield under drought stress divided by the average starch yield of the respective cultivar (*Gx*) under control conditions in the same experiment (*Ei*).

(1) $$DRYM_{G_{x},E_{i}}=RelSY_{G_{x},E_{i}}-median\left(RelSY_{E_{i}}\right)$$

This calculation centers *DRYM* around zero, with drought tolerant cultivars showing positive values and sensitive genotypes negatives values.

Analysis of covariance (ANCOVA) was performed with procedure GLM, testing the effect of experiment and cultivar (random factors) and normalized starch yield as covariable on *DRYM*. Means comparison for cultivars was performed with Ryan–Einot–Gabriel–Welch-Test (REGWQ) that provides multiple testing corrections.

### 2.3. Mining for Simple Sequence Repeats (SSR) and Characterization

Genomic sequences of the candidate genes for drought tolerance were retrieved from different databases as Phytozome (https://phytozome.jgi.doe.gov/pz/portal.html (accessed on 27 March 2021)), EnsemblPlants ([60], http://plants.ensembl.org/index.html (accessed on 27 March 2021)), Spud DB Potato Genomics Resource ([43], http://potatogenomics.plantbiology.msu.edu (accessed on 27 March 2021)), Sol Genomics Network (https://solgenomics.net/ (accessed on 27 March 2021)) and NCBI (https://www.ncbi.nlm.nih.gov/ (accessed on 27 March 2021)). The retrieved sequences were then mined for repeats using the default parameters in the program Microsatellite repeats finder (http://insilico.ehu.es/mini_tools/microsatellites/ (accessed on 27 March 2021)). In case, no microsatellites could be detected in the gene sequence (exons and introns), sequences of the 5′- and 3′-UTR were studied. If still no repeats could be observed the flanking downstream and upstream sequences were investigated for microsatellites, which were then termed intergenic SSRs. The microsatellites are given as present in the coding strand as this is the custom for naming repeats in coding regions [44]. Primer combinations were then derived in sequence segments of about 240 bp around the repeat sequence using the program Primer3 (http://bioinfo.ut.ee/primer3-0.4.0/ (accessed on 27 March 2021)). All 154 primer combinations are given in Supplementary Table S3. The polymerase chain reaction (PCR) was performed as described in Sajer et al. [61]. However, the DNA concentration was increased from 10 ng/µL to 20 ng/µL considering the heterozygous tetraploid genetic constitution of cultivated potatoes. The PCR reaction volume was reduced to 15 µL. By using IRD-labelled M13 primers (IRD700 and IRD800) (Biomers, Ulm, Germany) PCR products were fluorescence labelled for the detection on the DNA Analyzer Model 4300 (LI-COR Biosciences, Lincoln, NE, USA). The newly derived SSR primer combinations were analyzed in the association panel of 34 potato cultivars. The estimation of the polymorphic information content (PIC) values and of the expected heterozygosity (H_exp_) was performed according to Bali et al. [52].

In order to determine the statistical significance of the banding patterns concerning drought tolerance the Fisher’s exact test was calculated online in a case-control type study dividing the association panel into two groups of 17 drought tolerant and 17 drought sensitive cultivars (https://www.langsrud.com/stat/Fishertest.htm (accessed on 27 March 2021)). A significant association was reached at a (two-sided) *p* value (calculated probability) < 0.05.

### 2.4. AFLP Analyses

The potato DNA was digested with *EcoR*I and *Mse*I and ligated to *EcoR*I and *Mse*I adapters as described in Vos et al. [62]. AFLP analyses were performed based on a pre-amplification with E01 (5′-GACTGCGTACCAATTCA-3′) and M02 (5′-GATGAGTCCTGAGTAAC-3′) as primers. For the selective amplification, the *EcoR*I primers E32–E35 were combined with *Mse*I primers M47–M49 or M60–M62 (Supplementary Table S4). In addition, E32M50 was used. All primer sequences and numbers were used according to http://wheat.pw.usda.gov/ggpages/keygeneAFLPs.html (accessed on 27 March 2021) (Keygene, N.V., Wageningen, NL, USA). IRD-labelled *Eco*RI primers (IRD700 and IRD800) from Biomers (Ulm, Germany) were used for fluorescence labeling of the selective amplification products, which were separated on a DNA Analyzer Model 4300 (LI-COR Biosciences, Lincoln, NE, USA).

### 2.5. Population Structure

In total, 54 linkage group specific SSR primer combinations (Supplementary Table S5) were used to determine the population structure of the association panel, resulting in at least four SSR primer combinations per linkage group. In addition, data from 19 AFLP primer combinations were included to have better genome coverage (Supplementary Table S4). The program NTSYSpc 2.2 (Numerical Taxonomy System for personal computer) was used to identify and reveal structures in multivariate data [63]. The similarity matrix was based on the Jaccard’s coefficient to reflect the genetic similarity of the 34 different potato cultivars. For clustering, UPGMA (Unweighted Pair Group Method with Arithmetic Mean) was applied to obtain a dendrogram. Mantel test was conducted, and a cophenetic correlation coefficient computed, which allowed a statement regarding the reliability of the UPGMA-dendrogram in representing the similarity matrix.

## 3. Results

### 3.1. Characterization of Functional SSR Markers Derived from Candidate Genes for Drought Tolerance in Potato

**Figure 1:**
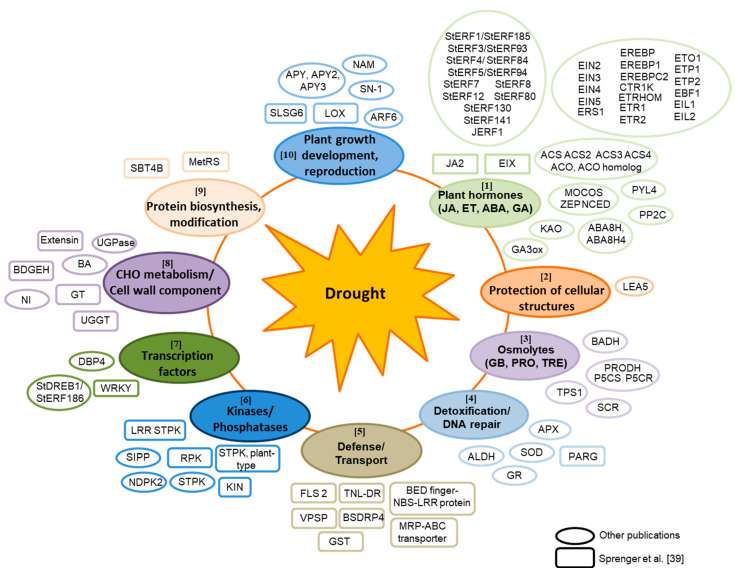
Overview about processes involved in response to water deficiency showing the selected candidate genes for drought tolerance used in this study for the development of SSR markers. Abbreviations: ABA: Abscisic acid, ABA8H: ABA 8′-hydroxylase, ACO: 1-Aminocyclopropane-1-carboxylic acid oxidase, ACS: 1-Aminocyclopropane-1-carboxylic acid synthase, ALDH: Aldehyde dehydrogenase, APX: Ascorbate peroxidase, APY: Apyrase, ARF: Auxin response factor, BA: β-amylase, BDGEH: β-D-glucan exohydrolase, BADH: Betaine aldehyde dehydrogenase, BSDRP4: Bacterial spot disease resistance protein 4, BEAF: Boundary element associated factor, BED: BEAF and DREF, CHO: Carbohydrate, CTR1K: Constitutive Triple Response1 kinase, DBP: DNA binding protein, P5CS: Delta 1-pyrroline-5-carboxylate synthetase, DREB: Dehydration-Responsive Element-Binding, DREF: DNA replication-related element factor, EBF1: EIN3-binding F-box protein 1, EIN: Ethylene insensitive, EIL: Ethylene-Insensitive3-Like, EREBP: Ethylene-responsive element-binding protein, EREBPC2: Ethylene responsive element binding protein C2, ERF: Ethylene response factor, ERS 1: Ethylene response sensor 1, ET: Ethylene, ETO1: Ethylene-overproduction protein 1, ETP: EIN2 targeting protein, ETR1: Ethylene receptor 1, ETRHOM: Ethylene receptor homolog, EIX: Ethylene-inducing xylanase, FLS 2: Flagellin-sensing 2, GA3ox: Gibberellin 3-oxidase, GA: Gibberellin, GB: Glycine betaine, GR: Glutathione reductase, GST: Glutathione-S-transferase, GT: Glucosyltransferase, JA: Jasmonic acid, JA2: Jasmonic acid 2, JERF1: Jasmonate and Ethylene Response Factor 1, KAO: Ent-kaurenoic acid oxidase, KIN: Kinase, LEA5: Late embryogenesis abundant 5, LOX: Lipoxygenase, LRR: Leucine-rich repeat, MetRS: Methionyl-tRNA synthetase, MOCOS: Molybdenum Cofactor sulfurase, MRP-ABC: Multidrug resistance protein ATP binding cassette, NAM: No apical meristem, NBS: Nucleotide binding site, NI: Neutral invertase, NCED: 9-Cis-epoxycarotenoid dioxygenase, NDPK2: Nucleoside diphosphate protein kinase 2, P5CR: Pyrroline-5-carboxylate reductase, P5CS: Pyrroline-5-carboxylate synthase, PARG: Poly (ADP-ribose) glycohydrolase, PP2C: Protein phosphatase 2C, PRO: Proline, PRODH: Proline dehydrogenase, PYL4: Pyrabactin resistance-like 4, RPK: Receptor protein kinase, SBT4B: subtilase 4B, SCR: SCARECROW, SIPP: Soluble inorganic pyrophosphatase, SLSG6: S-locus specific glycoprotein S6, SN-1: Snaking-1, SOD: Superoxide dismutase, St: Solanum tuberosum, STPK: Serine/threonine protein kinase, TIR: Toll/interleukin-1 receptor-like protein, TNL-DR: TIR-NBS-LRR disease resistance, TPS1: Trehalose-6-phosphate synthase 1, TRE: Trehalose, UGGT: UDP-glucose glycosyltransferase, UGPase: UDP-glucose-1-phosphate uridylyltransferase, VPSP: Vacuolar protein sorting protein, WRKY: WRKY transcription factor, ZEP: Zeaxanthin epoxidase

In total, 154 SSR primer combinations were derived from 103 candidate genes for drought tolerance (Figure 1; Supplementary Table S3). Eighty of the candidate genes were taken from previous publications (reviewed in Krannich et al. [14]) and 23 were identified in a drought-related transcriptome study as top transcript markers for drought tolerance prediction (Supplementary Table S6, [39]). Some candidate genes are involved in biosynthesis and signaling pathways of plant hormones like ethylene and ABA as well as the biosynthesis of osmolytes and starch (Figure 1).

Other potential candidate genes for drought tolerance were transcription factors, kinases/phosphatases and transporters. Another group covered the area of plant development, especially tuber formation. In addition, genes encoding proteins involved in cell wall formation, defense, protective cellular structures, protein modification and DNA repair as well as detoxification were included.

**Figure 2:**
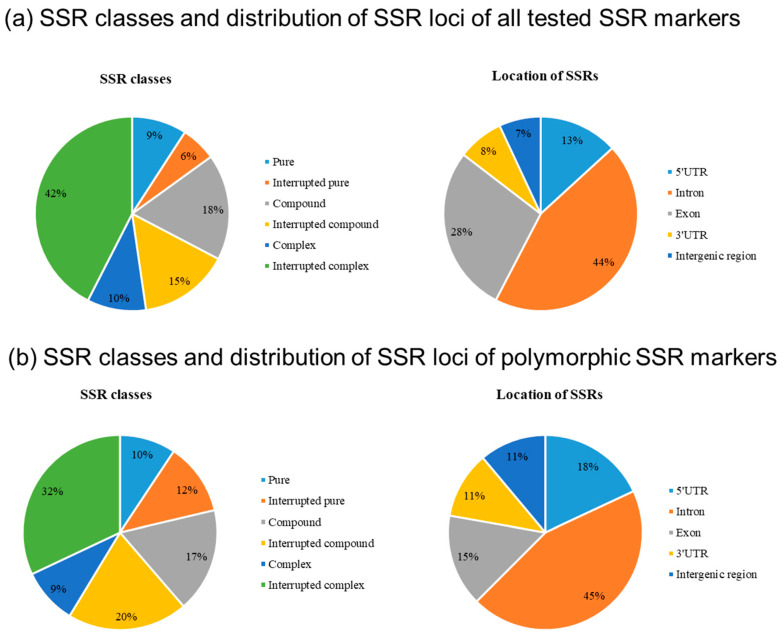
Composition and distribution of microsatellites in 5′-UTR-regions, exons, introns and 3′-UTR-regions of the investigated 103 candidate genes. (**a**) SSR classes and distribution of SSR loci of all tested SSR markers; (**b**) SSR classes and distribution of SSR loci of polymorphic SSR markers in the potato association panel (34 tetraploid potato cultivars).

Most of the detected microsatellites showed an interrupted complex structure of the type (repeats)_k_ X (repeats)_n_(repeats)_m_ (42%) or in 18% a compound structure such as (repeats)_n_ (repeats)_m_, where the repeats were di-, tri-, tetra-, penta- and hexanucleotide motifs, the number of repeats n + m or k + n+m added up to at least six and the number of nucleotides X between the repeats did not exceed four, with nine exceptions (Figure 2A, Supplementary Table S3). Most of the microsatellite repeats were located in intron regions (44%), followed by 28% in exon regions. A lower number of SSRs was present in 5′-UTRs (13%), in 3′-UTRs (8%), and in intergenic regions (7%) (Figure 2a). From 154 microsatellites tested and optimized in an association panel of 34 tetraploid starch potato cultivars, 75 SSR primer combinations (49%) gave reproducible polymorphic banding patterns (Supplementary Table S7). For these 75 SSR markers, a total of 278 alleles were observed resulting in an average of 3.7 alleles per SSR marker. The minority of these polymorphic SSR markers represented complex repeats (9%) and pure repeats (10%), followed by interrupted pure repeats (12%) (Figure 2b). Compound (17%), interrupted compound (20%) and interrupted complex (32%) repeats were the most prominent SSR types.

Only seven candidate genes for drought tolerance (PGSC0003DMG400021476, PGSC0003DMG400000284, PGSC0003DMG400021426, PGSC0003DMG400009719, PGSC0003DMG400021651, PGSC0003DMG402026767, PGSC0003DMG400014417) contained pure repeats. These repeats were (AT)_n_, (GA)_n_, (ACT)_n_, (TTA)_n_ and (CGG)_n_. In three of the candidate genes, 1-aminocyclopropane-1-carboxylate synthase 4 (PGSC0003DMG400021651, ([ACT]_n_], delta-1-pyrroline-5-carboxylate synthetase (PGSC0003DMG402026767; [TTA]_n_) and ethylene response factor ERF12 (PGSC0003DMG400014417; [CGG]_n_), SSRs had been previously described [43], but had not been screened with regard to drought tolerance. The polymorphic information content (PIC) for the 75 SSR markers ranged between 0.11 and 0.90 and for the expected heterozygosity (H_e_) between 0.11 and 0.91 (Supplementary Table S7). We also tested exceptions from X > 4 in nine cases by allowing X = 5 (HRO_BDGEH_1A, HRO_BDGEH_1B, HRO_BFNLRP_2C, HRO_GLUCT_7B, HRO_STPKPT_1A) or X = 6 (HRO_ETOP1_3, HRO_ETP2_2, HRO_ETR2_1, HRO_GLUCT_7), because there were no other possibilities for exploring microsatellites in these genes. In four cases, this resulted also in polymorphic banding patterns and might be considered as a possible option. Polymorphic SSR markers were mostly derived from intron sequences (45%), but also to a considerable percentage from 5′- and 3′-untranslated regions (29%), where changes in the repeat number might have an influence on gene expression. Only a low percentage of polymorphic SSR markers (15%) were obtained from exon regions as in case of PP2C (PGSC0003DMG400011321) and ethylene responsive transcription factor (PGSC0003DMG400002185). Here a change in the number of repeats will lead to changes in the encoded proteins.

**Figure 3:**
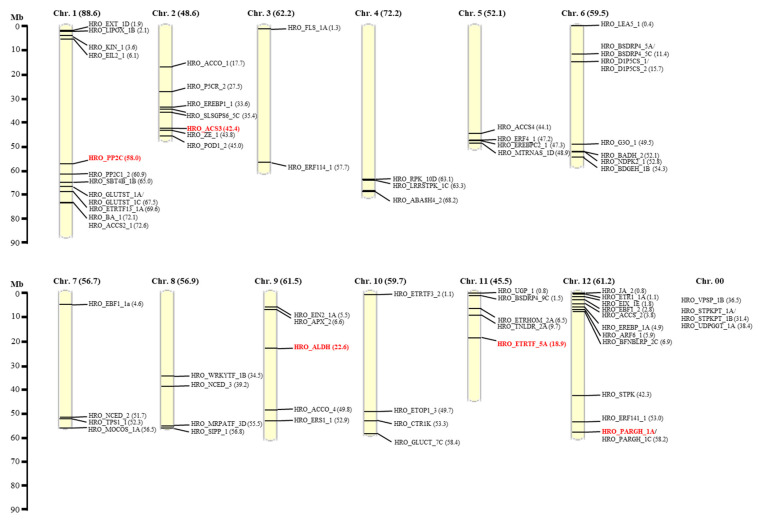
Physical location of the candidate gene specific SSR markers according to the potato reference genome (The Potato Genome Consortium 2011).

A single locus was amplified by nearly all developed SSR primer combinations, resulting in 1-4 bands for each potato cultivar. The exception was the primer combination for aldehyde dehydrogenase (ALDH), which detected at least two loci. All 12 chromosomes carried candidate genes for drought tolerance that allowed development of functional SSR markers. A clustering of candidate genes was observed in the distal parts of linkage group (LG)1, LG2, LG5 and LG6 as well as the proximal parts of LG1 and LG12 (Figure 3).

### 3.2. Ranking of the 34 Starch Potato Cultivars According to Drought Tolerance

**Figure 4:**
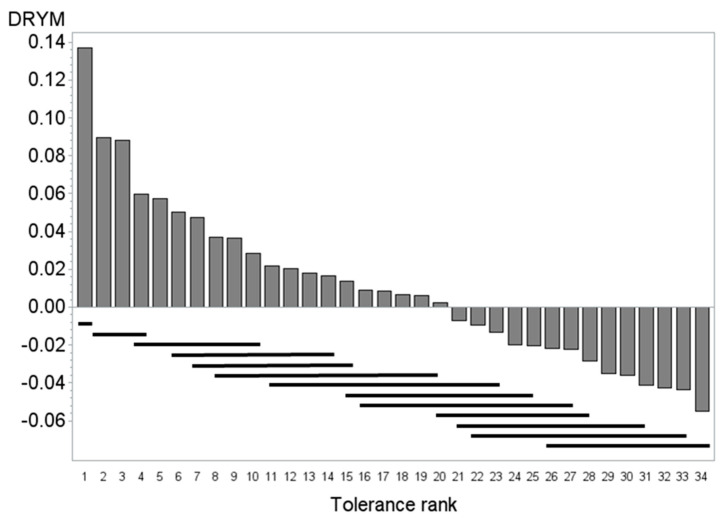
Ranking of the 34 potato varieties of the association panel according to the drought tolerance. Drought tolerance index (DRYM) of 34 potato varieties against rank of cultivar according to drought tolerance (DRYM) (1 most drought tolerant, 34 most sensitive). The bars at the bottom of the figure indicate the grouping according to the means comparison (REGWQ, α = 0.1). Cultivars that are underlined by the same bar are not significantly different in DRYM, e.g., 1 (most tolerant group) is different from 2 to 4 that belong into the second most tolerant group.

Drought tolerance of 34 potato cultivars (Supplementary Table S1) was assessed from the tuber starch yield obtained under optimal and reduced water supply in five field experiments. These experiments were performed for two years (2011 and 2012) on three sites (details see [24]). Analysis of covariance indicated that starch yield was significantly affected by the drought treatment in all experiments [24]. The drought tolerance index DRYM was calculated as the deviation of the relative starch yield from the experimental median of the starch yield (Figure 4). Drought tolerant potato cultivars show positive DRYM values, whereas drought sensitive genotypes have negative values. Analysis of covariance indicated a significant effect of cultivar on DRYM (*p* < 0.0001), suggesting a significant genetic variance of drought tolerance within the population. The means test (see bars at the bottom of Figure 4) indicates a more or less continuous variation in tolerance. Tolerance was significantly higher (α = 0.1) in the cultivars with the ranks 1 to 14 compared to those of rank 20 to 34. Subsequent comparison of means (REGWQ-Test, α = 0.1) yielded the ranking shown in Supplementary Table S1.

### 3.3. Population Structure of the Investigated 34 Potato Cultivars

The 34 potato cultivars were analyzed with 19 AFLP primer combinations (Supplementary Table S4) and 54 linkage group-specific SSR primers (Supplementary Table S5). The population structure shown in an UPGMA dendrogram was based on the Jaccard’s coefficient (Supplementary Figure S1). The Mantel test resulted in a cophenetic correlation coefficient of r = 0.62, indicating a moderate fit. No clustering of the potato cultivars was observed that would lead to a false association of markers regarding drought tolerance.

### 3.4. Identification of SSR Markers Associated with Drought Tolerance

**Figure 5:**
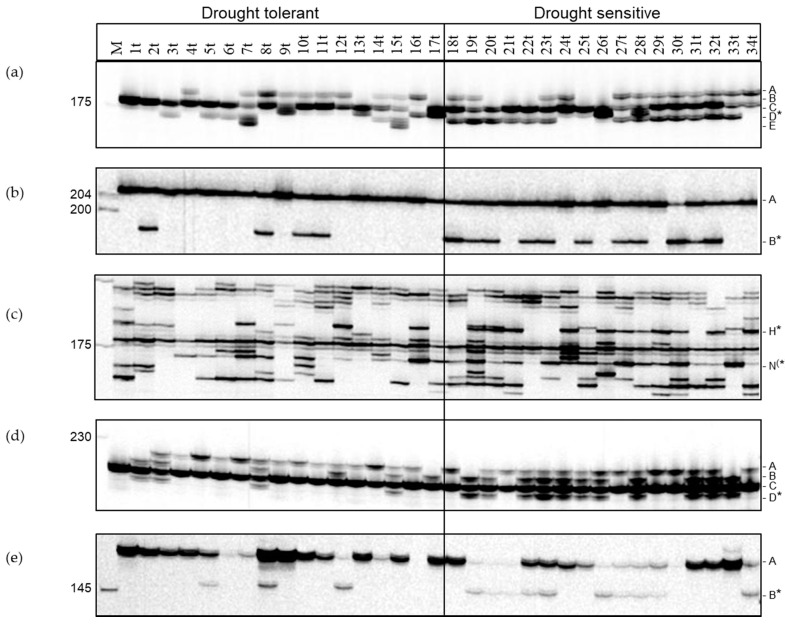
SSR markers associated with drought sensitivity in an association panel of 34 mostly starch potato varieties ranked according to drought tolerance (1t most drought tolerant, 34t most sensitive). (**a**) SSR primer combination HRO_ACS3; (**b**) SSR primer combination HRO-PP2C_1; (**c**) SSR primer combination HRO_ALDH (only relevant bands are marked); (**d**) SSR primer combination HRO_ETRTF_5a; (**e**) SSR primer combination HRO_PARG_1A; M: 50–350 IRD-Marker; Marker bands are alphabetically labelled starting with the largest fragment. Bands associated with drought sensitivity are marked with asterisks.

Association studies applying a case-control type approach were performed within the association panel of 34 potato cultivars, which had been previously ranked according to their drought tolerance using DRYM as drought tolerance index (1t = most tolerant and 34t = most sensitive) (Figure 4, Supplementary Table S1). After splitting the panel into two groups, one of drought tolerant (1t–17t) and one of drought-sensitive (18t–34t) cultivars, all bands were tested for association within these two groups using the Fisher’s Exact test (Supplementary Table S8). This association study allowed the identification of differences in five SSR markers significantly associated with drought sensitivity (Figure 5).

The first primer combination HRO_ACS3 was derived from the 1-aminocyclopropane-1-carboxylate synthase 3 (ACS3, PGSC0003DMG400021426, located on LG2) representing a rate-limiting step of the ethylene biosynthesis. This primer combination amplified 1-4 alleles in the different cultivars (Figure 5a). The band of 173 bp (HRO_ACS3_D) was significantly associated with drought sensitivity (*p* = 0.0366). This band was present in 13 drought sensitive cultivars. In addition, most of the more drought tolerant cultivars showed only one to two bands, whereas the more sensitive cultivars tended to display two to three alleles (Table 1).

The second primer combination HRO_PP2C amplified two alleles, a monomorphic band of 205 bp and a smaller band of 196 bp that was predominantly present in the drought sensitive group (HRO_PP2C_1_B, *p* = 0.0366) (Figure 5b). This SSR detected differences in the gene encoding a protein phosphatase 2C (PP2C, PGSC0003DMG400011321, located on LG1) as part of the ABA signaling pathway.

The third primer combination HRO_ALDH was derived from the aldehyde dehydrogenase (ALDH, PGSC0003DMG400034597, located on LG9). This primer combination amplified more than one locus showing on average 6.4 bands per cultivar. One band of 184 bp (HRO_ALDH_H, *p* = 0.0366) was significantly associated with drought sensitivity, a second one of 172 bp (HRO_ALDH_N, *p* = 0.0854) was only weakly associated (Figure 5c), but proved to be important for further analyses.

With the fourth primer combination HRO_ETRTF_5a derived from an ethylene responsive transcription factor (ERF, PGSC0003DMG400002185, located on LG 11) two to four alleles were amplified (Figure 5d). Again, the more drought sensitive cultivars showed a more complex pattern. The band HRO_ETRTF_5a_D (217 bp) was associated with drought sensitivity (*p* = 0.0366). The band was present in 11 drought sensitive cultivars, but only in four tolerant cultivars.

The last SSR marker (HRO_PARG_1A) was derived from a gene encoding a poly (ADP-ribose) glycohydrolase (PARG, PGSC0003DMG400029361, located on LG 12), which is involved in DNA repair. The primers amplified one to two alleles per cultivar with the lower band of 147 bp showing association with drought sensitivity (*p* = 0.0324) (Figure 5e).

The banding pattern of all five candidate genes was characterized by an additional allele in the group of drought sensitive cultivars, which was significantly associated with drought sensitivity indicating that in all cases these bands might represent alleles inferring less drought tolerance (Table 2).

### 3.5. Selection Using Functional SSR Markers Associated with Drought Tolerance

**Figure 6:**
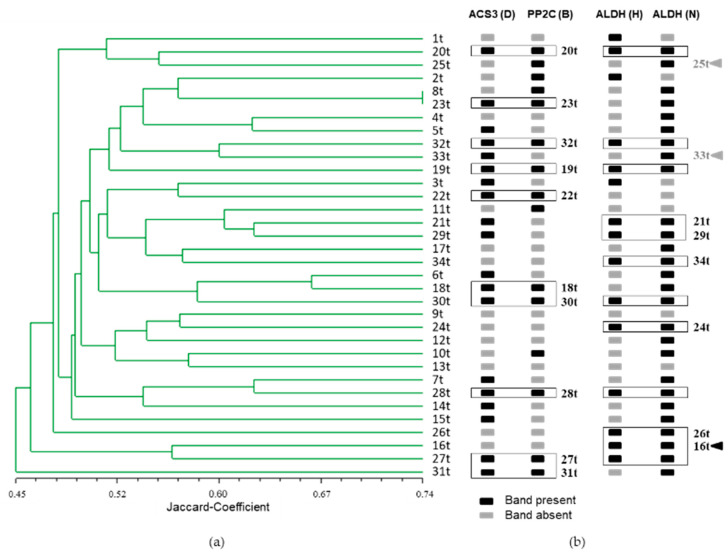
Clustering of the 34 potato varieties based on the genetic similarity obtained by SSR analyses and selection with SSR markers. (**a**) UPGMA dendrogram of the 34 potato varieties (1t = most drought tolerant, 34t = most sensitive) based on all 75 SSR primer combinations derived from candidate genes for drought tolerance, Mantel coefficient r = 0.62; (**b**) Selection by applying four SSR markers associated with drought sensitivity. Black box= band is present, grey box= band is absent. The varieties are ordered according to the UPGMA dendrogram. Boxed varieties are selected by the presence of two marker bands either in the combination HRO_ACS3_D and HRO_PP2C_1_B or the combination HRO_ALDH_H and HRO-ALDH_N. Black Arrow head = falsely negative selected; Grey arrow head = falsely positive selected.

The banding pattern obtained by 75 polymorphic SSR primer combinations derived from candidate genes for drought tolerance was used to estimate the genetic similarity among 34 potato cultivars based on the Jaccard’s coefficient (Figure 6). The clustering of the 34 potato cultivars as shown in the UPGMA dendrogram did not reveal any grouping corresponding to drought tolerance. However, when the SSR markers HRO_ACS3_D, HRO_PP2C_B, HRO_ALDH_H and HRO_ALDH_N associated with drought sensitivity were used for marker-assisted selection, 15 out of 17 drought-sensitive cultivars were correctly identified. Only 33t and 25t were not detected as drought-sensitive and only 16t was falsely identified as drought sensitive, even though the ranking put this cultivar in the drought tolerant group. The marker-assisted selection was performed in the way that a cultivar was considered drought-sensitive if both bands, HRO_ACS3_D as well as HRO_PP2C_B, were present or if the cultivar was characterized by the presence of both bands H and N produced by the primer combination HRO_ALDH (Figure 5). The band HRO_ALDH_N had only been weakly associated with drought tolerance using the Fisher’s exact test. However, it proved to be essential for the selection of drought sensitive cultivars using the three primer combinations HRO_ACS3, HRO_PP2C and HRO_ALDH.

The alleles for drought sensitivity detected by the three SSR primer combinations should be eliminated by breeding to obtain more drought tolerant cultivars. Including the two additional primer combinations HRO_ETRTF_5a and HRO_PARG_1A in the selection procedure did not improve the selection efficiency, even though most drought sensitive cultivars (14) contained at least one of these negative alleles, six even both negative alleles detectable by these two SSR primer combinations (Supplementary Table S8).

## 4. Discussion

### 4.1. Development of Drought Tolerance Associated SSR Markers for Potato

In the present study, 75 functional SSR markers were developed starting from 103 potential candidate genes for drought tolerance derived from own transcriptome analyses [39] and literature. SSR markers derived from five candidate genes (aldehyde dehydrogenase, PGSC0003DMG400034597; 1-aminocyclopropane-1-carboxylate synthase 3, PGSC0003DMG400021426; ethylene responsive transcription factor, PGSC0003DMG400002185; poly (ADP-ribose) glycohydrolase, PGSC0003DMG 400029361; protein phosphatase 2C, PGSC0003DMG400011321) were significantly associated with drought sensitivity. These five SSR-markers identified allelic differences between potato cultivars that are crucial for sensitivity towards drought. Selection for drought tolerance using three of the significantly associated SSR markers allowed in 91 % of the cases a correct grouping into drought tolerant or drought sensitive potato cultivars. Now, it would be interesting to test these SSR markers in a larger international potato association panel.

Our approach to derive SSR markers from candidate genes for drought tolerance made mostly use of the presence of interrupted pure, compound, interrupted compound, complex and interrupted complex repeats. This strategy differed from previous studies, which concentrated mostly on the use of pure as well as compound and complex SSRs [45,46,47,48,49,50,51]. Our results showed that the use of SSR markers derived from potential candidate genes worked well and can be used to detect genetic diversity in potato cultivars. In addition, the number of repeats was lowered from eight to six as compared to previous studies, which still resulted in highly polymorphic banding patterns. Comparing the locations for all derived SSR primer combinations versus only the polymorphic SSR markers the percentage of polymorphic SSR markers located in exon regions was considerably reduced to only 15% compared to 28% for all SSR primer combinations. On the other hand, the percentages of polymorphic SSR markers derived from 5′-UTR, 3′-UTR and intergenic regions and were slightly increased with 18%, 11% and 11%, respectively.

### 4.2. Role of PP2C as Part of the ABA Signaling Pathway

Our SSR analyses showed a significant association of drought sensitivity with allelic variations in PP2C (PGSC0003DMG400011321) located on LG1. The interrupted complex SSR (GAA)_k_A(AT)_m_CAT(GAT)_n_ used for detection is located in exon 3 of the gene. Changes in the repeat numbers will have an effect on the encoded protein. PP2Cs are known drought responsive genes in potato. Induction of expression was shown for drought exposed moderately drought tolerant native Andean potato clones [19]. In addition, comparing transcriptome profiles of drought treated and re-watered plants of the potato strain Ningshu4 also revealed a contrasting expression pattern for PP2C 30 (PGSC0003DMG400027174, located on LG5) [38]. In diploid potato, a different PP2C (PGSC0003DMG400027196, also located on LG5) was identified as a central point in the gene regulatory network [64]. PP2Cs are part of the ABA signaling pathway, which consists of three major players: ABA receptors, PP2C and SnRK2 [65]. The ABA signaling pathway leads to phosphorylation of SnRK2 by inhibiting dephosphorylation by PP2C in the presence of ABA. Auto- and trans-activation of SnRK2 then results in phosphorylation and activation of downstream transcription factors [66]. SnRK2 modulates gene expression by targeting basic-zipper class transcription factors (ABI5) for arrest of seed germination, but also phosphorylates SLAC1 (slow anion channel-associated 1) to induce stomatal closure under limiting water conditions [67,68,69].

Arabidopsis mutants of PP2C like *ABI1* (ABA insensitive 1) and its homolog *ABI2* revealed PP2C role as negative regulator of ABA trigged responses such as inhibition of germination, root elongation, and induction of stomatal closure [70]. Furthermore, PP2C mutant *sag113* (*senescence associated gene 113)* showed higher ABA sensitivity for stomatal movement and reduced leaf senescence [71]. In wheat, a genome-wide study of the *TaPP2C* gene family identified 257 homoeologs of 95 *TaPP2C* genes distributed over the A, B and D sub genomes [72]. Phylogenetic analyses separated the *TaPP2C* genes into 13 groups A-M. TaPP2Cs of group A were upregulated by ABA treatment and showed interaction with TaSnRK2.1 and TaSnRK2.2. Overexpression of *TaPP2C135* in Arabidopsis increased tolerance of germinating seedlings towards ABA [72].

Enhanced ABA synthesis during drought stress induces signaling cascades that activate plant stress responses and regulate the water status of plants mainly through management of stomatal aperture and maintenance of root growth [73]. Therefore, allelic variations in PP2C (PGSC0003DMG400011321) as part of the ABA signaling pathway could play a major role in drought tolerance in potato and further functional studies of the different allelic compositions are required to better understand the causes behind drought tolerance in potato.

### 4.3. Damage Control of Drought Induced Reactive Oxygen

The production of reactive oxygen species (ROS) under drought stress leads to oxidative stress and an excessive accumulation of oxidized toxic compounds such as aldehydes [74]. ALDHs act as aldehyde scavengers by irreversibly oxidizing aldehydes into the respective carboxylic acids [75]. Our SSR analyses in potato showed that one allelic difference in ALDH (PGSC0003DMG400034597, LG9) was significantly associated with drought sensitivity and another one also relevant for selection. This ALDH demonstrates the highest similarity in *Arabidopsis thaliana* to AT1G54100 (84.1% similarity according to Spud DB), which is annotated as ALDH family 7 member B4. In *A. thaliana,* members of *ALDH* gene families 3 and 7 (*ALDH3I1* and *ALDH7B4*), show the strongest transcriptional activation under osmotic stress [76]. Cytosolic overexpression of *ALDH7B4* confers enhanced detoxification and turgor response, demonstrating its importance under stress conditions [77]. As ALDH7B4 plays a major role in drought tolerance in *A. thaliana*, association of variation in this enzyme with drought sensitivity in potato suggests a functional relevance, which needs to be further investigated. In addition, the upregulation of this ALDH (PGSC0003DMG400034597) under water-limited conditions in greenhouse and field trials supports its importance in drought stress response in potato [39].

### 4.4. Damage Repair of DNA under Drought

Drought stress can lead to DNA lesions including double strand breaks (DSBs) as a major form [78,79,80]. DNA damage surveillance and repair processes have to recruit a number of repair proteins including poly (ADP-ribose) polymerases (PARPs), which recognize DNA SSBs and DSBs, assemble DNA repair complexes and auto-PARylate [81,82]. As the PARylation process is reversible, ADP-ribose chains are eventually hydrolyzed by the enzyme poly (ADP-ribose) glycohydrolase (PARG) [83]. As DNA damage repair is required for recovery of plants from drought stress, our SSR results implicating a role of allelic variation in PARG (PGSC0003DMG400029361, LG12) with drought tolerance in potato seem relevant. PARG (PGSC0003DMG400029361) was also used as one of the 27 predictors in forecasting drought tolerant potato based on transcripts and metabolites [31].

The *PARG* gene (PGSC0003DMG400029361) in potato shows the highest similarity on protein level to PARG1 (AT2G31870) (73.2% similarity according to Spud DB), one of two *PARG* genes present in tandem array on chromosome 2 in Arabidopsis [84]. An Arabidopsis *parg1* mutant exhibits loss of drought, osmotic, and oxidative stress tolerance, coupled with enhanced cell damage and loss of stomatal closure under drought stress [85].

### 4.5. Role of ACC Synthase and Ethylene Response Factors in Drought Tolerance

Ethylene represents a small two carbon gaseous hormone, which is involved in abiotic and biotic stress reactions, seed germination, fruit ripening, cell elongation, cell fate, leaf abscission and senescence [86]. Synthesis of ethylene involves two enzymes, 1-aminocyclopropane-1-carboxylic acid (ACC) synthase (ACS) and ACC-oxidase (ACO) [87,88].

For potato, we identified allelic differences in ACC synthase 3 (*StACS3*, PGSC0003DMG400021426, located on LG2) to be significantly associated with drought sensitivity. StACS3 shows the highest similarity to the Arabidopsis homolog AtACS8 (85% similarity according to Spud DB) encoded by *AT4G37770*. In roots, higher expression levels have been observed for this gene under drought conditions (TAIR Arabidopsis eFP Browser [89]). In Arabidopsis, the *ACS* gene family originally contained 12 annotated members *ACS1-12* [90]. A large scale of single as well as multiple mutants (*acs1*, *acs2*, *acs4*, *asc5*, *acs6* and *acs9*) demonstrated interactions between different ACS isoforms [91]. The ACS family is grouped into three types I-III based on the C-terminal end [92]. *StACS3* in potato represents a type II ACS as ACC synthases encoded in Arabidopsis by *AtACS4*, *AtACS5*, *AtACS8*, *AtACS9* and *AtACS11* [93]. These ACS are characterized by a shorter conserved C-terminal domain (TOE domain) around a serine as one potential phosphorylation site. Phosphorylation of ACS of type II by casein kinase 1.8 (CK1.8) leads to interaction with the E3 ubiquitin ligase Ethylene Overproducer 1 (ETO1) and ETO1-like (EOL), which results in degradation of ACS via 26S proteasome [88]. Binding of ACS by 14-3-3 protein represses this interaction and destabilizes ETO1/EOL [88]. In maize, *ZmACS6*-deficient mutants showed an inhibition of drought-induced leaf senescence, an increased transpiration rate and higher CO_2_ assimilation under drought stress conditions [94]. Variations in StACS3 may be interesting for drought tolerance breeding in potato as ACS represents a potentially rate-limiting enzyme in ethylene biosynthesis. Ethylene invokes responses in both young and mature potato leaves [18].

Ethylene acts as central integrator linking and reprogramming complex stress-adaptive signaling cascades through activation of downstream ERFs [95]. ERFs show conserved binding preference for the dehydration responsive element (DRE) (CCGAC) or the GCC motif (GCCGCC) in promoter sequences that control expression of abiotic and biotic stress-responsive genes [96,97,98,99]. Some ERFs can also bind to both motives, DRE and GCC.

A genome-wide study of ERFs in potato identified 155 genes, which could be mainly distributed into two groups, the CBF/DREB and the ERF group [100]. Comparison of RNA-seq data revealed that most *ERF* genes are controlled by abiotic stress conditions (drought, salt, low temperature, ABA treatment). Overexpression of *StDREB1*/*StERF186*, as well as of *StDREB2*/*StERF137*, improves tolerance to salt stress in transgenic potatoes [101,102]. Overexpression of the tomato jasmonate and ethylene response factor 1 (JERF1) results in enhanced drought tolerance and ABA biosynthesis in tobacco and rice [103]. The *ERF* gene (PGSC0003DMG400002185) significantly associated with drought sensitivity in our SSR study corresponds to *StERF8* [100]. As the SSR (CCA)_n_(ACA)_k_ is located in exon 1, the observed allelic variation will have an effect on the amino acid sequence of the encoded transcription factor. Further studies will be necessary to reveal the specific effects of these allelic variations of *StERF8* for drought tolerance in potato.

## 5. Conclusions

In potato, the candidate gene approach proved to be efficient to derive new functional SSR primer combinations and to identify SSR markers detecting allelic variations significantly associated with drought tolerance or sensitivity. Future testing of these SSR-markers in a larger panel of potato cultivars from all over the world will give a better idea about their versatility. Our association studies identified allelic differences in five of the candidate genes (aldehyde dehydrogenase, PGSC0003DMG400034597; 1-aminocyclopropane-1-carboxylate synthase 3 PGSC0003DMG400021426; ethylene responsive transcription factor, PGSC0003DMG400002185; poly (ADP-ribose) glycohydrolase, PGSC0003DMG400029361; protein phosphatase 2C, PGSC0003DMG400011321) as significantly associated with drought sensitivity. Further investigations will elucidate the role of these allelic variations in drought response of cultivated tetraploid potato and will give a better insight into mechanisms involved in drought tolerance in Solanaceae. The identified SSR markers are now available for genetic diversity studies and for marker-assisted selection in potato breeding programs. Interestingly, in our investigation breeding for drought tolerance in potato seems to require elimination of certain alleles from drought sensitive cultivars.

## Figures and Tables

**Table 1:** Scoring pattern of SSR markers significantly associated with drought tolerance in potato.

Primer\Cultivar	1	2	3	4	5	6	7	8	9	10	11	12	13	14	15	16	17	18	19	20	21	22	23	24	25	26	27	28	29	30	31	32	33	34
HRO_ACS3_D	0	0	1	0	1	1	1	0	0	0	0	0	0	1	1	0	0	1	1	1	1	1	1	0	0	0	1	1	1	1	1	1	1	0
HRO_PP2C_1_B	0	1	0	0	0	0	0	1	0	1	1	0	0	0	0	0	0	1	1	1	0	1	1	0	1	0	1	1	0	1	1	1	0	0
HRO_ALDH_H	1	1	1	0	0	0	0	0	0	0	0	0	0	0	0	1	0	0	1	1	1	0	0	1	0	1	1	1	1	1	0	1	0	1
HRO_ETRTF_5a_D	0	1	1	0	0	0	0	1	0	0	0	0	0	0	1	0	0	0	1	1	0	1	1	1	1	1	0	1	0	0	1	1	1	0
HRO_PARGH_1A_B	0	0	0	0	1	0	0	1	0	0	0	1	0	0	0	0	0	0	1	1	1	1	1	0	0	1	1	1	1	0	0	0	0	1

**Table 2:** SSR Markers significantly associated with drought tolerance in potato.

SSR Marker	Size	LG	Gene ID PGSC0003	Transcript ID PGSC0003	Gene Annotation	*p*-Value
HRO_ACS3_D	173 bp	LG 2	DMG400021426	DMT400055203	1-aminocyclopropane-1- carboxylate synthase 3	*p* = 0.0366
HRO_PP2C_1_B	205 bp	LG 1	DMG400011321	DMT400029441	protein phosphatase 2C	*p* = 0.0366
HRO_ALDH_H	184 bp	LG 9	DMG400034597	DMT400085026	aldehyde dehydrogenase	*p* = 0.0366
HRO_ETRTF_5a_D	217 bp	LG 11	DMG400002185	DMT400005585	ethylene responsive transcription factor	*p* = 0.0366
HRO_PARG_1A_B	147 bp	LG 12	DMG400029361	DMT400075512	poly (ADP-ribose) glycohydrolase	*p* = 0.0324

## Data Availability

Genomic sequences used are available in EnsemblPlants (Potato Genome Sequencing Consortium 2011), NCBI, Phytozome, Sol Genomics Network and Spud DB Potato Genomics Resource.

## Supplementary Materials

The following are available online at https://www.mdpi.com/article/10.3390/genes12040494/s1, Table S1. Characterization of potato cultivars. Table S2. Field trials for ranking the potato cultivars using DRYM as drought stress index. Table S3. Overview over all 154 derived SSR primer combinations. Table S4. Overview of 19 AFLP primer combinations used in the UPGMA dendrogram (Figure S1). Table S5. Linkage group specific SSR primer combinations. Table S6. Selected 23 transcripts identified in comparative transcriptome analysis [39]. Table S7. Characterization of 75 polymorphic SSR primer combinations. Table S8. Association studies of the SSR markers applying the Fisher Exact Test in the potato association panel (1t most drought tolerant cultivar, 34t most drought sensitive cultivar). Figure S1. Population structure in the investigated association panel of 34 starch potato cultivars based on 54 linkage group specific SSR primer combinations and 19 AFLP primer combinations. The UPGMA dendrogram based on the Jaccard’s coefficient is shown. The potato cultivars are shown with the drought tolerance rank from 1t = most tolerant to 34t = most sensitive.
