# Supplementary material for: Unravelling Differences in Candidate Genes for Drought Tolerance in Potato (Solanum tuberosum L.) by Use of New Functional Microsatellite Markers
Source: Genes (Basel). 2021 Mar 28;12(4):494. doi: 10.3390/genes12040494 (PMC8067248; doi:10.3390/genes12040494)
Supplement: Supplementary file 1 [file genes-12-00494-s001.pdf]

Table S1. Characterization of potato cultivars

Cultivar identifier, cultivar name, breeding companies and drought tolerance rank (1t = sensitive, 34t = tolerant) of 34 mostly starch potato cultivars.

Cultivar ID	Cultivar	Breeder	DRYM	Rank
382	DESIREE		0,02191	11t
2673	ALEGRIA	NORIKA	-0,00944	22t
2674	MILVA	BERDING	-0,04122	31t
2675	SATURNA	EUROPLANT	0,03639	9t
2853	ULME	BAVARIA	0,01636	14t
2854	ELDENA	EUROPLANT	0,02836	10t
2855	EUROBRAVO	EUROPLANT	-0,04375	33t
2856	EUROFLORA	EUROPLANT	-0,05497	34t
2857	EURONOVA	EUROPLANT	0,00923	16t
2858	EURORESA	EUROPLANT	-0,04250	32t
2859	EUROSTARCH	EUROPLANT	-0,00722	21t
2860	EUROTANGO	EUROPLANT	-0,02249	27t
2861	KURAS	EUROPLANT	-0,03498	29t
2862	TOMENSA	EUROPLANT	-0,01314	23t
2863	TOMBA	EUROPLANT	0,00851	17t
2864	JUMBO	FIRLBECK	0,01377	15t
2865	LOGO	FIRLBECK	-0,02198	26t
2866	MAXI	FIRLBECK	-0,02845	28t
2867	POWER	FIRLBECK	0,05740	5t
2868	SOMMERGOLD	FIRLBECK	0,05960	4t
2869	JASIA	NIEHOFF	-0,02031	25t
2870	ALBATROS	NORIKA	0,05042	6t
2871	KARLENA	NORIKA	0,08962	2t
2872	KIEBITZ	NORIKA	0,01824	13t
2873	KOLIBRI	NORIKA	0,13705	1t
2874	KORMORAN	NORIKA	0,00216	20t
2875	MAXILLA	NORIKA	0,00684	18t
2876	PIROL	NORIKA	-0,02001	24t
2877	BURANA	SAKA	0,00627	19t
2878	GOLF	SAKA	0,02017	12t
2879	PRIAMOS	SAKA	0,08819	3t
2880	RAMSES	SAKA	-0,03601	30t
2881	SIBU	SAKA	0,04748	7t
2882	VERDI	SAKA	0,03717	8t

Supplementary Table S2: Field trials for ranking the potato cultivars using DRYM as drought stress index (DOI: 10.5447/ipk/2021/7)

No.	Location	Year	Replications	Number of plants/ replication	Treatments	
					Control	Stress
F1	MPI-MP Potsdam Golm	2011	4	8	329.76 mm irrigation and precipitation[1]	274.32 mm irrigation and precipitation[1]
F2	Potato Research Station Dethlingen	2011	2	31	50% field capacity [2] (optimal water supply)	(30% field capacity) + no irrigation
F3	MPI-MP Potsdam Golm	2012	4	8	360.88 mm irrigation and precipitation[1]	319.74 mm irrigation and precipitation
F4	JKI Groß-Lüsewitz	2012	2	6	234.5 mm irrigation and precipitation	rain-out shelter, watered once at the beginning (20 mm), no watering afterwards
F5	Potato Research Station Dethlingen	2012	2	31	50% field capacity [2] (optimal water supply)	30% field capacity + no irrigation

[1] Control plots: Drip-irrigation (Netafim) with 10 mm water during the night if plants showed signs of reduced turgor at noon.
Drought stress plots: Drip-irrigation if plants had signs of water deficit 2 h after sunrise

[2] Control plots: Irrigation if soil water content dropped below 50% field capacity.
Drought stress plots: 2 plots - Irrigation with a irrigation boom at 30% field capacity, 2 plots - no irrigation. Field capacity was estimated according to the irrigation advice of the Deutscher Wetterdienst, Braunschweig.

Table S3. Overview over all 154 derived SSR primer combinations

Primer combinations that showed signifcant association with drought tolerance are marked in green.

[1] Primer combinations were in an interrupted complex or an interrupted compound structure of the type (repeats)k X (repeats)n(repeats)m or (repeats)n X (repeats)m X exceeded 4 are shown in red.

[2] nd not determined, means Ta and cycle number was not optimized monomorphic primer combinations

No.	Candidate gene	Tested SSR primer combination	Reference (DMG, Phytozome)	Reference (DMT) (TROST)	Primary transcript	Location Gene (Ensemble Plants) SolTub 3.0	Gene size (in bp, Ensemble)	Location Gene (Phytozome)	Gene size (in bp, phytozome)	ORF size (in bp)	CDS (in bp)	LG	SSR Motif[1]	Class of microsatellite (Chambers & MacAvoy 2000)	Location of the SSR	Primer sequences in 5'-3' direction (without M13 tail)	T°a in °C[2]	Cycles[2]	Expected size in bp (without M13 tail)
1	ABA8'-hydroxylase (ABA8H) CYP707A2	HRO_ABA8H4_2	PGSC0003 DMG400007972	PGSC0003 DMT400020589	PGSC0003 DMT400020589	4:68244330-68248224	3895	chr04:60375592..60379486 forward	3895	3346	1428	4	$(GT)_2 (AT)_7$	Compound	Intron 6	CACGTGTCAAATTCTCCTGAAA GGCATTACCTTCCATCCTTTT	60	36	200
2	ABA 8'-hydroxylase (ABA8H4) CYP707A1	HRO_ABA8H4_3	PGSC0003 DMG400001960	PGSC0003 DMT400004951	PGSC0003 DMT400004951	8:47110633-47113366	2734	chr08:34327986..34330719 reverse	2734	2368	1389	8	$(TA)_7 (AT)_2$	Compound	3'-UTR	GGAAAGCTAAGAAGGGTCCAGA TGTTTACATTAACTCCTTTCTTTTCTT	nd	nd	181
3	Abscisic acid receptor PYL4	HRO_ABAPR4	PGSC0003 DMG400020607	PGSC0003 DMT400053120	PGSC0003 DMT400053120	3:35855161-35856066	906	chr03:29295065..29295970 reverse	906	606	606	3	$(TC)_3$ CTTT $(GAA)_3$	Interrupted compound	5'-UTR	TGTAAAGAGTACTCCATCCTCACC GAGGCGGTTTGTGGAAGTT	nd	nd	144
4	1-aminocyclopropane-1-carboxylate oxidase (ACO)	HRO_ACCO_1 (HRO_ACO_1)	PGSC0003 DMG400021476	PGSC0003 DMT400055332	PGSC0003 DMT400055332	2:17740757-17743016	2260	chr02:38908747..38911006reverse	2260	1838	960	2	$(AT)_8$	Pure	Intron 2	ACAGGTCAGAGGGATCTCCA TGTTTTAGACCACAAATTTCAAA	55	36	134
5	1-aminocyclopropane-1-carboxylate oxidase (ACO)	HRO_ACCO_4 (HRO_ACO_4)	PGSC0003 DMG400009719	PGSC0003 DMT400025148	PGSC0003 DMT400025148	9:49787843-49791601	3759	chr09:39308252..39312010 reverse	3759 (without UTR)	3759	837	9	$(GA)_{12}$	Pure	Intron 3	TGGCTCGCCTATATTTGACA TGAATTTTATAGAAACTTTTCTCTCCT	53	36	172
6	1-aminocyclopropane-1-carboxylate oxidase homolog (ACO homolog)	HRO_ACCOhom_1	PGSC0003 DMG400044665	PGSC0003 DMT400095094	PGSC0003 DMT400095094	10:45540344-45543554	3211	chr10:40921106..40924316 forward	3211 (without UTR)	3211	432	10	$(TG)_5 (AG)_3$	Compound	Intron 1	TCACACGTGATGTTCAGACAAG TGGCCGTGTAGAGTGAAGGT	nd	nd	204
7	1-aminocyclopropane-1-carboxylate synthase (ACS)	HRO_ACCS_2 (HRO_ACS_2)	PGSC0003 DMG400000284	PGSC0003 DMT400000771	PGSC0003 DMT400000771	12:3809539-3812003	2465	chr12:4302319..4304783 reverse	2465 (without UTR)	2465	1305	12	$(AT)_6$	Pure	Intron 2	ATGCCTTGCTTGTTCCAACT AAAGGTATCTTTGACTCTTTTCCCTA	60	36	139
8	1-aminocyclopropane-1-carboxylate synthase 2 (ACS2)	HRO_ACCS2_1 (HRO_ACS2_1)	PGSC0003 DMG400000193	PGSC0003 DMT400000553	PGSC0003 DMT400000553	1:72662888-72665831	2944	chr01:78802955..78805898 reverse	2944	2652	1461	1	$(TTA)_5$ T $(TAA)_9$	Interrupted compound	Intron 3	CAGTTCCTTTGGGGAATTTG CAAGAAAGAGCTTGCGTGAT	55	36	211
9	1-aminocyclopropane-1-carboxylate synthase 3 (ACS 3)	**HRO_ACCS3_1 (HRO_ACS3)**	PGSC0003 DMG400021426	PGSC0003 DMT400055203	PGSC0003 DMT400055203	2:42416137-42418380	2244	chr02:63671580..63673823 reverse	2244	1742	1410	2	$(AT)_9$	Pure	Intron 2	TCATGGTCTTCCTGCTTTCA TTTTGTCATGGCCTGCATTA	55/57	36	159
10	1-aminocyclopropane-1-carboxylate synthase 4 (ACS4)	HRO_ACCS4_1 (HRO_ACS4_1)	PGSC0003 DMG400021651	PGSC0003 DMT400055753	PGSC0003 DMT400055753	5:44070623-44073744	3122	chr05:53360564..53363685 forward	3122	2775	1143	5	$(ACT)_5$	Pure	Exon 1	GGTTCTTCTTATTGGTCGCAAA GAAAACCTCTCTAATAGCCAGCA	55	36	104
11	Aldehyde dehydrogenase family 7 member (ALDH)	**HRO_ADFMfor_ADHrev (HRO_ALDH)**	PGSC0003 DMG400034597	PGSC0003 DMT400085026	PGSC0003 DMT400085026	9:22591541-22597210	5670	chr09:38340472..38346141 reverse	5670 (without UTR)	5670	630	9	$(AT)_{12} (AG)_{12}$ ATA $(GA)_9$	Interrupted complex	Intron 1	TTCTTGTCCTGGATTGACCAC ATTCTAATTAATCGCTCTCTTTACA	54	36	177
12	Aldehyde dehydrogenase family 7 member (ALDH)	HRO_ADFMfor_ADHrevn1	PGSC0003 DMG400034597	PGSC0003 DMT400085026	PGSC0003 DMT400085026	9:22591541-22597210	5670	chr09:38340472..38346141 reverse	5670 (without UTR)	5670	630	9	$(AT)_{12} (AG)_{12}$ ATA $(GA)_9$	Interrupted complex	Intron 1	TTCTTGTCCTGGATTGACCAC GGAAGTATTGAAACTAATATCAT GGAA	nd	nd	216
13	Aldehyde dehydrogenase family member 7 A1 (ALDH)	HRO_ADFM_ADHrev	PGSC0003 DMG400034597	PGSC0003 DMT400085026	PGSC0003 DMT400085026	9:22591541-22597210	5670	chr09:38340472..38346141 reverse	5669 (without UTR)	5670	630	9	$(AT)_{12} (AG)_{12}$ ATA $(GA)_9$	Interrupted complex	Intron 1	TTCTTGTCCTGGATTGACCAC ATTCTAATTAATCGCTCTCTTTACA	nd	nd	177
14	Aldehyde dehydrogenase family member 7 A1 (ALDH)	HRO_ADH	PGSC0003 DMG400034597	PGSC0003 DMT400085026	PGSC0003 DMT400085026	9:22591541-22597210	5670	chr09:38340472..38346141 reverse	5669 (without UTR)	5670	630	9	$(AT)_{12} (AG)_{12}$ ATA $(GA)_9$	Interrupted complex	Intron 1	TTCTTGTCCTGGATTGACCA ATTCTAATTAATCGCTCTCTTTACA	nd	nd	195
15	Aldehyde dehydrogenase family member 7 A1 (ALDH)	HRO_ADHfor_ADFMrev	PGSC0003 DMG400034597	PGSC0003 DMT400085026	PGSC0003 DMT400085026	9:22591541-22597210	5670	chr09:38340472..38346141 reverse	5669 (without UTR)	5670	630	9	$(AT)_{12} (AG)_{12}$ ATA $(GA)_9$	Interrupted complex	Intron 1	TTCTTGTCCTGGATTGACCA TGAGCTCACTTTGGTTAAGGAAG	nd	nd	234
16	Aldehyde dehydrogenase family member 7 A1 ALDH)	HRO_ADFMfor_ADHrevn2	PGSC0003 DMG400034597	PGSC0003 DMT400085026	PGSC0003 DMT400085026	9:22591541-22597210	5670	chr09:38340472..38346141 reverse	5669 (without UTR)	5670	630	9	$(AT)_{12} (AG)_{12}$ ATA $(GA)_9$	Interrupted complex	Intron 1	TTCTTGTCCTGGATTGACCAC ATTAATCGCTCTCTTTACA	nd	nd	189
17	Ascorbate peroxidase (APX)	HRO_APX_2	PGSC0003 DMG401001731	PGSC0003 DMT400004360	PGSC0003 DMT400004360	9:6599287-6603984	4698	chr09:3225908..3230605 forward	4698	4207	753	9	$(TA)_3$ C $(TA)_3$	Interrupted pure	3'-UTR	AAAGGTCTAAAGACCAACTTTTCA TGAGATTCTGACGGACACTAGC	57	36	174
18	Apyrase (APY)	HRO_APY_2	PGSC0003 DMG400007656	PGSC0003 DMT400019809	PGSC0003 DMT400019809	8:9072734-9079782	7049	chr08:8146602..8153650 reverse	7049	6340	1419	8	$(CT)_4$ G $(TC)_3$	Interrupted compound	Intron 6	CAATGCTGCTGTTTCTGTCC TTTGGGGAAAACAGAAGTTGA	nd	nd	190
19	Apyrase (APY)	HRO_APY_3	PGSC0003 DMG400020578	PGSC0003 DMT400053021	PGSC0003 DMT400053021	9:59667071-59670753	3683	chr09:50849737..50853419 reverse	3683 (without UTR)	3683	849	9	$(AT)_6$ A $(ATT)_2$	Interrupted compound	Intron 4	TTTCTTTGATATGGCTTCTGAGG TGATGCTTTTGGATCAACAATA	nd	nd	190
20	Apyrase 2 (APY2)	HRO_APY2_1	EU125182.1 (NCBI)	(96,6 % ID with PGSC0003 DMG400020578 - but only 59 bp	(96,6 % ID with PGSC0003 DMG400020578 - but only 59 bp	12:59342085-59343791 (Ensembl:BLAST Seq 99,2 % ID) chr12:65973640..65977563 (Phytozome (BLAST)	3909 (NCBI)	-	-	-	-	12	$(TA)_7$ $(CAA)_2$	Compound	unknown	GGACAAAAGACTATTCATGCTTCA TGGTGGAAAAGACGTGAAAA	nd	nd	161
21	Apyrase 3 (APY3)	HRO_APY3_1	PGSC0003 DMG400043318	PGSC0003 DMT400093747	PGSC0003 DMT400093747	2:16368634-16370254	1621	chr02:39965172..39966792 forward	1621 (without UTR)	1621	720	2	$(TA)_5$	Pure	Intron 1	TCTTTTGGGAACTAATTATGACATC AACCTCCCAAAATACATGGAC	nd	nd	213
22	Auxin response factor 6 (ARF6)	HRO_ARF6_1	PGSC0003 DMG400028826	PGSC0003 DMT400074168	PGSC0003 DMT400074168	12:5941093-5948789	7697	chr12:5852473..5860169 reverse	7697	6821	2655	12	$(CT)_2 (TAAAT)_2 (GAA)_2$	Complex	Intron 6	CCTTGTACTGGCGGTTAAAA TGAATCCACGTCAGAGGAAA	55	36	219
23	Bacterial spot disease resistance protein 4 (BS4)	HRO_BSDRP4_5A	PGSC0003 DMG400002427	PGSC0003 DMT400006231	PGSC0003 DMT400006231	6:11390359-11397114	6756	chr06:14395786..14402541 forward	6756	6236	3594	6	$(TCT)_4$ GTG $(AGT)_2$	Interrupted compound	Exon 1	TGATCTCATCCGATCCATACA GGGTATCTTCGCCTCTGAAA	55	36	132
24	Bacterial spot disease resistance protein 4 (BS4)	HRO_BSDRP4_5B	PGSC0003 DMG400002427	PGSC0003 DMT400006231	PGSC0003 DMT400006231	6:11390359-11397114	6756	chr06:14395786..14402541 forward	6756	6236	3594	6	$(AT)_6$ A $(CT)_2$	Interrupted compound	Intron 2	TCAAGAACTTGAAGTGCAAAAA CAACTTAAATGACTTGTGCCTCA	nd	nd	147
25	Bacterial spot disease resistance protein 4 (BS4)	HRO_BSDRP4_5C	PGSC0003 DMG400002427	PGSC0003 DMT400006231	PGSC0003 DMT400006231	6:11390359-11397114	6756	chr06:14395786..14402541 forward	6756	6236	3594	6	$(TTTA)_5$ T $(TA)_2$	Interrupted compound	Intron 3	GCGGAAATTTAAATAAAACATGC TCAACGTGTGAAATGAGAAATTG	55	36	158
26	Bacterial spot disease resistance protein 4 (BS4)	HRO_BSDRP4_9C	PGSC0003 DMG400031476	PGSC0003 DMT400080813	PGSC0003 DMT400080813	11:1492867-1496748	3882	-	-	-	-	11	$(CT)_2 (TA)_3$ T $(TAA)_2$	Interrupted complex	unknown	CGGTAACTCTGCCTATGAAGG TGTAAGAGAAATGTCGTGTCACC	55	36	152
27	Bacterial spot disease resistance protein 4 (BS4)	HRO_BSDRP4_9A	PGSC0003 DMG400031476	PGSC0003 DMT400080813	-	11:1492867-1496748	3882	-	-	-	-	11	$(CT)_2 (TG)_2 (ATC)_2$	Complex	unknown	TGGACCAAAAATTGGATTGC TGCTATATTCTCCCGGATGC	nd	nd	143
28	BED finger-NBS-LRR resistance protein	HRO_BFNBLRP_2C	PGSC0003 DMG400013736	PGSC0003 DMT400035714	PGSC0003 DMT400035714	12:6955533-6960815	5283	chr12:6866913..6872195 reverse	5283	3802	2697	12	$(TA)_2$ GACTA $(TC)_2$ ATTC $(TTTA)_2$	Interrupted complex	3'-UTR	TTTGCAGTTCGTGTGATTCAG TGAGGAGTTGAGTTGCCTTTT	60	34	175
29	BED finger-NBS-LRR resistance protein	HRO_BFNBLRP_2A	PGSC0003 DMG400013736	PGSC0003 DMT400035714	PGSC0003 DMT400035714	12:6955533-6960815	5283	chr12:6866913..6872195 reverse	5283	3802	2697	12	$(GT)_2$ TTT $(TG)_2 (GA)_3$	Interrupted complex	5'-UTR	CATGGAAATTGTTGGTGCAG CCGAGCTTTAAGCAATTCCA	nd	nd	154
30	BED finger-NBS-LRR resistance protein	HRO_BFNBLRP_2B	PGSC0003 DMG400013736	PGSC0003 DMT400035714	PGSC0003 DMT400035714	12:6955533-6960815	5283	chr12:6866913..6872195 reverse	5283	3802	2697	12	$(GA)_4$ G $(CA)_2$	Interrupted compound	Exon 1	GGGTGTGGGTAAGACATCCA CCTTCTGCAATTTCTGGACA	nd	nd	125
31	Beta-amylase (BA)	HRO_BA_1	PGSC0003 DMG400000169	PGSC0003 DMT400000485	PGSC0003 DMT400000485	1:72104919-72114159	9241	chr01:78244986..78254226 reverse	9241	7195	2088	1	$(GAG)_2$ AAA $(GAA)_3$ $(GA)_3$A $(GAA)_2$	Interrupted complex	Intron 5	AGAGTCGCGAAAGAAGAGCA CGGGTGGTTAAAAGGCAATA	55/57	36	213
32	Beta-amylase (BA)	HRO_BA7_1	PGSC0003 DMG400026199	PGSC0003 DMT400067405	PGSC0003 DMT400067405	7:47346058-47350374	4317	chr07:45794514..45798195 reverse	3682 (without UTR)	3682	1764	7	$(GTT)_3 (GT)_3$	Compound	Intron 1	TGTACCTGTTTTGAGTCGAGGA AATCATTCAACGCGCACATA	nd	nd	208
33	Beta-D-glucan exohydrolase (BDGEH)	HRO_BDGEH_1A	PGSC0003 DMG402005942	PGSC0003 DMT400015224	PGSC0003 DMT400015224	6:54345177-54347242	2066	chr06:49595917..49597982 reverse	2066	1595	1116	6	$(TC)_3$ C $(ATG)_2$ CTACA $(AG)_2$	Interrupted complex	Intron 4	AGAAGGATCCACAGTTTTCTTGA TTCGTGGGCGATTAATTGTT	nd	nd	161
34	Beta-D-glucan exohydrolase (BDGEH)	HRO_BDGEH_1B	PGSC0003 DMG402005942	PGSC0003 DMT400015224	PGSC0003 DMT400015224	6:54345177-54347242	2066	chr06:49595917..49597982 reverse	2066	1595	1116	6	$(GT)_2 (TA)_2$ CATGG $(AT)_4$	Interrupted complex	Intron 1	GGCTTGTAATCCGGTCAATG AGCTCAGCTGCTCATTCAGA	57	36	138
35	Betaine aldehyde dehydrogenase (BADH)	HRO_BADH_1	PGSC0003 DMG400033028	PGSC0003 DMT400083025	PGSC0003 DMT400083025	6:52130166-52135413	5248	chr06:47094788..47100035 reverse	5248	4904	1515	6	$(T)_{20}$	Pure	Intron 1	TGAAGAAATCATCGGTACGTCA CCGGAATATCCCCTGAAAAT	nd	nd	185
36	Betaine aldehyde dehydrogenase (BADH)	HRO_BADH_2	PGSC0003 DMG400033028	PGSC0003 DMT400083025	PGSC0003 DMT400083025	6:52130166-52135413	5248	chr06:47094788..47100035 reverse	5248	4904	1515	6	$(GT)_3$ CA $(GT)_4$	Interrupted pure	Intron 9	TGCAACATCACGGCTTATAG ACACTGCCAAGCACGATATG	55	36	195
37	Betaine aldehyde dehydrogenase (BADH)	HRO_BADH_3	PGSC0003 DMG400033028	PGSC0003 DMT400083025	PGSC0003 DMT400083025	6:52130166-52135413	5248	chr06:47094788..47100035 reverse	5248	4904	1515	6	$(CTGAT)_4$	Pure	Intron 14	TGTTTCATTTTCAATGCACGA TAAAAAGCCCATGGTTCAGC	nd	nd	159
38	Constitutive Triple Response1 (CTR1)	HRO_CTR1K	PGSC0003 DMG400008384	PGSC0003 DMT400021612	PGSC0003 DMT400021612	10:53333706-53352295	18590	chr10:46142884..46161473 reverse	18590	16948	2379	10	$(TA)_2 (TAT)_5$	Compound	Intron 5	GCTTTCTTAACATGAAAACCTCA TTCAACCACAAAGCGCAATA	50	36	197

39	Dehydration responsive element binding factor 1 (DREB1)	HRO_DREB1_1	PGSC0003 DMG400012525	PGSC0003 DMT400032622	PGSC0003 DMT400032622	8:110755-112152	1398	chr08:786238..787635 reverse	1398	717	717	8	$(AAAAG)_3$	Pure	3'-UTR	TGTCTTTGTGGAGTTACAGTATTTGA<br>AATAGGGACGGAGGGAGTACA	nd	nd	148
40	Delta 1-pyrroline-5-carboxylate synthetase (P5CS)	HRO_D1P5CS_1	PGSC0003 DMG402026767	PGSC0003 DMT400068829	PGSC0003 DMT400068829	6:15776837-15786259	9423	chr06:14263202..14272624 reverse	9423	8928	2154	6	$(TTA)_{10}$	Pure	3'-UTR	CAAGGGGATCTGGACAAGTT<br>CATCAAAATGCGCTTTACCA	55/57	36	187
41	Delta 1-pyrroline-5-carboxylate synthetase (P5CS)	HRO_D1P5CS_2	PGSC0003 DMG402026767	PGSC0003 DMT400068829	PGSC0003 DMT400068829	6:15776837-15786259	9423	chr06:14263202..14272624 reverse	9423	8928	2154	6	$(CT)_5$ T $(TA)_2$	Interrupted compound	Intron 1	CGGGCATTTGTAAAGGATGT<br>CGGCAGTTCCAACCTTGTAT	57	36	168
42	DNA-binding protein 4 (DBP4)	HRO_DBP4_1	PGSC0003 DMG400008391	PGSC0003 DMT400021625	PGSC0003 DMT400021625	10:53176675-53181319	4645	chr10:45985853..45990497 reverse	4645	4303	657	10	$(CA)_2$ AA $(AT)_{18}$ $(TA)_2$	Interrupted complex	Intron 2	GGCCATGCTTGGAGAAAATA<br>TGCATGTCTTTCCACCTTCA	nd	nd	216
43	EIN2 targeting protein 1 (ETP1)	HRO_ETP1_1	PGSC0003 DMG400028237	PGSC0003 DMT400072568	PGSC0003 DMT400072568	10:56055611-56057397	1787	chr10:48864789..48866575 forward	1787	1287	1287	10	$(GAA)_2$ A $(TG)_2$TA $(GT)_2$ T $(GTT)_2$	Interrupted complex	Exon 1	GACCATTCTGGAAGCCAATG<br>GCTTCCCTTAGGTCAACCAC	nd	nd	187
44	EIN2 targeting protein 1 (ETP2)	HRO_ETP2_2	PGSC0003 DMG400020921	PGSC0003 DMT400053922	PGSC0003 DMT400053922	10:40868395-40869651	1257	chr10:21681386..21682642 forward	1257	1257	1257	10	$(GAG)_2$ CTAATT $(CA)_2$TGGA $(TCC)_2$	Interrupted complex	Exon 1	CATCTGAAAATAGCAGCCAATG<br>CCAATTCGTGATTGCAGAGA	nd	nd	202
45	EIN3-binding F-box protein 1 (EBF1)	HRO_EBF1_1a	PGSC0003 DMG400030928	PGSC0003 DMT400079445	PGSC0003 DMT400079445	7:4622738-4626267	3530	chr07:3656635..3660164 forward	3530	2579	1929	7	$(CT)_3$ T $(CAA)_3$	Interrupted compound	Exon 2	AGCTGCTGCACTCCTTTCAC<br>ATGTTGGCTCTTGGTCATGC	55	36	177
46	EIN3-binding F-box protein 1 (EBF1)	HRO_EBF1_2	PGSC0003 DMG400002914	PGSC0003 DMT400007545	PGSC0003 DMT400007545	12:2844416-2848113	3698	chr12:2844416..2848113 reverse	3698	2821	1914	12	$(AT)_2$ $(TGTC)_2$ GA $(TGT)_2$	Interrupted complex	3'-UTR	GGCCAAGCCCCTAGAATAAC<br>CTCATGTTGCTCCTCGAAAG	55	38	181
47	Ethylene insensitive 2 (EIN2)	HRO_EIN2_1A	PGSC0003 DMG400021547	PGSC0003 DMT400055484	PGSC0003 DMT400055484	9:5550240-5558623	8384	chr09:4040363..4048746 forward	8384	6202	3906	9	$(TC)_7$ $(AT)_2$ $(GT)_9$	Complex	Intron 4	AGAGTGCATTTGCATTGATGA<br>TTACAGATGTGCCTCCGTTCT	55	36	183
48	Ethylene insensitive 3 (EIN3)	HRO_EIN3_1A	PGSC0003 DMG400005914	PGSC0003 DMT400015145	PGSC0003 DMT400015145	6:54305277-54308543	3267	chr06:49634616..49637882 forward	3267	1800	1800	6	$(TA)_3$ $(AT)_3$	Compound	5'-UTR	TGTGCTTTTGCTTAAAGATTGG<br>CCCCCAAACAGAAATCTGAA	nd	nd	202
49	Ethylene insensitive 4 (EIN4)	HRO_EIN4_1	PGSC0003 DMG400023402	PGSC0003 DMT400060160	PGSC0003 DMT400060160	5:50783752-50787902	4150	chr05:59809871..59813239 reverse	3369 (without UTR)	3369	2292	5	$(TGA)_2$ $(AG)_2$ G $(GT)_2$	Interrupted complex	Exon 1	GGTGGTTGTTTGTGGGATTC<br>CCCATTTGAATGGAACATCA	nd	nd	225
50	Ethylene insensitive 5 (EIN5)	HRO_EIN5_1	PGSC0003 DMG400003725	PGSC0003 DMT400009550	PGSC0003 DMT400009550	4:70688095-70702726	14632	chr04:62819357..62833988 forward	14632	14121	3300	4	$(TG)_2$ $(TGA)_2$ ATG $(AT)_2$	Interrupted complex	Exon 19	TGCAGAAGTTGCAAAAGTTGA<br>GCTGCTTACAGCGATCATCA	nd	nd	219
51	Ethylene insensitive-like 1 (EIL1)	HRO_EIL1_1	PGSC0003 DMG400005915	PGSC0003 DMT400015148	PGSC0003 DMT400015148	6:54292149-54295237	3089	chr06:49647922..49651010 forward	3089	1833	1833	6	$(TG)_2$ GTT $(GAT)_3$ $(TA)_2$	Interrupted complex	Exon 1	GCTGAAGGAAGTGGAAGTGG<br>GTTGTTTTGCAGGATCGACA	nd	nd	197
52	Ethylene insensitive-like 2 (EIL2)	HRO_EIL2_1	PGSC0003 DMG400008712	PGSC0003 DMT400022477	PGSC0003 DMT400022477	1:6142379-6146461	4083	chr01:5271439..5275521 reverse	4083	1845	1845	1	$(ACCCTC)_2$ $(CT)_5$	Compound	5'-UTR	GGCACCAGATAGTTGGGAGA<br>AATTGCTACAGCCCCACAAC	55	36	132
53	Ethylene-inducing xylananse (EIX)	HRO_EIX_1A	PGSC0003 DMG400007876	PGSC0003 DMT400020366	PGSC0003 DMT400020367	12:1808690-1811450	2761	chr12:1808690..1810952 reverse	2263	1737	1737	12	$(GTT)_3$ $(TTC)_2$ $(GT)_2$	Complex	Exon 1	CCCAAAATAACCAGAAACCTG<br>CGGATCATTTACATGAGGACA	nd	nd	153
54	Ethylene-inducing xylananse (EIX)	HRO_EIX_1B	PGSC0003 DMG400007876	PGSC0003 DMT400020366	PGSC0003 DMT400020367	12:1808690-1811450	2761	chr12:1808690..1810952 reverse	2263	1737	1737	12	$(CT)_2$ TT $(GCT)_2$ TGA $(AT)_2$	Interrupted complex	Exon 1	GAAGCAGCTTAATTTGGGAAA<br>GAGGTTCCTGGAAAACATGG	nd	nd	108
55	Ethylene-inducing xylananse (EIX)	HRO_EIX_1D	PGSC0003 DMG400007876	PGSC0003 DMT400020366	PGSC0003 DMT400020367	12:1808690-1811450	2761	chr12:1808690..1810952 reverse	2263	1737	1737	12	$(ATTA)_2$ A $(AT)_4$ G $(TA)_2$	Interrupted complex	Intergenic region	TACTCAGAGGCGGAGCTAGG<br>AGACAGAGGCGGAGCTAAGA	nd	nd	194
56	Ethylene-inducing xylananse (EIX)	HRO_EIX_1D2	PGSC0003 DMG400007876	PGSC0003 DMT400020366	PGSC0003 DMT400020367	12:1808690-1811450	2761	chr12:1808690..1810952 reverse	2263	1737	1737	12	$(ATTA)_2$ A $(AT)_4$ G $(TA)_2$	Interrupted complex	Intergenic region	TCTTTACTCAGAGGCGGAGC<br>GGCGGAGCTAAGATTAAGAG	nd	nd	191
57	Ethylene-inducing xylananse (EIX)	HRO_EIX_1E	PGSC0003 DMG400007876	PGSC0003 DMT400020366	PGSC0003 DMT400020367	12:1808690-1811450	2761	chr12:1808690..1810952 reverse	2263	1737	1737	12	$(GT)_5$ GGGG $(CA)_2$	Interrupted compound	Intergenic region	TCTTAGCTCCGCCTCTGTCT<br>TTGCTCTTTGTTGACTTGAGGA	57	32	149
58	Ent-kaurenoic acid oxidase (KAO)	HRO_EKAO	PGSC0003 DMG400001823	PGSC0003 DMT400004596	PGSC0003 DMT400004596	1:67908155-67912635	4481	chr01:74093222..74097702 reverse	4481	4186	1494	1	$(TA)_8$	Pure	Intron 1	GAAGCATTTGTCTTCATTTAGTGAG<br>CACAACTTTGGCGTGAAAAA	nd	nd	215
59	Ethylene overproduction protein 1 (ETO1)	HRO_ETOP1_3	PGSC0003 DMG400015898	PGSC0003 DMT400041081	PGSC0003 DMT400041081	10:49687923-49692818	4896	chr10:42233573..42238468 reverse	4896	4071	2844	10	$(AT)_2$ CATAAT $(CCT)_3$	Interrupted compound	Exon 1	TATCTCTTCTTCCCCCATGC<br>CCGAGGAGTTCTTTGGTGTC	55	33	189
60	Ethylene receptor homolog (ETR homolog)	HRO_ETRHOM_2A	PGSC0003 DMG400028694	PGSC0003 DMT400073847	PGSC0003 DMT400073847	11:6479282-6486765	7484	chr11:277585..285068 forward	7484	5297	2304	11	$(GT)_4$ A $(TG)_6$	Interrupted compound	5'-UTR	TTTTTGGGGTTTTCTTGGTTT<br>AACCCCTCTTGCCAAAATTC	55	36	127
61	Ethylene response 1 (ETR1)	HRO_ETR1_1A	PGSC0003 DMG400007843	PGSC0003 DMT400020285	PGSC0003 DMT400020285	12:1111718-1121848	10131	chr12:1111718..1121848 reverse	10131	9594	2265	12	$(ATA)_2$ AAA $(ATA)_4$	Interrupted pure	Intron 6	GCAGACCCTTGGTTACCATC<br>CTCTGTGTGATCGTTGCTTTTC	55	36	180
62	Ethylene response 2 (ETR2)	HRO_ETR2_1	PGSC0003 DMG400016284	PGSC0003 DMT400041983	PGSC0003 DMT400041983	6:39789897-39794657	4761	chr06:35141052..35145812 forward	4761	2834	2286	6	$(CT)_2$ AGTTTT $(GTG)_2$ GTC $(GGGGT)_2$	Interrupted complex	5'-UTR	GGGTTTGCATAAGGGAAGTG<br>CCCCTCACATGCATTGGTAT	nd	nd	201
63	Ethylene response factor 114 (ERF114 = ERF80)	HRO_ERF114_1	PGSC0003 DMG400014240	PGSC0003 DMT400036934	PGSC0003 DMT400036934	3:57768986-57771196	2211	chr03:43459012..43461222 reverse	2211	1454	831	3	$(CTT)_3$ CAT $(CTT)_2$	Interrupted pure	Exon 2	TGTTGAGGTCCACCACTTGA<br>TGAATTTCGATGCTTCACCA	55	38	211
64	Ethylene response factor 12 (ERF12)	HRO_ERF12_1	PGSC0003 DMG400002350	PGSC0003 DMT400006041	PGSC0003 DMT400006041	6:38128095-38130080	1986	chr06:33905984..33907969 reverse	1986	1125	1125	6	$(TCAT)_2$ $(CA)_3$ AAG $(AAC)_2$	Interrupted complex	5'-UTR	AACCAAGGCGTTGTAGTCTCA<br>GGCTTAAAAAGGCTGAGTGG	nd	nd	209
65	Ethylene response factor 141 (ERF141)	HRO_ERF141	PGSC0003 DMG400028853	PGSC0003 DMT400074248	PGSC0003 DMT400074248	12:53036109-53038939	2831	chr12:59471842..59474672 reverse	2831	2339	1167	12	$(GA)_4$ $(TTTTTA)_2$	Compound	Intron 1	TGCCCAATGAACAACATAGC<br>CCTTTGCTTTCTCTGCTTCTG	55	38	199
66	Ethylene response factor 185 (ERF185)	HRO_ERF185_1	PGSC0003 DMG402013892	PGSC0003 DMT400036072	PGSC0003 DMT400036072	12:6424457-6432746	8289	chr12:6337488..6344126 forward	6639	6639	603	12	$(AG)_2$ $(TTC)_2$ $(GAA)_2$ $(GAAAAA)_2$	Complex	Intron 3	AGAAAATAGGGGTGGGATGG<br>TTTGAAACCCCCATCTTGAC	nd	nd	181
67	Ethylene response factor 4 (ERF)	HRO_ERF4_1	PGSC0003 DMG400016004	PGSC0003 DMT400041328	PGSC0003 DMT400041328	5:47293160-47294004	845	chr05:56538102..56538946 reverse	845	621	621	5	$(TAA)_2$ $(GAA)_3$ TTAC $(AG)_2$	Interrupted complex	Exon 1	CAATCTCCACTCCGATTTCC<br>TACCCAACCAAATCCTCGAT	53	34	197
68	Ethylene response sensor 1 (ERS 1)	HRO_ERS1_1	PGSC0003 DMG400031819	PGSC0003 DMT400081399	PGSC0003 DMT400081399	9:52922466-52930013	7548	chr09:44810065..44817612 reverse	7548	4323	1908	9	$(GCA)_5$ $(GC)_2$	Compound	Intergenic region	CAGGGACCAGGAGAGAAGC<br>TTAGATCCTCCCCTGCAACA	55	36	190
69	Ethylene response factor 1 (ERF1)	HRO_ERF	PGSC0003 DMG400026136	PGSC0003 DMT400067218	PGSC0003 DMT400067218	6:45876182-45877959	1778	chr06:44645977..44647754 reverse	1778	1267	591	6	$(TAA)_2$ TGAA $(AT)_3$ $(GT)_2$	Interrupted complex	Intron 1	CGAAATTCGCCATCCATTAC<br>AGATCCGCCTCTAACGTGTC	nd	nd	183
70	Ethylene responsive element binding protein 1 (EREBP1)	HRO_EREBP1_1	PGSC0003 DMG400029713	PGSC0003 DMT400076408	PGSC0003 DMT400076408	2:33632854-33633640	787	chr02:54888224..54889010 reverse	787	462	462	2	$(GTGTT)_4$ $(GTTTT)_2$ $(GT)_2$ $(TG)_3$	Complex	5'-UTR	TCCTCTGTAGTGACCCTCACG<br>CCGACGATTACGGGTGTC	57	36	200
71	Ethylene responsive element binding protein 1 (EREBP1)	HRO_EREBP_1a	PGSC0003 DMG400001067	PGSC0003 DMT400002750	PGSC0003 DMT400002750	12:4920261-4927464	4386	chr12:4913744..4920947 reverse	7204	6550	675	12	$(CTT)_2$ $(TC)_6$ CCC $(CTG)_2$	Interrupted complex	Intron 1	CCTGAAGTTTCAGTTGTCTCAGG<br>AGAAATGAGGTGCTGGAGGA	55	36	178
72	Ethylene responsive element binding protein C2 (EREBPC2)	HRO_EREBPC2_1	PGSC0003 DMG400016003	PGSC0003 DMT400041327	PGSC0003 DMT400041327	5:47308711-47310174	1464	chr05:56553653..56555116 reverse	1464	891	891	5	$(GTTG)_2$ AA $(TAT)_2$ GAAA $(GAATT)_2$	Interrupted complex	5'-UTR	TCCATGAAAACAACATCATCA<br>GCTCCAAGAAACCTCCATTG	55	36	202
73	Ethylene responsive transcription factor (ETRTF)	HRO_ETRTF_5a	PGSC0003 DMG400002185	PGSC0003 DMT400005585	PGSC0003 DMT400005585	11:18921453-18922659	1207	chr11:18766243..18767449 forward	1207	1207	873	11	$(ACC)_4$ $(AAC)_3$	Compound	Exon 1	CACGTTCCATGCGTGTTAA<br>AATCGACGTTAGGAGCAGGA	55	36	198
74	Ethylene responsive transcription factor 3 (ETRTF3)	HRO_ETRTF3_2	PGSC0003 DMG400014417	PGSC0003 DMT400037359	PGSC0003 DMT400037359	10:1121218-1122800	1583	chr10:314882..316464 forward	1583	672	672	10	$(CGG)_7$	Pure	Exon 1	GGTCATGAGGAGAGGCAGAG<br>CCAAACCCTAGTTTTCTTCCA	55	36	169
75	Ethylene responsive transcription factor 3 (ETRTF3)	HRO_ETRTF3_3	PGSC0003 DMG400022305	PGSC0003 DMT400057443	PGSC0003 DMT400057443	7:55079184-55080640	1457	chr07:53080191..53081647 reverse	1457	723	723	7	$(GCC)_3$ CCC $(GCA)_2$	Compound	Exon 1	CAGGACCACCCGATAGTTTC<br>TCACAGTCGCTATGGCAATC	nd	nd	173
76	Ethylene responsive transcription factor 7 (ETRTF7)	HRO_ETRTF7_1	PGSC0003 DMG401013892	PGSC0003 DMT400036071	PGSC0003 DMT400036071	12:6424671-6425243	573	chr12:6336051..6336623 forward	573	573	573	12	$(TCG)_2$ $(TCT)_2$ $(GTG)_2$	Complex	Exon 1	AAATTCCTCGCCGGATTC<br>AGAAACACAAAGGGGCAATG	nd	nd	189
77	Ethylene responsive transcription factor 13 (ETRTF13)	HRO_ETRTF13_1A	PGSC0003 DMG400026046	PGSC0003 DMT400067013	PGSC0003 DMT400067013	1:69601395-69602081	687	chr01:75741462..75742148 forward	687 (without UTR)	687	687	1	$(CTT)_3$ $(CTC)_5$	Compound	Intron 1	TAGAGTGAGGCCGAGGAGAC<br>GACCATCCAAATTGTTTACCG	55	31	185
78	Extensin	HRO_EXT_1A	PGSC0003 DMG401032203	PGSC003 DMT40082012	PGSC0003 DMT400082014	1:1940714-1946323	5610	chr01:1444249..1449858 reverse	5610	5461	726	1	$(TA)_6$	Pure	Intron 2	GAAAATTTATCGACTATCCAAATAATG<br>TCAATTGAATGATTGTTATCGATTT	nd	nd	121
79	Extensin	HRO_EXT_1B	PGSC0003 DMG401032203	PGSC003 DMT40082012	PGSC0003 DMT400082014	1:1940714-1946323	5610	chr01:1444249..1449858 reverse	5610	5461	726	1	$(CA)_2$ $(TA)_2$ $(GA)_3$	Complex	Intron 2	TTGAGAAGTATATTTATTCATTTTT<br>CATGTGTATCATGTGATAGTGAAAAG	nd	nd	102
80	Extensin	HRO_EXT_1C	PGSC0003 DMG401032203	PGSC003 DMT40082012	PGSC0003 DMT400082014	1:1940714-1946323	5610	chr01:1444249..1449858 reverse	5610	5461	726	1	$(GGT)_2$ GGAG $(GTG)_3$ ATC $(GTG)_3$ GA $(GGT)_2$	Interrupted complex	Exon 1	TGCATTTCTTCACCTTTGCTT<br>CTCCAAAACCACGACCATGT	nd	nd	135
81	Extensin	HRO_EXT_1D	PGSC0003 DMG401032203	PGSC0003 DMT400082012	PGSC0003 DMT400082012	1:1940714-1946323	5610	chr01:1444249..1449858 reverse	5610	5461	726	1	$(ACC)_2$ TC $(CAC)_3$ GAT $(CAC)_3$ CT $(CCA)_3$	Interrupted complex	Exon 1	ACAATTATGGCCATGGGAAA<br>CACCTTTGCTTTGTCACCTG	55	36	199
82	Flagellin-sensing 2 (FLS2)	HRO_FLS2_1A	PGSC0003 DMG401026747	PGSC0003 DMT400068776	PGSC0003 DMT400068776	3:1303348-1307104	3757	chr03:13924..17680 reverse	3757	2843	1146	3	$(AT)_3$ $(TAT)_2$ $(GA)_2$	Complex	3'-UTR	CCCCGAGGGTATTTCACTGT<br>GAGCAACATGGTAGGTTTGGA	55	36	142
83	Flagellin-sensing 2 (FLS2)	HRO_FLS2_1B	PGSC0003 DMG401026747	PGSC0003 DMT400068776	PGSC0003 DMT400068776	3:1303348-1307104	3757	chr03:13924..17680 reverse	3757	2843	1146	3	$(AT)_2$ G $(GAA)_2$ AA $(AT)_3$	Interrupted complex	Intron 1	GCGTTATGGGAATAGGGATCT<br>CCCTGATTTTCTTACCTTATTTGAA	nd	nd	178

84	Gibberellin 3-oxidase (Ga3ox)	HRO_G3O_1	PGSC0003 DMG400016516	PGSC0003 DMT400042582	PGSC0003 DMT400042582	6:49523127-49525846	2720	chr06:43045522..43048241 forward	2720	2552	1119	6	$(ACC)_5 (AAT)_3$	Compound	Exon 1	CATGCATGGACATCATCAAA TGAAATGCACCCCATTTCTT	55	36	152
85	Glucosyltransferase (GT)	HRO_GLUCT_7	PGSC0003 DMG400008138	PGSC0003 DMT400021019	PGSC0003 DMT400021019	10:58444348-58445769	1422	chr10:44471577..44472998 reverse	1422 (without UTR)	1422	1422	10	$(AC)_2 (AT)_3$ TACCGA $(GTTT)_2$	Interrupted complex	Exon 1	TCTCTGAGGACCCAAATGAA CACCATCAAACCCTGTATTGG	nd	nd	155
86	Glucosyltransferase (GT)	HRO_GLUCT_7B	PGSC0003 DMG400008138	PGSC0003 DMT400021019	PGSC0003 DMT400021019	10:58444348-58445769	1422	chr10:44471577..44472998 reverse	1422 (without UTR)	1422	1422	10	$(TGA)_2$ GCCTC $(GA)_2$ GGTG $(GA)_2$	Interrupted complex	Exon 1	AGCTGTGACAGGATCGAAGC TGGTTATGGTGCCACTTTCA	nd	nd	158
87	Glucosyltransferase (GT)	HRO_GLUCT_7C	PGSC0003 DMG400008138	PGSC0003 DMT400021019	PGSC0003 DMT400021019	10:58444348-58445769	1422	chr10:44471577..44472998 reverse	1422 (without UTR)	1422	1422	10	$(TC)_7$ TGTT $(TC)_3$	Interrupted pure	Intergenic region	TGTTCCACCAAAATCATAGCTT CCGGCCATGTTGTTGTAGTT	55	36	214
88	Glutathione reductase (GR)	HRO_GR_1	PGSC0003 DMG400007537	PGSC0003 DMT400019491	PGSC0003 DMT400019491	9:48830689-48837646	6958	chr09:41364863..41371820 reverse	6958	4677	1671	9	$(CA)_2 (TG)_3 (GA)_2$	Complex	Exon 8	AAGGCTACTCTTTCTGGTCTGC TCAATCTGAAATATCCACCAAA	nd	nd	159
89	Glutathione reductase (GR)	HRO_GR_2	PGSC0003 DMG400007537	PGSC0003 DMT400019491	PGSC0003 DMT400019491	9:48830689-48837646	6958	chr09:41364863..41371820 reverse	6958	4677	1671	9	$(TTCC)_2$ T $(CTT)_3$	Interrupted compound	Intron 9	TCAGTGAAACTGAAGTCTGAAACC TTGAAGTTTCCACAGAGTGTGA	nd	nd	196
90	Glutathione-S-Transferase (GST)	HRO_GLUTST_1A	PGSC0003 DMG400001804	PGSC0003 DMT400004539	PGSC0003 DMT400004541	1:67558231-67567291	9061	chr01:73745245..73752358 reverse	7114	6481	675	1	$(AT)_4$ G $(TA)_2$	Interrupted compound	Intron 1	TAGGTGGGGTTCGTGAGTTT ATTTTCATGGCCCTTTCCTT	55	36	153
91	Glutathione-S-Transferase (GST)	HRO_GLUTST_1B	PGSC0003 DMG400001804	PGSC0003 DMT400004539	PGSC0003 DMT400004541	1:67558231-67567291	9061	chr01:73745245..73752358 reverse	7114	6481	675	1	$(ACA)_2 (GT)_4$	Compound	Intron 1	AAGCTAAAGTTGATTTGTAGTTTGGA TGAAAACAAGAGCAAAGACCAA	nd	nd	125
92	Glutathione-S-Transferase (GST)	HRO_GLUTST_1C	PGSC0003 DMG400001804	PGSC0003 DMT400004539	PGSC0003 DMT400004541	1:67558231-67567291	9061	chr01:73745245..73752358 reverse	7114	6481	675	1	$(GT)_4$ AT $(TAG)_2$	Interrupted compound	Intron 1	GACATCATTTAATAGGGAATAATTTGA CATGGCAAGAAAAAGTGTACCA	55	36	131
93	Jasmonic acid 2 (JA2)	HRO_JA2_1	PGSC0003 DMG400015342	PGSC0003 DMT400039670	PGSC0003 DMT400039670	12:822922-825609	2688	chr12:822922..825609 forward	2688	2209	1056	12	$(TA)_2$GT $(TG)_2 (TAA)_3$AA $(TGA)_2$	Interrupted complex	Intron 1	TGGATGGATTCATAATTAAAGTCA GCTTAGCATCCGAGCAACTC	55	36	223
94	JERF1 Transcription factor	HRO_JERF1_1	PGSC0003 DMG400004898	PGSC0003 DMT400012547	PGSC0003 DMT400012547	6:48380518-48383025	2508	chr06:41800450..41802957 reverse	2508	2147	1119	6	$(CT)_2$ GG $(TTC)_2 (GAT)_2$	Interrupted complex	Exon 2	GGCTTTTGAATCCCAGATGA GCAAGGGAAGACGTTTATGC	nd	nd	189
95	Kinase (KIN)	HRO_KIN_1	PGSC0003 DMG400016429	PGSC0003 DMT400042362	PGSC0003 DMT400042362	1:3617273-3617745	473	chr01:3120808..3121280 forward	473	180	180	1	$(TC)_2$ AAGC $(AT)_4$	Interrupted compound	3'-UTR	CACAGTTCTTTTTACCCCAGATG GCTCGATAACGATGAAGTCTCC	55	36	132
96	Kinase (KIN)	HRO_KIN_1B	PGSC0003 DMG400016429	PGSC0003 DMT400042362	PGSC0003 DMT400042362	1:3617273-3617745	473	chr01:3120808..3121280 forward	473	180	180	1	$(AAGA)_2$ TGA $(AG)_2$ TTG $(CCT)_3$	Interrupted complex	Exon 1	GATCCCAACGTTACGTCCAT GTTGGCAATCGAAGCAGAAT	nd	nd	172
97	Late embryogenesis abundant protein 5 (LEA5)	HRO_LEA5_1	PGSC0003 DMG400017936	PGSC0003 DMT400046207	PGSC0003 DMT400046207	6:461908-463086	1179	chr06:3213972..3215150 forward	1179	629	270	6	$(TA)_3$ A $(TA)_2$ A $(TA)_2$	Interrupted pure	5'-UTR	AAATACCTCTCTTCCTCACACTATTTT AATTACTTCCGCAGCAACAAA	54	36	142
98	Lipoxygenase (LOX)	HRO_LIPOX_1	PGSC0003 DMG400032207	PGSC0003 DMT400082023	PGSC0003 DMT400082023	1:2151329-2152141	813	chr01:1654864..1655676 reverse	813	453	453	1	$(TA)_4 (CTT)_2$	Compound	3-UTR	TTGAGACCAAGGCTCAAAGC TTATAGCATTTCCATTTGATTTTT	nd	nd	155
99	Lipoxygenase (LOX)	HRO_LIPOX_1B	PGSC0003 DMG400032207	PGSC0003 DMT400082023	PGSC0003 DMT400082023	1:2151329-2152141	813	chr01:1654864..1655676 reverse	813	453	453	1	$(TC)_3$ TA $(CT)_2$	Interrupted compound	5'-UTR	TTGTTGGCCTATTTGGAAAG CAAGTGAGGTCTGAGAGGGTAGA	60	32	135
100	LRR receptor-like serine/threonine-protein kinase (LRR-STPK)	HRO_LRRSTPK_1A	PGSC0003 DMG400027968	PGSC0003 DMT400071885	PGSC0003 DMT400071885	4:63318594-63322913	4320	chr04:55449855..55454174 forward	4320 (without UTR)	4320	3852	4	$(TG)_3$ A $(TG)_2 (TA)_2$	Interrupted complex	Exon 4	GCCAAACACTCCATTGTTTTTA ATTCCCCAAGATGCTTTCCT	nd	nd	171
101	LRR receptor-like serine/threonine-protein kinase (LRR-STPK)	HRO_LRRSTPK_1B	PGSC0003 DMG400027968	PGSC0003 DMT400071885	PGSC0003 DMT400071885	4:63318594-63322913	4320	chr04:55449855..55454174 forward	4320 (without UTR)	4320	3852	4	$(AAG)_2$ TTGA $(AAG)_2$ G $(GATGGT)_2$	Interrupted complex	Exon 2	TGCTCTGTGTTTTCATTGGTG TCTGTGGCGTTTAAGATGTCC	nd	nd	145
102	LRR receptor-like serine/threonine-protein kinase (LRR-STPK)	HRO_LRRSTPK_1C	PGSC0003 DMG400027968	PGSC0003 DMT400071885	PGSC0003 DMT400071885	4:63318594-63322913	4320	chr04:55449855..55454174 forward	4320 (without UTR)	4320	3852	4	$(AT)_3 (CATAT)_2$ A $(ATATC)_2 (AT)_3$	Interrupted complex	Intergenic region	TCCCGTCTCAATGAAGAACA AAAATGGTAATCTATCTTTTTCCTAA	57	36	203
103	Molybdenum Cofactor sulfurase (MOCOS)	HRO_MOCOS_1A	PGSC0003 DMG400022125	PGSC0003 DMT400056893	PGSC0003 DMT400056893	7:56514925-56527675	12751	chr07:51633156..51645906 forward	12751	9838	1821	7	$(TCT)_3$ G $(CA)_2$ ATTT $(AT)_2$ TGGT $(TG)_2$	Interrupted complex	5'-UTR	GCGACCCCTTTGATTCCTTA CAGAGCAAAGGCAGAGGAAG	55/57	36	184
104	Methionyl-tRNA synthetase (MetRS)	HRO_MTRNAS_1B	PGSC0003 DMG400019113	PGSC0003 DMT400049172	PGSC0003 DMT400049172	5:48910277-48917779	7503	chr05:58171816..58179318 forward	7503	4229	735	5	$(CT)_3 (TC)_2$ CCTG $(CT)_3$	Interrupted complex	Intron 3	TCTCTTTTCTCCTTCTTCTGTTCC TCCACGCAGAAGAGGTACTG	nd	nd	104
105	Methionyl-tRNA synthetase (MetRS)	HRO_MTRNAS_1D	PGSC0003 DMG400019113	PGSC0003 DMT400049172	PGSC0003 DMT400049172	5:48910277-48917779	7503	chr05:58171816..58179318 forward	7503	4229	735	5	$(AT)_3$ AC $(AT)_3 (GT)_3$	Interrupted complex	Intron 5	CGACTTTTGCAAGGCCAGTA TTGGTCTAGCCACCTAATGTGA	57	36	131
106	Multidrug resistance protein ABC transporter family (MRP)	HRO_MRPATF_3A	PGSC0003 DMG400012167	PGSC0003 DMT400031714	PGSC0003 DMT400031715	8:55531912-55539184	7273	chr08:42641417..42648689 forward	7273	3679	3249	8	$(GT)_2$ TTTG $(TA)_3$ CATT $(TGA)_2$	Interrupted complex	5-UTR	CAGGATTTGAAGTTTATGAGTTTGG CTTCCCTTACGGATTCAGCA	nd	nd	149
107	Multidrug resistance protein ABC transporter family (MRP)	HRO_MRPATF_3B	PGSC0003 DMG400012167	PGSC0003 DMT400031714	PGSC0003 DMT400031715	8:55531912-55539184	7273	chr08:42641417..42648689 forward	7273	3679	3249	8	$(CA)_2 (CCT)_2 (AT)_3$	Complex	Exon 4	ATATCTTGGCGAAAGCAACG GGATGAGATTCAATTTGCTGGT	nd	nd	143
108	Multidrug resistance protein ABC transporter family (MRP)	HRO_MRPATF_3D	PGSC0003 DMG400012167	PGSC0003 DMT400031714	PGSC0003 DMT400031714	8:55531912-55539184	7273	chr08:42641417..42648689 forward	7273	3679	3249	8	$(ATA)_2$ TA $(AT)_6 (GAT)_2$	Interrupted complex	Intergenic region	TTTGACATAGAGGCTTCCCACT CCATTCTTGTTTGTTGAATACCA	55	32	163
109	No apical meristem (NAM) (-->RD 26, similar to NAM family protein)	HRO_NAM	PGSC0003 DMG400031266	PGSC0003 DMT400080307	PGSC0003 DMT400080307	5:4946134-4948792	2659	chr05:4687504..4690162 reverse	2659	2548	1164	5	$(TA)_2 (TAT)_2$ TT $(TAT)_3$ TA $(GT)_2$	Interrupted complex	Intron 2	CTTATTTTGTGGATCTTCCCATA TGTGCATTGGAGTTTGCTTT	nd	nd	166
110	9-cis-Epoxy-carotenoid dioxygenase (NCED)	HRO_NCED_1	PGSC0003 DMG400027633	PGSC0003 DMT400071048	PGSC0003 DMT400071048	7:51778895-51781175	2281	chr07:50086448..50088728 forward	2281	1521	1521	7	$(TAC)_5$	Pure	5'-UTR	TCACCTCAAAAGGAAAACACAA GATGGCATTGACAACTTAGGC	nd	nd	203
111	9-cis-Epoxycarotenoid dioxygenase (NCED)	HRO_NCED_2	PGSC0003 DMG400027633	PGSC0003 DMT400071048	PGSC0003 DMT400071048	7:51778895-51781175	2281	chr07:50086448..50088728 forward	2281	1521	1521	7	$(GA)_3$G $(TA)_4$	Interrupted compound	3'-UTR	AGCTCAGCTCGTTTTTCTGG GGGTGGAAATAGTATAAGCCATGA	57	36	145
112	9-cis-Epoxycarotenoid dioxygenase (NCED)	HRO_NCED_3	PGSC0003 DMG400004311	PGSC0003 DMT400011006	PGSC0003 DMT400011006	8:39185413-39188702	3290	chr08:28315023..28318312 forward	3290 (without UTR)	3290	1824	8	$(TA)_3 (TGTTT)_2 (CTT)_3$	Complex	Intron 6	TTGAACTTGGTGTCATTTCAGG TTAAATTGAGTAACGTTGTGAAAAA	55	36	134
113	Nucleoside diphosphate kinase 2 (NDPK)	HRO_NDPK2_1	PGSC0003 DMG400026976	PGSC0003 DMT400069352	PGSC0003 DMT400069352	6:52807449-52811263	3815	chr06:48478602..48482416 forward	3815	3307	675	6	$(AT)_4 (TTA)_2$	Complex	Intron 5	AGGGTGTTGGTGTTGTTGCT TTGCCATCAGAATCCATGAC	57	36	182
114	Neutral invertase (NI)	HRO_NE1_1	PGSC0003 DMG400013088	PGSC0003 DMT400034052	PGSC0003 DMT400034052	1:37067233-37072498	5266	chr01:18007260..18012525 reverse	5266	4689	2037	1	$(GT)_3$ A $(TG)_3$	Interrupted compound	Intron 1	CCAATTGCTGTCAATGATGC AAAGCTGCAATCCTTCATGG	nd	nd	143
115	Neutral invertase (NI)	HRO_NE11_3	PGSC0003 DMG400026530	PGSC0003 DMT400068246	PGSC0003 DMT400068246	11:39907597-39913829	6233	chr11:37090122..37096352 forward	6231	4767	1926	11	$(AC)_5 (AT)_2$	Compound	Intron 5	GCTTTCGGCACGTATAGCTT TGCATATTTATACACATACGAAAAACA	nd	nd	169
116	Poly (ADP-ribose) glycohydrolase (PARG)	**HRO_PARGH_1A**	PGSC0003 DMG400029361	PGSC0003 DMT400075512	PGSC0003 DMT400075512	12:58212212-58218855	6644	chr12:64844605..64851248 forward	6644	6078	1692	12	$(GA)_4$ AA $(GA)_2$	Interrupted pure	Intron 9	GCCTAAAAACTACGATGCGACT CGGGAAGTATTGGCTCATGT	55	36	135
117	Poly (ADP-ribose) glycohydrolase (PARG)	HRO_PARGH_1C	PGSC0003 DMG400029361	PGSC0003 DMT400075512	PGSC0003 DMT400075512	12:58212212-58218855	6644	chr12:64844605..64851248 forward	6644	6078	1692	12	$(TA)_2$ G $(TA)_8$	Interrupted pure	Intron 2	ATATTGGCCACAACCACACC AGAGCCTGGCATTGTCTTTC	56	34	175
118	Proline oxidase/ dehydrogenase 1 (PRODH, PDH)	HRO_POD1_2	PGSC0003 DMG400010050	PGSC0003 DMT400026049	PGSC0003 DMT400026049	2:45033737-45039948	6212	chr02:66292969..66295391 forward	2423	1813	1503	2	$(TG)_3 (AT)_4$	Compound	Intron 2	AAATAAATTTGTTCGCTATTGTCTAA CAAATTGGAGATTTTGCCTCTC	55	36	117
119	Protein phosphatase 2c (PP2C)	**HRO_PP1_1 (HRO_PP2C)**	PGSC0003 DMG400011321	PGSC0003 DMT400029441	PGSC0003 DMT400029441	1:58016454-58020478	4025	chr01:64271792..64275816 forward	4025	2194	1566	1	$(GAA)_3$ A $(AT)_2$ CAT $(GAT)_2$	Interrupted complex	Exon 3	CCTATTTGCATTTCTTGATGAGC GATGCTTTTGAGGTGCGTTT	55	36	182
120	Protein phosphatase 2c (PP2C)	HRO_PP1_2 (HRO_PP2C1_2)	PGSC0003 DMG400032110	PGSC0003 DMT400081763	PGSC0003 DMT400081763	1:60945180-60952491	7312	chr01:67200518..67207829 reverse	7312	6226	1245	1	$(GGT)_2 (GT)_2 (CT)_3$	Complex	Intron 1	CACCCAAAGTTGGAGAGCAT TCCAATATCTTGCTGTAACAGAGC	55	36	149
121	Protein phosphatase 2c (PP2C)	HRO_PP1_3 (HRO_PP2C1_3)	PGSC0003 DMG400032110	PGSC0003 DMT400081763	PGSC0003 DMT400081763	1:60945180-60952491	7312	chr01:67200518..67207829 reverse	7312	6226	1245	1	$(GT)_2$ CAG $(TA)_2$ T $(GT)_4$	Interrupted complex	Intron 1	CTGGTGGTGTGTCTCTCTGC GCATAGTCTCCAATAGGGAAAA	nd	nd	200
122	Protein phosphatase 2c (PP2C)	HRO_PP2_1	PGSC0003 DMG400001510	PGSC0003 DMT400003812	PGSC0003 DMT400003812	2:38333034-38336937	3904	chr02:60384102..60388005 forward	3904	3000	1155	2	$(TA)_7$ T $(TC)_2$	Interrupted compound	Intron 2	ACATATGGTTCCGCCTTCAC TTTTGGCAACGTAACAAATCC	nd	nd	186
123	Pyrroline-5-carboxylate reductase (P5CR)	HRO_P5CR_2	PGSC0003 DMG400010441	PGSC0003 DMT400027070	PGSC0003 DMT400027070	2:27511074-27519810	8737	chr02:49037101..49045837 forward	8737	7933	825	2	$(TA)_3 (TAAAA)_2$	Compound	Intron 3	TGGATTTCTGCTCATTTTATGTG TTCTCACCTAATTTTGTGTGTGATT	55	36	184
124	Receptor protein kinase (RPK)	HRO_RPK_10A	PGSC0003 DMG400017718	PGSC0003 DMT400045689	PGSC0003 DMT400045691	4:63083149-63086451	3303	chr04:54068506..54070997 reverse	2492	1138	1035	4	$(TA)_2 (CA)_2$ T $(CA)_3$	Interrupted complex	Exon 2	TTCATCCAGCAAATCGCTAA TGAAAATTGTATTATTCAGAGCTTGC	nd	nd	180
125	Receptor protein kinase (RPK)	HRO_RPK_10B	PGSC0003 DMG400017718	PGSC0003 DMT400045689	PGSC0003 DMT400045691	4:63083149-63086451	3303	chr04:54068506..54070997 reverse	2492	1138	1035	4	$(CAT)_3$ CC $(CTT)_2$	Interrupted compound	Exon 1	GTCCCTGTACAATGCCTTCC TGTACTTCCTGTTATGGGAGCA	nd	nd	157
126	Receptor protein kinase (RPK)	HRO_RPK_10C	PGSC0003 DMG400017718	PGSC0003 DMT400045689	PGSC0003 DMT400045691	4:63083149-63086451	3303	chr04:54068506..54070997 reverse	2492	1138	1035	4	$(AAG)_2$ G $(GAT)_3$	Interrupted compound	Exon 1	TGCTCTGTGTTTTCATTGGTG GTAAACGCTTCCGTGTCCTC	nd	nd	197

127	Receptor protein kinase (RPK)	HRO_RPK_10D	PGSC0003<br>DMG400017718	PGSC0003<br>DMT400045689	PGSC0003<br>DMT400045689	4:63083149-63086451	3303	chr04:54069856..54071808 reverse	1953 (without UTR)	1953	1953	4	$(TC)_2$ CCA $(AT)_3$ TTC $(CA)_2$	Interrupted complex	Intergenic region	AGTTGCGCGATTTCTTCCTA<br>CATGGAAGTCAATGTGTGAGTG	55	36	158
128	Receptor protein kinase (RPK)	HRO_RPK_10E	PGSC0003<br>DMG400017718	PGSC0003<br>DMT400045689	PGSC0003<br>DMT400045691	4:63083149-63086451	3303	chr04:54068506..54070997 reverse	2492	1138	1035	4	$(TG)_3$ A $(TG)_2$ $(TA)_2$	Interrupted complex	Exon 2	GGCTATGTCGCACCTGGTAA<br>CCCAAGATGCTTTCCTTTGA	nd	nd	211
129	SBT4B protein	HRO_SBT4B_1	PGSC0003<br>DMG400006840	PGSC0003<br>DMT400017633	PGSC0003<br>DMT400017634	1:65018707-65021313	2607	chr01:71203774..71206380 reverse	2607	1353	1353	1	$(TG)_2$ GCC $(TGC)_2$ $(CA)_4$	Interrupted complex	Exon 1	ACAAAGGCTCTTGCAGGAAA<br>GAAATCGAGGTCCAGGGATT	nd	nd	157
130	SBT4B protein	HRO_SBT4B_1B	PGSC0003<br>DMG400006840	PGSC0003<br>DMT400017633	PGSC0003<br>DMT400017634	1:65018707-65021313	2607	chr01:71203774..71206380 reverse	2607	1353	1353	1	$(AG)_2$ G $(TAT)_2$ T $(GTA)_3$	Interrupted complex	5'-UTR	AAAGCATGAGGGTCCCTTG<br>TTTTATACAAGCCGCCTACCTT	55	36	101
131	SCARECROW (SCR)	HRO_SCRCRW	PGSC0003<br>DMG400016833	PGSC0003<br>DMT400043358	PGSC0003<br>DMT400043358	10:48250273-48254623	4351	chr10:39916234..39920584 reverse	4351	3866	2481	10	$(CCT)_2$ $(GCT)_2$ $(GCA)_2$	Complex	Exon 1	CAATCCCCATCTGTTTGTG<br>TGCTATCCTCCATGGAATCG	nd	nd	160
132	Serine-threonine protein kinase (STPK)	HRO_STPK	PGSC0003<br>DMG400000652	PGSC0003<br>DMT40001743	PGSC0003<br>DMT40001743	12:42294180-42298253	4074	chr01:71075826..71081106 forward	5281 (without UTR)	5281	2517	12	$(CT)_3$ TTT $(CT)_2$ TT $(CT)_3$	Interrupted pure	5'-UTR	CAAATCTACGCCAAAAGATTAAA<br>TCAGCTCCTTCTCGATTGTG	57	36	178
133	Serine-threonine protein kinase (STPK), plant-type	HRO_STPKPT_1A	PGSC0003<br>DMG400010951	PGSC0003<br>DMT400028434	PGSC0003<br>DMT400028434	00:31420301-31421237	937	chr03:3517472..3518408 reverse	937	282	282	0 (3?)	$(TG)_2$ $(TA)_2$ AATCA $(CT)_2$	Interrupted complex	5'-UTR	AGAGACGTACCCGATGCAAT<br>ATATGGTTGCCCACCTAAGC	55	36	115
134	Serine-threonine protein kinase (STPK), plant-type	HRO_STPKPT_1B	PGSC0003<br>DMG400010951	PGSC0003<br>DMT400028434	PGSC0003<br>DMT400028434	00:31420301-31421237	937	chr03:3517472..3518408 reverse	937	282	282	0 (3?)	$(AGT)_2$ $(TG)_3$	Compound	5'-UTR	TCCAACGAACATGCCACAT<br>GGAATCTCGTGAAAGTCATCA	55	36	166
135	S-locus-specific glycoprotein S6 (SLSG6)	HRO_SLSGPS6_5A	PGSC0003<br>DMG401013876	PGSC0003<br>DMT400036026	PGSC0003<br>DMT400036023	2:35398866-35403067	4202	chr02:56654435..56658437 reverse	4003	3337	2433	2	$(AT)_4$ T $(CA)_2$	Interrupted compound	Exon 1	CATTGCATGAGCAATTGGAT<br>TGCAGTGTGGCAGCAATAAC	nd	nd	132
136	S-locus-specific glycoprotein S6 (SLSG6)	HRO_SLSGPS6_5B	PGSC0003<br>DMG401013876	PGSC0003<br>DMT400036026	PGSC0003<br>DMT400036023	2:35398866-35403067	4202	chr02:56654435..56658437 reverse	4003	3337	2433	2	$(CA)_3$ C $(CCA)_2$ G $(AAT)_2$	Interrupted complex	Exon 1	TGCACTGATTCAGATGATGC<br>CCAGTCCAATCTGTGGATGTT	nd	nd	175
137	S-locus-specific glycoprotein S6 (SLSG6)	HRO_SLSGPS6_5C	PGSC0003<br>DMG401013876	PGSC0003<br>DMT400036026	PGSC0003<br>DMT400036023	2:35398866-35403067	4202	chr02:56654435..56658437 reverse	4003	3337	2433	2	$(ATA)_2$ $(AAT)_4$ ACCT $(TA)_2$	Interrupted complex	Intergenic region	CAACGTTCACACCGAAAAAG<br>GCAAAGTTCGACCACCCTAC	55	36	183
138	Snakin-1 (SN-1)	HRO_SN1_1	PGSC0003<br>DMG400014441	PGSC0003<br>DMT400037423	PGSC0003<br>DMT400037423	12:49371055-49372039	985	chr12:55666169..55667153 reverse	985	801	267	12	$(AT)_4$ AA<br>$(AT)_2$	Interrupted compound	Intron 1	CAGGTCGGAGCTAAAAGTTCA<br>TGGCTAGTAAATGTAAAGGTGGA	nd	nd	200
139	Soluable anorganic pyrophosphatase (siPPiase)	HRO_SIPP_1	PGSC0003<br>DMG400012223	PGSC0003<br>DMT400031867	PGSC0003<br>DMT400031867	8:56807414-56809327	1914	chr08:41371274..41373187 reverse	1914	1313	639	8	$(ATC)_2$ AC $(AT)_5$	Interrupted compound	5'-UTR	GGAAAAGGAATCGGACAACA<br>GGCGTGTCTTTGTTCATCAC	55	36	206
140	Superoxide dismutase (SOD)	HRO_SOD_1	PGSC0003<br>DMG400005247	PGSC0003<br>DMT400013447	PGSC0003<br>DMT400013447	3:36657156-36660903	3748	chr03:24127144..24130891 reverse	3748	2672	912	3	$(GAA)_2$ $(GA)_3$ $(GGA)_2$ A<br>$(GAG)_2$	Interrupted complex	Exon 9	TTGAAGCAGCTCAAGCTCAA<br>TCTGCTTTCGCAAAATTGAC	nd	nd	200
141	Superoxide dismutase (SOD)	HRO_SOD_2	PGSC0003<br>DMG400027577	PGSC003<br>DMT400070918	PGSC0003<br>DMT400070920	6:35297776-35306389	8614	chr06:31466066..31474679 reverse	8614	7831	756	6	$(TA)_2$ $(TTTA)_2$TG $(AT)_3$	Interrupted complex	Intron 8	CGATTTTGAGTTTATGTGTTCTGA<br>CCAAAAAATTGATCTCAAATGGA	nd	nd	196
142	TIR-NBS-LRR disease resistance (TNL-DR)	HRO_TNLDR_2A	PGSC0003<br>DMG400027797	PGSC0003<br>DMT400071445	PGSC0003<br>DMT400071445	11:9730445-9736116	5672	chr11:9172641..9178312 reverse	5672 (without UTR)	5672	3195	11	$(AT)_4$ AAA $(TGA)_2$	Interrupted compound	Intron 2	TGCAGAGCTTCTAAAATACTATAATTG<br>CAAACATCCCCTCTCTGCAT	50	36	164
143	TIR-NBS-LRR disease resistance (TNL-DR)	HRO_TNLDR_2B	PGSC0003<br>DMG400027797	PGSC0003<br>DMT400071445	PGSC0003<br>DMT400071445	11:9730445-9736116	5672	chr11:9172641..9178312 reverse	5672 (without UTR)	5672	3195	11	$(GTA)_5$ AAAC $(TA)_2$	Interrupted compound	Intron 1	TTTACGCAATACCTCCTGGAT<br>GGAAAATTGGAAAACATTTTCTTG	nd	nd	
144	TIR-NBS-LRR disease resistance (TNL-DR)	HRO_TNLDR_2C	PGSC0003<br>DMG400027797	PGSC0003<br>DMT400071445	PGSC0003<br>DMT400071445	11:9730445-9736116	5672	chr11:9172641..9178312 reverse	5672 (without UTR)	5672	3195	11	$(CAT)_3$ $(CT)_2$ AA $(AC)_2$	Interrupted complex	Exon 1	GTGAAATGTCCTCGCCTCTT<br>GAATTTTAGGGGAAAAGATGTGC	nd	nd	122
145	Trehalose-6-phosphate synthase 1 (TPS 1)	HRO_TPS1_1	PGSC0003<br>DMG400007089	PGSC0003<br>DMT400018267	PGSC0003<br>DMT400018267	7:52282758-52284752	1995	chr07:49582871..49584865 forward	1995	1418	804	7	$(TA)_3$ $(CA)_3$	Compound	Intron 3	CCGAGCAGTTGGTGTTACAA<br>TTGGTGTTGCAATGGCTTTA	60	36	156
146	UDP-glucose:glucosyltransferase (UGGT)	HRO_UDPGGT_1A	PGSC0003<br>DMG400019093	PGSC0003<br>DMT400049125	PGSC0003<br>DMT400049125	00:38379277-38379630	354	-	-	-	-	0	$(TG)_2$ ATT $(GA)_2$ T<br>$(TGGG)_2$	Interrupted complex	unknown	TTGGACATTTTCAGGGCTTT<br>CCCGAAAAGTATGCTGATTTG	55	36	141
147	UDP-glucose:glucosyltransferase (UGGT)	HRO_UDPGGT_1B	PGSC0003<br>DMG400019093<br>(SPUD DB)	PGSC0003<br>DMT400049125<br>(SPUD DB)	-	00:38379277-38379630	354	-	-	-	-	0	$(GA)_3$ $(TG)_2$	Compound	unknown	TTTCTTGATGGTGGATTTGAGA<br>TTCCCATTTTTGGCTTATGG	nd	nd	123
148	UTP-glucose-1-phosphate uridylyltransferase (UGPase)	HRO_UGP_1	PGSC0003<br>DMG401013333	PGSC0003<br>DMT400034699	PGSC0003<br>DMT400034699	11:808268-814810	6543	chr11:2402051..2408593 forward	6543	6248	1434	11	$(TA)_2$ $(GT)_8$	Compound	Intron 2	GTGTGTGCGTGCAAAATGTA<br>TGATCTGCAAAAGCTCGGATA	57	36	177
150	Vacuolar protein sorting protein (VPSP)	HRO_VPSP_1A	PGSC0003<br>DMG400003501<br>(SPUD DB)	PGSC0003<br>DMT400008982<br>(SPUD DB)	-	00:36520614-36528235	7622	-	-	-	-	0	$(AT)_3$ GCT $(TC)_2$ $(TG)_2$	Interrupted complex	unknown	TCCCTTTTTGTGGAAAGCAT<br>AAAAATCCAGAATTCGAAGTGAA	nd	nd	151
149	Vacuolar protein sorting protein (VPSP)	HRO_VPSP_1B	PGSC0003<br>DMG400003501<br>(SPUD DB)	PGSC0003<br>DMT400008982<br>(SPUD DB)	-	00:36520614-36528235	7622	-	-	-	-	0	$(TGG)_2$ ACT $(AT)_2$ G $(GA)_4$	Interrupted complex	unknown	TTTCCTCCTTCGAATTTACTGA<br>CTTCCTGCAGGTTGTTGTCC	60	32	157
151	Vacuolar protein sorting protein (VPSP)	HRO_VPSP_1C	PGSC0003<br>DMG400003501<br>(SPUD DB)	PGSC0003<br>DMT400008982<br>(SPUD DB)	-	00:36520614-36528235	7622	-	-	-	-	0	$(GCT)_2$ T $(TA)_4$	Interrupted compound	unknown	TCCATTGTTCAGTAATGCTGCT<br>TCTGGCATTCAAGATCACTAGC	nd	nd	177
152	Vacuolar protein sorting protein (VPSP)	HRO_VPSP_1D	PGSC0003<br>DMG400003501<br>(SPUD DB)	PGSC0003<br>DMT400008982<br>(SPUD DB)	-	00:36520614-36528235	7622	-	-	-	-	0	$(TC)_3$ AC $(TG)_3$ A $(CAA)_5$	Interrupted complex	unknown	TTCCAGGTAGAATGAACATTGA<br>ACCCCACCTAGTGGGATTTC	nd	nd	159
153	WRKY transcription factor (WRKY TF)	HRO_WRKYTF_1B	PGSC0003<br>DMG400008188	PGSC0003<br>DMT400021144	PGSC0003<br>DMT400021144	8:34572458-34573154	697	chr08:19161502..19162198 reverse	697	523	387	8	$(TAC)_2$ GCC $(ATGT)_4$ $(TA)_2$	Interrupted complex	Intergenic region	CACAGATTCGCAGCGGATTA<br>TTGGTGAAGTTGTTTTGGTACG	57	36	204
154	Zeaxanthin epoxidase (ZEP)	HRO_ZE_1	PGSC0003<br>DMG400004020	PGSC0003<br>DMT400010287	PGSC0003<br>DMT400010287	2:43804309-43810444	6136	chr02:65059752..65065887 forward	6136	5265	2010	2	$(CT)_2$ ATG $(TA)_3$ CAA<br>$(TTA)_2$	Interrupted complex	3'-UTR	CCTGGAGAAAGTCATGGTCA<br>TAAGTCCCAGGAGGCAAATG	53	36	200

Table S4 Overview of 19 AFLP primer combinations used in the UPGMA dendrogram (Figure S1)

E-Name	E-Primer	E-IRD	M-Name	M-Primer
E32	E-AAC	800	M47	M-CAA
E32	E-AAC	800	M48	M-CAC
E32	E-AAC	800	M49	M-CAG
E32	E-AAC	800	M50	M-CAT
E32	E-AAC	800	M60	M-CTC
E32	E-AAC	800	M61	M-CTG
E32	E-AAC	800	M62	M-CTT
E33	E-AAG	800	M47	M-CAA
E33	E-AAG	800	M48	M-CAC
E33	E-AAG	800	M49	M-CAG
E33	E-AAG	800	M60	M-CTC
E33	E-AAG	800	M61	M-CTG
E33	E-AAG	800	M62	M-CTT
E35	E-ACA	800	M47	M-CAA
E35	E-ACA	800	M48	M-CAC
E35	E-ACA	800	M49	M-CAG
E35	E-ACA	800	M60	M-CTC
E35	E-ACA	800	M61	M-CTG
E35	E-ACA	800	M62	M-CTT

Table S5. Linkage group specific SSR primer combinations

References: (1) Feingold et al., 2005 [45]; (2) Ghislain et al., 2009 [51]; (3) Milbourne et al., 1998 [47]; (4) Provan et al., 1996 [48]; (5) Veilleux et al., 1995 [50]; (6) unpublished from SCRI, 2009.

LG	Primer	Sequence in 5'-3'-direction	Reference	Gene ID/ Reference	SSR	Expected size in bp
1	STG0016for	AgCTgCTCAgCATCAAgAgA	(2)	BI178934	$(AGA)_n$	138
	STG0016rev	ACCACCTCAggCACTTCATC				
1	STI0031for	AggCgCACTTTAACTTCCAC	(1)	(TC105688)	$(TCA)_n$	138
	STI0031rev	CggAACAAATggCTCTgATg				
1	STI0034for	CAAgAAACCAAgAgCAAATTTCA	(1)	(TC94397)	$(CTT)_n$	158
	STI0034rev	TggCgAATgTgAgAAACAAA				
1	STI0043for	CAATgCgAATgTTgCTACTggT	(1)	(TC95982)	$(AAC)_n$	135
	STI0043rev	ATCCACCAAgACCTCCAgAA				
1	STMLeatpacA	ACTTACTCCCCTCCAACTC	(3)	M96324	$(TA)_n$	187
	STMLeatpacA	CgTTTggTTACAAgAgAATTg				
2	STG0033for	gCTCATTTgACTgCTAAACCC	(2)	BI935647	$(CGG)_n$	-
	STG0033rev	gAAAgAATTgTgCCgTCgAT				
2	STI0024for	CgCCATTCTCTCAgATCACTC	(1)	(TC104888)	$(CAA)_n$	168
	STI0024rev	gCtgCAgCAgTTgTTgTTgT				
2	STI0036for	ggACTggCTgACCATgAACT	(1)	BI920053	$(AT)_n(TC)_{imp}$	-
	STI0036rev	TTACAggAAATgCAAACTTCg				
2	STI0056for	gACAgAgAATATgggACCACCA	(1)	(TC95818)	$(TA)_n$	195
	STI0056rev	gCAgCACCTTAAATggCTgAC				
2	STM5114for	AATggCTCTCTCTgTATgCT	(6)	[p102B19]	$(ACC)_n$	-
	STM5114rev	gCTgTCCCAACTATCTTTgA				
3	STG0010for	CgATCTCTgCTTTgCAggTA	(2)	BM407152	$(TG)_n$	175-192
	STG0010rev	gTTCATCACTACCgCCgACT				
3	STG0018for	ACCCgAATCCAAACCCTAAC	(2)	BI920777	$(CAA)_n$	134
	STG0018rev	AACCCgTgTCAACTTCTgCT				
3	STM1053for	CTCCCCATCTTAATGTTTC	(3)	AB022690	$(TA)n(ATC)_n$	171
	STM1053rev	CAACACAGCATACAGATCATC				
3	STM1058for	ACAATTTAATTCAAgAAgCTAgg	(3)	Z13992	$(ATT)_n$	113
	STM1058rev	CCAAATTTgTATACTTCAATATgA				
4	STG0008for	TCCTCgAAAAATTCCTCCAC	(2)	BE341755	$(GT)_n$	-
	STG0008rev	CgCCTTCTTCAACAATCCAT				
4	STG0020for	gTCATCCgAAgATggAggAA	(2)	BI405354	$(GAT)_n$	-
	STG0020rev	CTggACTgTCTTCACCACCA				
4	STI0001for	CAgCAAAATCAgAACCCgAT	(1)	CK860917	$(AAT)_n$	188
	STI0001rev	ggATCATCAAATTCACCgCT				
4	STI0012for	gAAgCgACTTCCAAAATCAgA	(1)	U69633	$(ATT)_n$	183
	STI0012rev	AAAgggAggAATAgAAACCAAAA				
5	STG0021for	gCCTACTgCCCAAAACATT	(2)	BQ506520	$(AAGA)_n$	136
	STG0021rev	ACTggCTgggAAgCATACAC				
5	STI0032for	gggAAgAATCCTgAAATgg	(1)	BQ120452	$(GGA)_n$	120
	STI0032rev	TgCTCTACCAATTAACggCA				

**5**	STM1031for	GTGTTTGTTTTTCTGTAT	(3)	X55748	(AT)$_n$	275
	STM1031rev	AATTCATTCCTCATCTCTA				
**5**	STM5149for	CCCAAACTAACCCTAATTAC	(6)	[p120M22]	(AAG)$_n$	-
	STM5149rev	GCGACTCTATTAATCACTTGAC				
**6**	STI0004for	GCTgCTAAACACTCAAgCAgAA	(1)	BQ118939	(AAG)$_n$	103
	STI0004rev	CAACTACAAgATTCCATCCACAg				
**6**	STI0021for	CATCAAgTCgTCgTCATCAA	(1)	TC94434	(CAT)$_n$	106
	STI0021rev	TCgAATgATCCAAAgCTTCC				
**6**	STM1019for	AgATTTTATTATTCCCAACAAgCA	(3)	L01402	(ATC)$_n$	-
	STM1019rev	CAACTACCTTCTCCCCACATAg				
**6**	STM5126for	gCAACAgCgCATCAACAAA	(6)	[p46L10]	(AAG)$_n$	-
	STM5126rev	TCCAAATCCATCCCATTgAg				
**7**	STI0025for	CTgCCgCAAAAAgTgAAAAC	(1)	BQ119932	(CTCC)$_n$	116
	STI0025rev	TgAATgTAggCCAAATTTTgAA				
**7**	STI0033for	gAgggTTTTCAgAAAgggA	(1)	BG886969	(AGG)$_n$	133
	STI0033rev	CATCCTTgCAACAACCTCCT				
**7**	STM0031for	CATACgCACgCACgTACAC	(3)	[PAC50]	(AC)$_n$...(AC)$_n$ GCAC (AC)$_n$ (GCAC)$_n$	168 - 211
	STM0031rev	TTCAACCTATCATTTTgTgAgTCg				
**7**	STM1004for	ATATgAAATTCTCTCgATgTTTCg	(3)	Z27235	(AAG)$_n$	163
	STM1004rev	TCAgCCCATAAAXCTTTAgTTACCT				
**7**	STM5144for	CAATgAgAATTgTggAggCAAg	(6)	[p104M6]	(TAA)$_n$..(TA)$_n$	-
	STM5144rev	gCATATAAATTCAAAgATCACg				
**8**	STGBSS2for	AATCggTgATAAATgTgAATgC	(4)	EU548082	(TCT)$_n$	121-150
	STGBSS2rev	ATgCTTgCCATgTgATgTgT				
**8**	STI0003for	ACCATCCACCATgTCAATgC	(1)	AW096896	(ACC)$_n$	144
	STI0003rev	CTCATggATggTgTCATTgg				
**8**	STM0024for	CATTACCTTgTgAgATTAgATTg	(3)	[PAC42]	(GTT)$_n$..(AC)$_n$ (AT)$_n$	-
	STM0024rev	CATATAAgTAggAATAggAggTTT				
**8**	STM1104for	TgATTCTCTTgCCTACTgTAATCg	(3)	EU548082	(TCT)$_n$	165
	STM1104rev	CAAAgTggTgTgAAgCTgTgA				
**8**	Stpatpgfor	CAACCAACAAggTAAATggTACC	(5)	X04077	(AATT)$_n$	386
	Stpatpgrev	TggTCTggTgCATTAgAAAAAA				
**9**	STG0011for	TTgCTCCTCCTCCACTTgAT	(2)	BQ046381	(AAC)$_n$	147
	STG0011rev	CACACACCTCAAATTggTCg				
**9**	STI0014for	AgAAACTgAgTTgTgTTTgggA	(1)	BQ115461	(TGG)$_n$(AGG)$_n$	129
	STI0014rev	TCAACAgTCTCAgAAAACCCTCT				
**9**	STM1051for	CCCCTTggCATTTTCTTCTCC	(3)	AJ133765	(TAT)$_n$ TTT(TAT)$_n$	226
	STM1051rev	TTTAgggTggggTgAggTTgg				
**9**	STM3012for	CAACTCAAACCAGAAGGCAAA	(3)	-	(CT)$_4$…(CT)$_8$	193
	STM3012rev	GAGAAATGGGCACAAAAAACA				
**10**	STG0025for	ggAATCCgAATTACgCTC	(2)	BQ506618	(AAAC)$_n$	-
	STG0025rev	AggTTTTACCACTCgggCTT				
**10**	STM1040for	AgTACTCAgTCAATCAAAg	(3)	Z11882	(A)$_n$	173
	STM1040rev	AggTAAgTATgTTCTCCAg				
**10**	STM5117for	CCACATTAGAAAACCCCTC	(6)	[p29E24]	(GAA)n	-

	STM5117rev	AGGACGCTGGTACATGTTAGA				
**10**	STM5132for	AgTTCACgAgAggTATCCATg	(6)	[p20H20]	$(TAA)_n$	-
	STM5132rev	gAAATTCAgATCCACCgCAA				
**11**	STG0001for	CAgCCAACATTTgTACCCCT	(2)	BE340539	$(CT)_n$	126
	STG0001rev	ACCCCCACTTgCCATATTTT				
**11**	STG0004for	gAAAgCCAATCTCACTggA	(2)	BI434815	$(GT)_n$	212-217
	STG0004rev	TATAATTggCTTgCgAgTgC				
**11**	STI0018for	CCACTACTgCTTCCTCCACC	(1)	(TC98351)	$(GTT)_n$	189
	STI0018rev	gCAgCAACAACAAgCTCAAC				
**11**	STI0028for	ATACCCTCCAATgggTCCTT	(1)	(TC97044)	$(CAA)_{imp}$	170-217
	STI0028rev	CTTggAgATttgCAAgAAgAA				
**11**	STM5130for	AAAgTACAgCgAAgATgACgAC	(6)	[p2115]	$(CGG)_n$	-
	STM5130rev	TTACCTTTgCAACCTTgCC				
**12**	STI0030for	gACCCTCCAACTATAgATTCTTC	(1)	BF188393	$(ATT)_n$	105
	STI0030rev	TgACAACTTTAAAgCATATgTCAgC				
**12**	STI0051for	ggTCTCCATTAgCCCTCTgAg	(1)	(TC96682)	$(TA)_n$	-
	STI0051rev	ACATAAATggATCACACA				
**12**	STI0063for	gCATTCTATggCCAACATTgg	(1)	(T_AW030425)	$(GAT)_{imp}$	252
	STI0063rev	AgATTCCTCCAATTTCCCAgC				
**12**	STM0030for	AGAGATCGATGTAAAACACGT	(3)	[PAC05]	compound (GT/GC) $(GT)_n$	-
	STM0030rev	GTGGCATTTTGATGGATT				
**12**	STM5121for	CACCggAATAAgCggATCT	(6)	[p46L17]	$(TGT)_n$	297-309
	STM5121rev	TCTTCCCTTCCATTTgTCA				

Table S6. Selected 23 transcripts identified in Sprenger et al. [39]

Transcript name	PGSC-ID (DMT)	PGSC-ID (DMG)	Marker name
Glutathione S-transferase	PGSC0003DMT400004539	PGSC0003DMG400001804	HRO_GLUTST
Bacterial spot disease resistance protein 4	PGSC0003DMT400006231	PGSC0003DMG400002427	HRO_BSDRP4
Beta-D-glucan exohydrolase	PGSC0003DMT400015224	PGSC0003DMG402005942	HRO_BDGEH
SBT4B protein	PGSC0003DMT400017633	PGSC0003DMG400006840	HRO_SBT4B
Ethylene-inducing xylanase	PGSC0003DMT400020366	PGSC0003DMG400007876	HRO_EIX
Glucosyltransferase	PGSC0003DMT400021019	PGSC0003DMG400008138	HRO_GLUCT
Serine-threonine protein kinase, plant-type	PGSC0003DMT400028434	PGSC0003DMG400010951	HRO_STPKPT
Multidrug resistance protein ABC transporter family	PGSC0003DMT400031714	PGSC0003DMG400012167	HRO_MRPATF
BED finger-nbs-lrr resistance protein	PGSC0003DMT400035714	PGSC0003DMG400013736	HRO_BFNBLRP
S-locus-specific glycoprotein S6	PGSC0003DMT400036026	PGSC0003DMG401013876	HRO_SLSGPS6
Kinase	PGSC0003DMT400042362	PGSC0003DMG400016429	HRO_KIN
Receptor protein kinase	PGSC0003DMT400045689	PGSC0003DMG400017718	HRO_RPK
UDP-glucose:glucosyltransferase	PGSC0003DMT400049125	PGSC0003DMG400019093	HRO_UDPGGT
Methionyl-tRNA synthetase	PGSC0003DMT400049172	PGSC0003DMG400019113	HRO_MTRNAS
Flagellin-sensing 2	PGSC0003DMT400068776	PGSC0003DMG401026747	HRO_FSL2
TIR-NBS-LRR disease resistance	PGSC0003DMT400071445	PGSC0003DMG400027797	HRO_TNLDR
LRR receptor-like serine/threonine-protein kinase	PGSC0003DMT400071885	PGSC0003DMG400027968	HRO_LRRSTPK
Poly (ADP-ribose) glycohydrolase	PGSC0003DMT400075512	PGSC0003DMG400029361	HRO_PARGH
Bacterial spot disease resistance protein 4	PGSC0003DMT400080813	PGSC0003DMG400031476	HRO_BSDRP4
Extensin	PGSC0003DMT400082012	PGSC0003DMG401032203	HRO_EXT
Lipoxygenase	PGSC0003DMT400082023	PGSC0003DMG400032207	HRO_LIPOX
Betaine aldehyde dehyrogenase	PGSC0003DMT400083025	PGSC0003DMG400033028	HRO_BADH
Vacuolar protein sorting protein	PGSC0003DTM400008981	PGSC0003DMG400003501	HRO_VPSP

Table S7. Characterization of 75 polymorphic SSR primer combinations

Marker Name	Reference - Gene ID[1] PGSC0003	Chromosome location	Repeat Motif	Expected product size[2] in bp	Observed product size in bp	Allele sizes in bp	Total number of alleles	PIC value	H_e value	Scoring efficiency[3]
HRO_ABA8H4_2	DMG400007972	IV	$(GT)_2$ $(AT)_7$	200	219-224	219, 223, 224	3	0.4117	0.4998	1
HRO_ACO_1	DMG400021476	II	$(AT)_8$	134	140-156	140, 144, 149, 156	4	0.6132	0.6762	1
HRO_ACO_4	DMG400009719	IX	$(GA)_{12}$	172	175-191	175, 177, 180,181, 184, 191	6	0.6692	0.7203	1
HRO_ACS_2	DMG400000284	XII	$(AT)_6$	139	157-178	157, 161, 178	3	0.3	0.3254	1
HRO_ACS2_1	DMG400000193	I	$(TTA)_5$ T $(TAA)_9$	211	202-238	202, 206, 213, 221, 226, 238	6	0.6939	0.7387	1
HRO_ACS3	DMG400021426	II	$(AT)_9$	159	170-181	170, 173,174, 177,181	5	0.6546	0.7064	1
HRO_ACS4_1	DMG400021651	V	$(ACT)_5$	104	118-120	118, 120	2	0.2896	0.3512	1
HRO_ALDH	DMG400034597	IX	$(AT)_{12}$ $(AG)_{12}$ ATA $(GA)_9$	177	156-199	156, 159, 162, 163, 166,169, 175, 176, 179, 181, 184, 186, 188, 190, 192, 196, 197, 199	18	0.9003	0.9075	2
HRO_APX_2	DMG401001731	IX	$(TA)_3$ C $(TA)_3$	174	196-202	196, 198, 202	3	0.3741	0.4159	3
HRO_ARF6_1	DMG400028826	XII	$(CT)_2$ $(TAAAT)_2$ $(GAA)_2$	219	238-243	238, 243	2	0.1706	0.1884	1
HRO_BA_1	DMG400000169	I	$(GAG)_2$ AAA $(GAA)_3$ $(GA)_3$A $(GAA)_2$	213	207-268	207, 213, 251, 255, 264, 266,268	7	0.72	0.7603	1
HRO_BADH_2	DMG400033028	VI	$(GT)_3$ CA $(GT)_4$	195	209-219	209, 212, 214, 219	4	0.5975	0.6682	1
HRO_BDGEH_1B	DMG402005942	VI	$(GT)_2$ $(TA)_2$ CATGG $(AT)_4$	138	159-161	159, 161	2	0.2392	0.2778	1
HRO_BFNLRP_2C	DMG400013736	XII	$(TA)_2$ GACTA $(TC)_2$ ATTC $(TTTA)_2$	175	196-197	196, 197	2	0.1046	0.1107	2
HRO_BSDRP4_5A	DMG400002427	VI	$(TCT)_4$ GTG $(AGT)_2$	132	155-172	155, 164, 166, 169, 172	5	0.7664	0.7986	2
HRO_BSDRP4_5C	DMG400002427	VI	$(TTTA)_5$ T $(TA)_2$	158	157-180	157, 162, 172, 175, 177, 179, 180	7	0.7866	0.8136	1
HRO_BSDRP4_9C	DMG400031476	XI	$(CT)_2$ $(TA)_3$ T $(TAA)_2$	152	175-194	175,194	2	0.3726	0.4953	2
HRO_CTR1K	DMG400008384	X	$(TA)_2$ $(TAT)_5$	197	220-223	220, 221, 223	3	0.5157	0.5848	2
HRO_D1P5CS_1	DMG402026767	VI	$(TTA)_{10}$	187	202-216	202, 205, 207, 208, 216	5	0.6065	0.6534	1
HRO_D1P5CS_2	DMG402026767	VI	$(CT)_5$ T $(TA)_2$	168	189-200	189, 200	2	0.2225	0.255	1
HRO_EBF1_1a	DMG400030928	VII	$(CT)_3$ T $(CAA)_3$	177	219-227	219, 223, 227	3	0.4798	0.5568	1

HRO_EBF1_2	DMG400002914	XII	$(AT)_2$ $(TGTC)_2$ GA $(TGT)_2$	181	202-272	202, 267, 272	3	0.5274	0.6099	1
HRO_EIL2_1	DMG400008712	I	$(ACCCTC)_2$ $(CT)_5$	132	153-180	153, 161, 166, 172, 180	5	0.6076	0.6518	1
HRO_EIN2_1A	DMG400021547	IX	$(TC)_7$ $(AT)_2$ $(GT)_9$	183	204-226	204, 206, 210, 211, 215, 219, 224, 226	8	0.7979	0.8214	2
HRO_EIX_1E	DMG400007876	XII	$(GT)_5$ GGGG $(CA)_2$	149	168-192	168, 169, 171, 192	4	0.4486	0.5488	2
HRO_EREBP_1A	DMG400001067	XII	$(CTT)_2$ $(TC)_6$ CCC $(CTG)_2$	178	196-208	196, 199, 208	3	0.4136	0.5146	2
HRO_EREBP1_1	DMG400029713	II	$(GTGTT)_4$ $(GTTTT)_2$ $(GT)_2$ $(TG)_3$	200	193-230	193, 205, 206, 212, 219, 220, 224, 230	8	0.6028	0.6531	2
HRO_EREBPC2_1	DMG400016003	V	$(GTTG)_2$ AA $(TAT)_2$ GAAA $(GAATT)_2$	202	224-246	224, 225, 226, 239, 246	5	0.6758	0.7267	2
HRO_ERF114_1	DMG400014240	III	$(CTT)_3$ CAT $(CTT)_2$	211	219-231	219, 231	2	0.3297	0.4163	1
HRO_ERF141	DMG400028853	XII	$(GA)_4$ $(TTTTTA)_2$	199	214-302	214, 217, 283, 297, 302	5	0.6891	0.736	1
HRO_ERF4_1	DMG400016004	V	$(TAA)_2$ $(GAA)_3$ TTAC $(AG)_2$	197	218-219	218, 219	2	0.1379	0.149	2
HRO_ERS1_1	DMG400031819	IX	$(GCA)_5$ $(GC)_2$	190	182-210	182, 183, 191, 196, 205, 207, 210	7	0.7399	0.776	1
HRO_ETOP1_3	DMG400015898	X	$(AT)_2$ CATAAT $(CCT)_3$	189	210-213	210, 211, 213	3	0.5866	0.6609	1
HRO_ETR1_1A	DMG400007843	XII	$(ATA)_2$ AAA $(ATA)_4$	180	186-196	186, 189, 193, 196	4	0.6108	0.6669	1
HRO_ETRHOM_2A	DMG400028694	XI	$(GT)_4$ A $(TG)_6$	127	142-154	142, 152, 154	3	0.4667	0.5552	1
HRO_ETRTF_5a	DMG400002185	XI	$(CCA)_4$ $(ACA)_3$	198	217-221	217, 218, 220, 221	4	0.6711	0.7218	1
HRO_ETRTF13_1A	DMG400026046	I	$(CTT)_3$ $(CTC)_5$	185	207-209	207, 209	2	0.1379	0.149	2
HRO_ETRTF3_2	DMG400014417	X	$(CGG)_7$	169	177-191	177, 183, 186, 191	4	0.6317	0.6882	1
HRO_EXT_1D	DMG401032203	I	$(ACC)_2$ TC $(CAC)_3$ GAT $(CAC)_3$ CT $(CCA)_3$	199	190-224	190, 195, 209, 211, 224	5	0.6084	0.6761	2
HRO_FLS2_1A	DMG401026747	III	$(AT)_3$ $(TAT)_2$ $(GA)_2$	142	155-164	155, 159, 164	3	0.4114	0.4859	1
HRO_G3O_1	DMG400016516	VI	$(ACC)_5$ $(AAT)_3$	152	170-177	170, 173, 177	3	0.4737	0.5493	1
HRO_GLUCT_7C	DMG400008138	X	$(TC)_7$ TGTT $(TC)_3$	214	232-252	232, 239, 247, 249, 252	5	0.5966	0.662	1
HRO_GLUTST_1A	DMG400001804	I	$(AT)_4$ G $(TA)_2$	153	177	177	1	0.00	0.00	1
HRO_GLUTST_1C	DMG400001804	I	$(GT)_4$ AT $(TAG)_2$	131	154	154	1	0.00	0.00	1
HRO_JA2_1	DMG400015342	XII	$(TA)_2$GT $(TG)_2$ $(TAA)_3$AA $(TGA)_2$	223	210-228	210, 228	2	0.3318	0.42	2

HRO_KIN_1	DMG400016429	I	$(TC)_2$ AAGC $(AT)_4$	132	151-158	151, 154, 158	3	0.5917	0.6657	2
HRO_LEA5_1	DMG400017936	VI	$(TA)_3$ A $(TA)_2$ A $(TA)_2$	142	156-167	156, 158, 161, 162, 166, 167	6	0.7134	0.7491	2
HRO_LIPOX_1B	DMG400032207	I	$(TC)_3$ TA $(CT)_2$	135	158-159	158, 159	2	0.3576	0.4664	2
HRO_LRRSTPK_1C	DMG400027968	IV	$(AT)_3$ $(CATAT)_2$ A $(ATATC)_2$ $(AT)_3$	203	209-249	209, 219, 221, 224, 228, 239, 249	7	0.6569	0.7112	2
HRO_MOCOS_1A	DMG400022125	VII	$(TCT)_3$ G $(CA)_2$ ATTT $(AT)_2$ TGGT $(TG)_2$	184	194-208	194, 204, 208	3	0.3491	0.4002	1
HRO_MRPATF_3D	DMG400012167	VIII	$(ATA)_2$ TA $(AT)_6$ $(GAT)_2$	163	185	185	1	0.00	0.00	1
HRO_MTRNAS_1D	DMG400019113	V	$(AT)_3$ AC $(AT)_3$ $(GT)_3$	131	151-161	151, 154, 159, 161	4	0.5611	0.6299	1
HRO_NCED_2	DMG400027633	VII	$(GA)_3$G $(TA)_4$	145	168-170	168, 170	2	0.3675	0.4851	2
HRO_NCED_3	DMG400004311	VIII	$(TA)_3$ $(TGTTT)_2$ $(CTT)_3$	134	151-162	151, 158, 159, 162	4	0.2217	0.2327	1
HRO_NDPK2_1	DMG400026976	VI	$(AT)_4$ $(TTA)_2$	182	202-204	202, 204	2	0.3606	0.4721	1
HRO_P5CR_2	DMG400010441	II	$(TA)_3$ $(TAAAA)_2$	184	202-204	202, 204	2	0.3749	0.4998	1
HRO_PARGH_1A	DMG400029361	XII	$(GA)_4$ AA $(GA)_2$	135	147-156	147, 156	2	0.3201	0.4002	1
HRO_PARGH_1C	DMG400029361	XII	$(TA)_2$ G $(TA)_8$	175	192-195	192, 193, 195	3	0.4023	0.4444	1
HRO_POD1_2	DMG400010050	II	$(TG)_3$ $(AT)_4$	117	130-145	130, 139, 141, 143, 145	5	0.6375	0.6932	1
HRO_PP2C	DMG400011321	I	$(GAA)_3$ A $(AT)_2$ CAT $(GAT)_2$	182	196-205	196, 205	2	0.3346	0.4248	1
HRO_PP2C1_2	DMG400032110	I	$(GGT)_2$ $(GT)_2$ $(CT)_3$	149	168-173	168, 171, 173	3	0.4126	0.5103	1
HRO_RPK_10D	DMG400017718	IV	$(TC)_2$ CCA $(AT)_3$ TTC $(CA)_2$	158	180-191	180, 182, 190, 192	4	0.4671	0.5637	2
HRO_SBT4B_1B	DMG400006840	I	$(AG)_2$ G $(TAT)_2$ T $(GTA)_3$	101	103-124	103, 124	2	0.1986	0.2235	1
HRO_SIPP_1	DMG400012223	VIII	$(ATC)_2$ AC $(AT)_5$	206	224-234	224, 228, 230, 232, 234	5	0.6094	0.677	1
HRO_SLSGPS6_5C	DMG401013876	II	$(ATA)_2$ $(AAT)_4$ ACCT $(TA)_2$	183	205-227	206, 215, 227	3	0.3213	0.372	1
HRO_STPK	DMG400000652	XII	$(CT)_3$ TTT $(CT)_2$ TT $(CT)_3$	178	161-183	161, 171, 174, 180, 183	5	0.6796	0.7259	1
HRO_STPKPT_1A	DMG400010951	0/ III	$(TG)_2$ $(TA)_2$ AATCA $(CT)_2$	115	129-142	129, 138, 142	3	0.4318	0.4906	1
HRO_STPKPT_1B	DMG400010951	0/ III	$(AGT)_2$ $(TG)_3$	166	188-193	188, 193	2	0.1706	0.1884	2
HRO_TNLDR_2A	DMG400027797	XI	$(AT)_4$ AAA $(TGA)_2$	164	170-186	170, 186	3	0.3936	0.5048	1

HRO_TPS1_1	DMG400007089	VII	$(TA)_3$ $(CA)_3$	156	178-180	178, 180	2	0.369	0.4882	2
HRO_UDPGGT_1A	DMG400019093	0	$(TG)_2$ ATT $(GA)_2$ T $(TGGG)_2$	141	151-164	151, 164	2	0.2896	0.3512	1
HRO_UGP_1	DMG401013333	XI	$(TA)_2$ $(GT)_8$	177	196-199	196, 199	2	0.3508	0.4537	1
HRO_VPSP_1B	DMG400003501 (SPUD DB)	0	$(TGG)_2$ ACT $(AT)_2$ G $(GA)_4$	157	182-183	182, 183	2	0.3538	0.4592	2
HRO_WRKYTF_1B	DMG400008188	VIII	$(TAC)_2$ GCC $(ATGT)_4$ $(TA)_2$	204	230-231	230, 231	2	0.355	0.4614	2
HRO_ZE_1	DMG400004020	II	$(CT)_2$ ATG $(TA)_3$ CAA $(TTA)_2$	200	216-222	216, 218, 219, 222	4	0.4949	0.5686	2

[1]Phytozome

[2]without M13 forward tail (labelled with fluorescent IRD700 or IRD800 M13 primer)

[3]1 = Easy to score, 2 = Average to score, 3 = Bad or no scoring

Table S8. Association studies applying the exact Fisher-Test to the SSR-marker results in the potato association panel (1t most tolerant cultivar, 34t most sensitive cultivar). *significant p-value (< 0.05) shown in grey

Primer\Cultivar	1t	2t	3t	4t	5t	6t	7t	8t	9t	10t	11t	12t	13t	14t	15t	16t	17t	18t	19t	20t	21t	22t	23t	24t	25t	26t	27t	28t	29t	30t	31t	32t	33t	34t	p-value*
HRO_ABA8H4_2_A	1	1	1	0	1	1	1	1	1	1	1	0	1	1	1	1	1	1	1	1	1	1	1	1	1	1	1	1	1	0	0	0	0	1	0.6562
HRO_ABA8H4_2_B	0	0	0	0	0	0	0	0	0	0	0	0	0	0	0	0	0	0	0	0	0	0	0	0	0	0	0	0	0	0	1	1	0	0	0.4848
HRO_ABA8H4_2_C	0	0	0	1	1	0	1	1	1	1	0	1	1	0	1	0	1	0	0	0	0	0	1	0	0	0	0	0	0	1	1	1	1	0	0.1663
HRO_ACO_1_A	1	1	1	1	1	1	0	1	1	0	1	0	1	1	1	0	1	1	1	1	1	1	1	1	1	1	0	1	1	1	0	1	1	0	1.0000
HRO_ACO_1_B	1	1	1	0	1	1	1	1	1	1	1	1	1	1	1	1	1	1	1	0	1	1	1	1	1	1	1	1	1	1	0	0	1	1	0.6012
HRO_ACO_1_C	0	0	1	1	0	0	0	0	0	1	0	1	0	0	0	0	0	0	0	0	0	0	0	0	0	0	0	0	0	0	0	0	0	0	0.1026
HRO_ACO_1_D	1	1	0	1	0	1	0	0	0	1	1	0	1	1	0	0	0	1	0	1	1	1	0	0	1	0	0	0	0	1	1	0	0	0	1.0000
HRO_ACO_4_A	0	1	1	1	0	1	1	1	0	1	1	1	1	1	0	1	1	1	0	1	1	1	1	1	0	1	1	0	1	1	1	0	0	1	1.0000
HRO_ACO_4_B	0	0	0	0	0	0	0	0	0	0	0	0	0	0	0	0	0	0	0	0	0	0	0	0	0	0	0	0	0	0	1	0	0	0	1.0000
HRO_ACO_4_C	1	1	0	1	1	1	1	1	1	1	1	0	1	1	1	1	1	1	1	1	1	1	1	1	1	0	1	1	0	0	0	1	1	1	0.6562
HRO_ACO_4_D	0	0	0	0	0	1	0	0	0	0	0	0	1	0	0	0	1	0	0	1	0	0	0	0	0	0	0	0	0	0	0	0	0	0	0.6012
HRO_ACO_4_E	1	1	1	0	0	1	1	1	0	0	1	1	0	1	0	0	0	1	0	1	0	1	0	0	1	1	0	1	1	1	1	1	1	0	0.7283
HRO_ACO_4_F	1	0	0	0	0	0	0	0	0	0	0	0	0	0	0	0	0	0	0	0	0	0	0	0	1	0	0	0	0	0	0	1	0	0	1.0000
HRO_ACS_2_A	0	1	0	0	0	0	0	0	0	0	0	0	0	0	0	0	0	0	0	0	0	0	1	0	0	0	0	0	0	1	0	0	0	0	1.0000
HRO_ACS_2_B	1	1	0	0	0	0	0	0	0	0	1	0	0	0	0	0	0	0	0	1	0	0	0	0	1	0	0	0	0	0	0	0	0	0	1.0000
HRO_ACS_2_C	1	1	1	1	1	1	1	1	1	1	1	1	1	1	1	1	1	1	1	1	1	1	1	1	1	1	1	1	1	1	1	1	1	1	1.0000
HRO_ACS2_A	1	1	1	1	1	0	1	1	1	1	1	0	0	0	1	1	1	0	1	1	1	1	1	1	1	1	1	1	0	0	1	1	1	1	1.0000
HRO_ACS2_B	0	0	1	0	0	1	1	0	1	1	0	1	1	1	0	0	1	1	0	1	1	1	0	1	0	0	0	1	1	1	0	1	0	0	1.0000
HRO_ACS2_C	1	1	0	1	1	1	1	0	1	1	1	1	1	1	0	1	0	1	1	1	0	0	1	1	1	1	1	1	1	1	1	0	1	1	1.0000
HRO_ACS2_D	0	1	0	0	0	0	0	0	0	0	1	0	0	0	0	0	0	0	0	0	1	1	0	0	1	0	0	0	1	0	0	0	0	1	0.3983
HRO_ACS2_E	0	0	0	0	0	0	0	0	0	0	0	0	0	1	1	0	0	0	0	0	0	0	0	0	0	0	0	0	0	0	0	0	0	0	0.4848
HRO_ACS2_F	0	0	1	0	0	0	0	0	0	0	0	0	0	0	0	1	0	0	0	0	0	0	0	0	0	0	1	0	0	0	0	0	0	0	1.0000
HRO_ACS3_A	0	0	0	1	0	0	1	1	1	1	1	1	0	1	1	1	0	1	1	0	0	0	1	1	0	0	1	1	1	1	1	1	1	1	0.7207
HRO_ACS3_B	1	1	1	1	1	1	1	1	1	1	1	1	1	1	1	0	1	1	1	1	1	1	1	1	1	1	1	1	1	1	1	1	1	1	1.0000
HRO_ACS3_C	0	0	0	0	0	0	0	0	1	0	0	0	1	0	0	1	1	0	0	0	0	0	0	0	0	1	0	1	0	0	0	0	0	0	0.6562
HRO_ACS3_D	0	0	1	0	1	1	1	0	0	0	0	0	0	1	1	0	0	1	1	1	1	1	1	0	0	0	1	1	1	1	1	1	1	0	0.0366
HRO_ACS3_E	0	0	0	0	0	0	1	0	0	0	0	0	0	0	1	0	0	0	0	0	0	0	0	0	0	0	0	0	0	0	0	0	0	0	0.4848
HRO_ACS4_A	1	1	1	1	1	1	1	1	1	1	1	1	1	1	1	1	1	1	1	1	1	1	1	1	1	1	1	1	1	1	1	1	1	1	1.0000
HRO_ACS4_B	0	0	0	0	0	1	1	1	0	0	1	0	0	1	0	0	0	0	0	1	0	0	1	1	0	1	0	1	0	0	0	0	0	0	1.0000
HRO_ALDH_A	0	1	0	0	0	1	0	1	1	0	1	1	1	1	1	0	1	0	1	1	0	0	1	1	0	1	0	1	1	1	1	0	0	1	1.0000
HRO_ALDH_B	1	1	1	0	1	1	1	1	0	1	1	1	1	1	1	1	1	1	0	1	1	1	1	1	1	1	1	1	1	1	1	0	1	1	1.0000
HRO_ALDH_C	0	1	1	1	1	1	0	0	0	0	0	1	0	0	1	1	1	1	0	0	1	1	1	0	0	0	0	0	0	0	1	0	0	0	0.2960
HRO_ALDH_D	0	0	0	0	0	0	0	0	0	1	0	1	0	1	0	0	0	0	0	0	0	0	1	0	0	0	0	0	0	0	0	0	0	1	1.0000
HRO_ALDH_E	0	0	0	0	0	0	0	0	1	0	1	0	0	0	0	0	0	0	0	0	0	0	0	1	0	0	1	0	0	0	0	1	0	0	1.0000
HRO_ALDH_F	1	0	0	0	0	0	0	0	0	0	0	0	0	0	0	0	0	0	0	0	0	0	0	0	0	0	0	0	0	0	0	0	0	1	1.0000
HRO_ALDH_G	1	0	0	0	0	0	1	0	1	0	0	1	0	0	0	0	0	0	1	1	0	0	0	0	1	0	0	0	0	0	0	0	1	0	1.0000
HRO_ALDH_H	1	1	1	0	0	0	0	0	0	0	0	0	0	0	0	1	0	0	1	1	1	0	0	1	0	1	1	1	1	1	0	1	0	1	0.0366
HRO_ALDH_I	0	0	0	0	0	0	0	1	0	0	0	0	1	0	0	0	0	0	0	0	0	0	0	0	0	0	0	0	0	0	0	0	0	0	0.4848

HRO_ALDH_J	0	0	0	0	0	0	0	0	1	1	0	1	0	1	0	1	1	0	1	0	0	0	0	0	0	1	0	0	1	0	0	0	0	0	0.4384
HRO_ALDH_K	1	1	1	1	1	1	1	1	1	1	1	1	1	1	1	1	1	1	1	1	1	1	1	1	1	1	1	1	1	1	1	1	1	1	1.0000
HRO_ALDH_L	0	0	0	0	0	0	0	0	0	0	0	0	0	0	0	0	0	0	1	0	0	0	0	1	1	0	0	0	0	0	0	0	0	0	0.2273
HRO_ALDH_M	0	0	0	0	0	1	1	0	0	0	0	0	0	1	0	0	0	0	0	0	0	0	0	1	0	0	0	0	0	0	0	0	0	0	0.6012
HRO_ALDH_N	0	0	0	1	1	1	1	1	0	1	0	1	0	1	1	1	1	1	1	1	1	0	1	1	1	1	1	1	1	1	1	1	1	1	0.0854
HRO_ALDH_O	1	1	0	0	0	0	0	0	0	0	0	0	0	0	0	0	0	0	1	0	0	0	0	0	0	1	0	0	0	0	0	0	0	0	1.0000
HRO_ALDH_P	0	1	0	0	0	0	0	1	0	1	0	0	0	0	0	0	0	1	1	1	0	0	1	0	1	0	1	0	0	1	0	1	0	0	0.1411
HRO_ALDH_Q	1	0	0	0	1	1	1	1	1	0	1	0	0	0	1	0	1	1	1	1	1	0	0	0	1	0	0	1	1	1	1	1	0	1	0.7283
HRO_ALDH_R	0	0	0	0	0	0	0	0	0	0	0	0	0	0	0	0	1	0	0	0	1	0	0	0	0	0	0	0	1	1	0	0	0	1	0.3353
HRO_APX_2_A	0	0	0	0	0	0	0	0	0	1	0	0	1	0	0	0	0	0	0	0	0	0	0	1	0	1	0	0	0	0	0	0	0	0	1.0000
HRO_APX_2_B	1	1	1	1	1	1	1	1	1	1	1	1	1	1	1	1	1	1	1	1	1	1	1	1	1	1	1	1	1	1	1	1	1	1	1.0000
HRO_APX_2_D	0	0	0	0	0	1	0	0	1	0	0	0	0	0	0	0	0	1	1	0	0	1	0	0	0	0	0	0	0	1	1	0	0	1	0.2245
HRO_ARF6_A	1	1	1	1	1	1	1	1	1	1	1	1	1	1	1	1	1	1	1	1	1	1	1	1	1	1	1	1	1	1	1	1	1	1	1.0000
HRO_ARF6_B	0	0	0	0	0	0	1	0	0	1	0	0	0	0	1	0	0	0	0	0	0	0	0	0	0	0	0	0	0	0	1	0	0	0	0.6012
HRO_BA_1_A	0	0	0	0	0	0	0	0	0	0	0	0	0	0	0	1	0	0	0	0	0	0	0	0	0	0	1	0	0	0	0	0	0	0	1.0000
HRO_BA_1_B	1	1	1	1	1	0	1	1	1	1	1	1	0	0	1	0	1	0	1	0	1	1	1	1	1	1	0	1	1	0	1	1	1	1	1.0000
HRO_BA_1_C	0	1	0	0	0	0	0	1	1	0	0	0	0	0	0	1	0	1	0	1	1	0	1	0	1	1	1	0	0	1	0	0	1	1	0.0799
HRO_BA_1_D	0	0	1	0	0	0	0	0	0	0	0	0	0	0	0	0	0	0	0	0	0	0	0	0	0	0	0	0	0	0	0	0	0	0	1.0000
HRO_BA_1_E	0	0	0	0	0	0	0	0	1	0	0	0	0	0	0	0	0	0	0	0	0	0	0	0	0	0	0	0	0	0	0	0	0	0	1.0000
HRO_BA_1_F	1	0	0	1	1	1	1	0	1	1	1	1	1	1	0	1	1	1	1	1	0	1	0	1	0	0	0	1	0	1	1	1	0	0	0.2818
HRO_BA_1_G	0	0	1	0	0	1	1	0	1	1	0	1	1	1	1	0	0	1	1	1	1	1	0	1	0	0	0	1	1	0	0	0	0	0	1.0000
HRO_BADH_2_A	1	0	1	1	1	1	0	1	1	1	1	1	1	1	1	1	1	0	1	1	1	1	1	1	1	1	1	1	0	1	1	1	1	1	1.0000
HRO_BADH_2_B	0	1	1	1	1	1	0	1	1	1	1	1	0	1	1	1	1	1	1	0	1	0	1	0	1	1	1	1	1	1	0	1	1	1	1.0000
HRO_BADH_2_C	1	1	0	1	0	1	1	1	1	0	1	1	1	0	0	1	1	1	0	1	1	0	0	1	0	1	0	1	1	1	0	0	0	1	0.4813
HRO_BADH_2_D	0	0	0	0	0	0	0	0	0	0	0	0	0	0	0	0	0	0	0	0	0	0	0	0	0	0	0	0	0	0	1	0	0	0	1.0000
HRO_BDGEH_1B_A	1	1	0	0	1	0	0	1	1	0	1	1	0	1	0	1	1	0	0	0	1	0	1	0	1	1	1	1	1	1	1	0	0	1	1.0000
HRO_BDGEH_1B_B	0	1	0	0	0	0	0	0	0	0	0	0	0	0	0	0	0	0	1	0	0	1	1	0	0	0	0	0	0	0	0	0	0	0	0.6012
HRO_BFNBLRP_2C_A	0	0	1	0	1	0	0	0	1	1	1	1	0	1	0	0	1	0	0	0	1	1	1	1	1	1	0	0	1	0	1	0	0	0	1.0000
HRO_BFNBLRP_2C_B	0	0	0	0	0	0	1	0	0	0	0	0	0	0	0	0	0	0	0	0	0	0	0	0	0	0	0	0	0	0	0	0	0	0	1.0000
HRO_BSDRP4_5A_A	1	1	1	1	1	1	1	1	1	0	1	1	1	0	1	1	1	1	1	1	1	1	1	1	1	1	1	1	0	1	1	1	1	1	1.0000
HRO_BSDRP4_5A_B	1	1	1	1	1	1	1	1	1	1	1	1	1	1	1	1	1	1	1	1	1	1	1	1	1	1	1	1	1	1	1	1	1	1	1.0000
HRO_BSDRP4_5A_C	1	1	1	1	1	1	1	1	1	1	1	1	1	1	1	1	1	1	1	1	1	1	1	1	1	1	1	1	1	1	1	1	1	1	1.0000
HRO_BSDRP4_5A_D	0	1	1	1	1	1	0	1	1	1	1	1	1	0	0	1	0	1	1	1	1	1	1	1	1	1	1	0	1	1	0	1	1	1	0.3983
HRO_BSDRP4_5A_E	1	1	1	1	1	1	1	1	1	1	1	1	1	0	1	1	1	1	1	1	1	1	1	1	1	1	1	1	1	1	1	1	1	1	1.0000
HRO_BSDRP4_5C_A	0	0	0	0	0	0	0	0	0	0	0	0	1	0	0	0	1	0	0	0	0	0	0	0	0	0	0	0	0	0	0	0	0	0	0.4848
HRO_BSDRP4_5C_B	1	1	1	0	1	1	1	1	1	0	0	0	0	0	1	0	0	1	0	0	1	1	1	1	0	0	0	1	0	1	1	1	1	0	1.0000
HRO_BSDRP4_5C_C	0	0	0	0	0	0	0	0	0	1	0	0	1	0	0	0	1	0	0	0	0	0	0	0	0	0	0	0	0	0	0	0	1	0	0.6012
HRO_BSDRP4_5C_D	0	0	1	1	0	0	0	1	0	0	0	0	0	0	1	0	1	0	1	1	1	0	1	0	1	0	1	1	1	1	1	0	0	1	0.0844
HRO_BSDRP4_5C_E	0	1	1	1	1	1	0	1	1	1	1	1	1	0	0	1	1	1	1	1	1	1	0	1	1	1	1	0	1	1	0	1	1	1	1.0000
HRO_BSDRP4_5C_F	1	0	1	0	1	1	1	0	0	0	1	0	1	1	0	1	1	0	1	1	0	1	0	0	1	1	1	1	1	0	0	0	0	1	1.0000
HRO_BSDRP4_5C_G	0	1	1	0	0	1	0	1	1	1	0	1	1	1	0	1	1	1	1	1	0	0	1	1	1	1	0	0	1	1	1	1	1	0	1.0000
HRO_BSDRP4_9C_A	0	1	1	1	1	1	0	1	1	1	1	1	1	0	0	1	1	1	1	1	1	1	1	1	1	1	1	0	1	1	0	1	1	1	0.6562
HRO_BSDRP4_9C_B	1	1	1	1	1	1	1	1	1	1	1	1	1	1	1	1	1	1	1	1	1	1	1	1	1	1	1	1	1	1	1	1	1	1	1.0000

HRO_CTR1K_A	0	0	1	1	0	1	0	0	1	0	1	0	0	0	1	1	1	1	0	0	0	1	0	0	0	1	0	1	0	1	1	1	1	1	1.0000
HRO_CTR1K_B	1	0	0	0	0	0	0	1	0	0	1	0	1	0	0	0	1	0	0	0	0	0	0	1	0	1	0	1	1	0	0	0	0	1	1.0000
HRO_CTR1K_C	1	1	1	1	1	1	1	1	1	1	1	1	1	1	1	1	1	1	1	1	1	1	1	1	1	1	1	1	1	1	1	1	1	1	1.0000
HRO_D1P5CS_1_A	0	0	1	1	1	1	0	0	0	0	1	0	0	0	0	0	1	0	0	1	0	0	0	0	0	0	1	0	0	1	1	0	0	1	1.0000
HRO_D1P5CS_1_B	0	0	0	1	1	0	1	1	1	1	0	1	1	1	0	0	1	1	1	0	0	0	0	1	0	0	0	0	0	1	0	0	0	0	0.0799
HRO_D1P5CS_1_C	0	0	0	0	0	0	0	0	0	0	0	0	0	0	0	0	0	0	1	0	0	0	0	0	0	0	0	0	0	0	0	0	0	0	1.0000
HRO_D1P5CS_1_D	0	0	0	0	0	0	0	1	1	0	0	0	0	0	0	0	0	0	0	0	0	1	1	0	0	0	0	0	0	0	0	1	1	0	0.6562
HRO_D1P5CS_1_E	1	1	1	1	1	1	1	1	1	1	1	1	1	1	1	1	1	1	1	1	1	1	1	1	1	1	1	1	1	1	1	1	1	1	1.0000
HRO_D1P5CS_2_A	1	1	0	0	0	0	0	0	0	0	0	0	0	0	0	1	0	0	1	0	0	0	0	0	0	1	0	0	0	0	0	0	1	0	1.0000
HRO_D1P5CS_2_B	1	1	1	1	1	1	1	1	1	1	1	1	1	1	1	1	1	1	1	1	1	1	1	1	1	1	1	1	1	1	1	1	1	1	1.0000
HRO_EBF1_1a_A	0	0	0	0	0	0	0	0	0	0	0	1	0	0	0	0	0	0	0	1	1	1	0	0	0	0	0	0	1	0	0	0	0	0	0.3353
HRO_EBF1_1a_B	1	0	1	0	1	1	1	1	1	1	0	1	1	1	0	1	1	1	0	1	1	1	1	1	0	1	1	1	0	1	1	1	1	1	1.0000
HRO_EBF1_1a_C	0	1	1	1	0	0	0	1	1	0	1	0	0	0	1	0	1	1	1	0	0	1	1	0	1	1	1	0	0	0	0	0	0	0	1.0000
HRO_EBF1_2_A	1	1	1	1	1	1	1	0	1	1	1	1	1	1	1	1	0	1	1	1	1	1	0	1	1	0	1	0	1	1	1	1	1	1	1.0000
HRO_EBF1_2_B	0	1	1	1	1	1	1	1	1	1	1	1	1	1	1	0	1	1	0	0	0	1	1	1	0	1	1	1	1	0	1	1	1	0	0.2245
HRO_EBF1_2_C	0	1	0	0	0	0	0	1	0	0	0	0	0	1	0	0	1	0	0	0	0	0	1	1	0	1	0	0	0	1	0	0	1	0	1.0000
HRO_EIL2_A	0	0	0	1	1	1	0	1	0	0	1	0	0	0	0	0	0	0	1	1	1	0	0	0	0	1	0	0	1	1	1	0	0	0	0.7207
HRO_EIL2_B	0	1	0	0	0	0	0	0	0	0	0	0	0	0	0	0	0	0	1	0	1	1	0	0	0	0	0	0	1	1	0	0	0	1	0.0854
HRO_EIL2_C	1	0	0	0	1	0	0	0	1	1	0	1	1	1	0	0	1	0	0	0	0	0	1	0	0	0	1	0	0	0	0	1	0	0	0.1411
HRO_EIL2_D	0	0	0	0	0	0	0	0	0	0	0	0	0	0	0	0	1	0	0	0	0	0	0	0	0	0	0	0	0	0	0	0	0	0	1.0000
HRO_EIL2_E	1	1	1	1	1	1	1	1	1	1	1	1	1	1	1	1	1	1	1	1	1	1	1	1	1	1	1	1	1	1	1	1	1	1	1.0000
HRO_EIN2_A	1	0	0	0	0	0	0	0	0	0	0	0	0	0	0	0	0	0	0	0	0	0	0	0	1	0	0	0	0	0	0	1	0	0	1.0000
HRO_EIN2_B	1	0	1	1	0	1	1	1	1	1	1	0	1	1	1	0	1	0	1	1	1	1	1	1	1	1	1	1	1	0	1	1	1	1	0.6562
HRO_EIN2_C	0	1	0	1	1	0	1	0	0	1	0	1	1	0	0	1	0	0	1	0	1	0	0	0	0	1	1	0	1	0	1	0	0	0	0.7283
HRO_EIN2_D	0	0	0	0	0	0	0	0	0	0	0	1	0	0	0	0	0	0	0	0	0	0	0	1	0	0	0	0	0	0	0	0	0	0	1.0000
HRO_EIN2_E	0	0	0	0	0	0	0	1	1	0	1	0	0	0	1	0	0	0	0	0	0	1	0	0	1	0	0	0	0	0	0	0	0	0	0.6562
HRO_EIN2_F	1	1	0	0	1	0	1	1	0	0	1	1	0	1	0	0	1	1	0	0	0	0	1	1	0	1	1	0	1	1	1	1	1	0	1.0000
HRO_EIN2_G	0	0	0	0	0	0	1	0	0	1	1	0	0	0	0	1	1	1	0	0	0	1	0	0	0	0	1	1	0	1	0	0	1	1	0.7207
HRO_EIN2_H	1	1	1	1	1	1	0	1	0	0	0	0	0	1	0	1	1	1	0	1	1	0	1	0	1	0	0	1	0	0	0	1	0	1	0.7319
HRO_EIX_1E_A	0	0	0	0	0	0	0	0	0	0	0	0	0	0	0	0	0	0	0	0	0	0	0	0	0	1	0	1	0	0	0	0	0	0	0.4848
HRO_EIX_1E_B	1	1	1	0	0	1	0	1	1	1	1	1	1	1	1	1	1	1	1	1	1	1	1	0	1	0	1	0	1	1	1	1	1	1	1.0000
HRO_EIX_1E_C	1	1	1	1	1	1	0	1	1	0	1	1	0	1	0	1	0	0	1	0	1	1	1	1	0	1	0	1	1	1	0	1	1	1	1.0000
HRO_EIX_1E_D	0	0	0	0	0	0	0	0	0	0	0	0	0	0	0	0	1	0	0	0	0	0	0	0	0	0	0	0	0	0	0	0	0	0	1.0000
HRO_EREBP1_1_A	0	0	0	0	0	0	0	0	0	0	0	0	0	0	0	0	0	0	0	0	0	0	0	0	0	0	1	0	0	0	0	0	0	0	1.0000
HRO_EREBP1_1_B	1	0	0	0	0	0	0	0	0	0	0	0	0	0	0	0	0	0	0	0	0	0	0	0	0	0	0	0	0	0	0	0	0	0	1.0000
HRO_EREBP1_1_C	0	0	0	0	0	1	0	1	0	0	0	0	0	0	0	0	0	0	0	0	0	0	1	0	1	0	0	0	0	0	0	0	0	0	1.0000
HRO_EREBP1_1_D	1	0	1	0	0	0	1	0	1	1	0	1	1	1	1	0	0	0	0	1	0	1	1	0	1	1	1	1	1	0	1	1	0	1	0.7283
HRO_EREBP1_1_E	1	1	1	1	1	1	1	1	1	1	1	1	1	1	1	1	1	1	1	1	1	1	1	1	1	1	1	1	1	1	1	1	1	1	1.0000
HRO_EREBP1_1_F	0	1	0	0	0	0	0	0	0	0	0	1	0	0	0	0	0	0	0	0	0	1	0	0	0	0	0	0	1	0	0	1	0	0	1.0000
HRO_EREBP1_1_G	0	0	0	0	0	0	0	0	0	0	0	0	0	1	0	0	0	0	0	0	0	0	0	0	0	0	0	0	0	0	0	0	0	0	1.0000
HRO_EREBP1_1_H	0	0	0	0	0	0	0	0	1	0	0	0	0	0	0	0	0	0	0	0	0	0	0	0	0	0	0	0	0	1	0	0	0	0	1.0000
HRO_EREBP_1a_A	1	1	1	1	0	0	1	0	1	0	1	1	1	1	0	1	1	0	1	0	1	0	0	1	0	1	1	1	1	0	1	1	0	1	0.7207
HRO_EREBP_1a_B	1	1	1	1	1	1	1	1	1	1	1	1	0	1	1	1	1	1	1	1	1	1	1	1	1	1	1	1	1	1	1	1	1	1	1.0000

HRO_EREBP_1a_C	0	0	0	0	0	0	0	0	0	0	0	0	1	0	0	0	0	0	0	0	0	0	0	0	0	0	0	0	0	1	0	0	0	0	1.0000
HRO_EREBPC2_1_A	1	1	1	1	0	0	1	0	1	1	1	1	1	1	1	1	1	1	0	1	1	1	1	1	1	1	0	1	1	1	1	0	0	0	0.6880
HRO_EREBPC2_1_B	0	1	1	1	1	1	1	1	1	1	0	0	1	1	0	0	0	0	0	0	1	1	0	1	1	1	1	1	1	1	0	1	1	1	1.0000
HRO_EREBPC2_1_C	0	0	0	0	0	0	0	0	0	0	0	0	0	0	0	0	0	0	0	0	0	0	0	0	0	0	0	0	0	0	0	0	1	1	0.4848
HRO_EREBPC2_1_D	0	0	0	0	0	0	0	0	0	0	0	0	0	0	0	1	0	0	1	0	0	0	1	0	0	0	0	0	1	0	0	1	1	1	0.0854
HRO_EREBPC2_1_E	1	0	0	1	1	1	1	1	0	1	1	1	1	1	0	1	1	0	1	1	1	1	1	1	1	1	1	1	0	0	1	0	0	1	1.0000
HRO_ERF114_A	1	1	1	1	1	0	1	1	1	1	1	1	0	1	1	1	1	1	1	1	1	0	1	1	1	1	1	1	1	1	1	1	1	1	1.0000
HRO_ERF114_B	1	0	0	0	0	1	0	0	0	0	1	0	1	0	0	0	1	1	0	1	1	1	0	1	1	0	0	0	1	0	1	0	0	0	0.4813
HRO_ERF141_A	0	1	1	1	1	1	0	1	1	1	1	1	1	1	1	1	1	1	1	0	1	1	1	1	1	1	1	1	1	1	0	1	1	1	1.0000
HRO_ERF141_B	1	0	1	1	1	0	1	1	1	0	1	1	0	0	0	1	1	0	1	1	1	0	1	0	1	1	1	0	0	0	0	0	0	1	0.4905
HRO_ERF141_C	0	0	0	0	0	0	0	0	0	0	0	0	0	0	0	1	0	0	0	0	0	0	0	0	0	0	0	0	0	0	0	0	0	1	1.0000
HRO_ERF141_D	0	0	1	0	0	0	1	0	0	1	0	0	1	1	0	0	0	1	1	0	0	0	0	0	0	1	0	0	0	0	1	1	1	1	0.7207
HRO_ERF141_E	1	1	0	1	1	1	1	1	1	1	1	1	1	0	1	1	0	0	1	1	1	1	1	0	1	0	1	1	1	0	1	1	1	1	1.0000
HRO_ERF4_A	1	1	1	1	1	1	1	1	1	1	1	1	1	1	1	1	1	1	1	1	1	1	1	1	1	1	1	1	1	1	1	1	1	1	1.0000
HRO_ERF4_B	0	0	0	0	0	0	0	0	0	0	0	1	1	0	0	0	0	0	1	0	0	0	0	0	0	0	0	0	0	0	0	0	0	0	1.0000
HRO_ERS1_1_A	1	1	1	1	1	0	1	1	1	1	1	0	1	1	1	0	0	1	1	1	1	1	1	1	1	1	0	1	1	0	1	1	1	1	0.6562
HRO_ERS1_1_B	0	0	0	0	0	0	0	0	0	0	0	0	0	0	0	0	0	0	0	0	0	0	0	0	0	0	0	0	0	0	1	0	0	0	1.0000
HRO_ERS1_1_C	0	0	0	0	1	1	1	1	1	0	0	1	1	1	0	1	0	1	1	1	0	0	1	1	1	0	1	1	0	1	1	0	1	1	0.4813
HRO_ERS1_1_D	1	1	0	1	0	0	0	1	1	0	1	1	0	0	1	0	1	0	1	1	1	0	1	1	1	0	0	0	0	0	0	0	1	0	0.7319
HRO_ERS1_1_E	0	1	1	0	0	1	1	1	1	1	1	1	1	1	1	1	1	1	1	1	1	0	0	1	1	1	1	1	1	1	1	0	0	1	1.0000
HRO_ERS1_1_F	0	0	0	0	0	0	0	0	0	0	0	0	0	0	0	0	0	0	1	0	1	0	0	0	0	0	1	0	1	0	0	1	0	0	0.0445
HRO_ERS1_1_G	0	0	0	0	0	0	0	0	0	0	0	0	0	0	0	1	0	0	0	0	0	0	0	0	0	0	0	0	0	0	0	1	0	0	1.0000
HRO_ETOP1_3_A	1	1	0	1	1	0	1	1	1	1	1	1	1	0	1	1	1	1	1	1	1	1	1	0	1	1	1	1	1	1	0	1	1	1	1.0000
HRO_ETOP1_3_B	0	1	1	1	0	1	1	1	1	0	0	0	0	1	1	1	1	1	0	0	0	1	1	1	1	1	1	1	0	0	1	0	1	1	1.0000
HRO_ETOP1_3_C	1	1	1	1	1	1	1	1	0	1	1	1	1	1	1	0	1	1	1	1	1	1	1	1	0	0	1	1	1	1	1	1	1	1	1.0000
HRO_ETR1_1A_A	0	1	1	1	1	1	0	1	1	1	1	1	1	1	0	1	0	0	0	0	1	1	1	1	0	1	1	1	0	0	1	0	0	0	0.1571
HRO_ETR1_1A_B	0	0	0	0	0	0	0	0	0	0	0	0	1	0	0	1	1	0	0	0	0	0	0	0	1	0	1	0	0	1	0	0	0	1	1.0000
HRO_ETR1_1A_C	1	1	1	1	1	1	1	1	1	1	1	1	1	1	1	1	1	1	1	1	1	1	1	1	1	1	0	1	1	1	1	1	1	1	1.0000
HRO_ETR1_1A_D	1	0	0	0	1	1	1	0	0	1	0	0	0	1	1	0	0	1	0	1	0	0	0	0	1	0	0	0	0	0	0	0	0	0	0.2587
HRO_ETRHOM_A	0	0	0	0	0	1	0	1	1	0	0	0	0	0	0	0	0	0	0	0	0	0	1	0	0	0	0	0	0	0	0	1	0	0	1.0000
HRO_ETRHOM_B	1	1	1	1	1	1	1	1	1	1	1	1	1	1	1	1	1	1	1	1	1	1	1	1	1	1	1	1	1	1	1	1	1	1	1.0000
HRO_ETRHOM_C	0	1	0	1	1	0	0	1	1	1	1	1	1	0	1	1	1	1	1	0	1	0	1	0	1	1	1	0	1	0	1	0	1	1	1.0000
HRO_ETRTF_5a_A	0	0	1	1	1	1	1	1	1	1	1	1	1	1	1	1	1	0	1	0	1	1	1	1	1	1	1	1	1	1	1	1	0	1	1.0000
HRO_ETRTF_5a_B	0	1	1	0	0	0	0	1	0	0	0	1	0	0	1	0	1	0	1	1	0	1	1	1	1	1	0	1	0	0	1	1	1	0	0.1694
HRO_ETRTF_5a_C	1	1	1	1	1	1	1	1	1	1	1	1	1	1	1	1	1	1	1	1	1	1	1	1	1	1	1	1	1	1	1	1	1	1	1.0000
HRO_ETRTF_5a_D	0	1	1	0	0	0	0	1	0	0	0	0	0	0	1	0	0	0	1	1	0	1	1	1	1	1	0	1	0	0	1	1	1	0	0.0366
HRO_ETRTF13_A	1	1	1	1	1	1	1	1	1	1	1	1	1	1	1	1	1	1	1	1	1	1	1	1	1	1	1	1	1	1	1	1	1	1	1.0000
HRO_ETRTF13_B	0	0	0	0	0	0	0	0	0	0	0	0	0	0	0	0	0	0	0	0	0	0	0	1	0	1	0	1	0	0	0	0	0	0	0.2273
HRO_ETRTF3_2_A	1	1	0	1	1	0	0	1	0	0	1	1	1	0	1	1	1	0	1	0	0	0	1	0	1	1	0	0	0	0	1	0	1	1	0.3028
HRO_ETRTF3_2_B	1	0	0	1	1	0	1	1	0	0	0	0	0	1	0	0	0	1	1	0	1	0	1	0	1	1	0	1	0	1	1	0	0	0	0.4905
HRO_ETRTF3_2_C	0	0	1	1	1	1	1	1	1	1	1	1	0	1	1	1	1	1	1	1	1	1	1	1	0	0	1	1	1	1	0	1	1	1	1.0000
HRO_ETRTF3_2_D	0	0	0	0	0	0	1	0	1	0	0	0	0	0	0	0	0	0	0	0	0	1	0	0	0	0	1	1	0	0	0	0	0	0	1.0000
HRO_EXT_1D_A	1	1	1	1	1	1	1	1	1	1	1	1	1	0	1	1	1	1	1	1	1	1	1	1	1	1	1	1	1	1	1	1	1	1	1.0000

HRO_EXT_1D_B	0	0	0	0	0	0	0	0	0	0	0	0	0	0	0	0	0	0	0	0	0	0	0	0	0	0	0	0	0	0	1	0	0	0	1.0000
HRO_EXT_1D_C	1	1	1	1	1	1	0	1	1	1	1	1	1	0	1	0	0	1	1	1	1	0	1	1	1	1	1	0	0	1	0	0	1	0	0.7080
HRO_EXT_1D_D	1	1	1	1	1	1	1	1	1	1	1	1	1	0	1	0	0	1	1	1	1	1	1	1	1	1	1	1	0	1	1	1	1	0	1.0000
HRO_EXT_1D_E	0	0	0	0	0	0	0	0	0	0	0	0	0	0	0	0	0	0	0	0	0	0	0	0	0	0	0	0	0	0	1	0	0	0	1.0000
HRO_FLS2_1A_A	1	1	1	1	1	1	1	1	1	1	1	1	1	1	1	1	1	1	1	1	1	1	1	1	1	1	1	1	1	1	1	1	1	1	1.0000
HRO_FLS2_1A_B	0	0	0	0	0	0	1	1	0	0	1	1	1	1	0	1	0	0	1	0	1	0	1	0	0	1	1	0	1	0	1	0	1	0	1.0000
HRO_FLS2_1A_C	0	0	1	0	0	0	0	0	0	0	0	0	0	0	1	0	0	0	0	0	0	0	0	0	0	0	0	1	0	0	0	0	0	0	1.0000
HRO_G3O_A	0	0	1	1	1	1	0	0	0	0	0	0	0	1	1	0	1	1	0	1	1	1	0	1	0	1	0	0	1	1	0	1	1	1	0.3028
HRO_G3O_B	1	1	1	1	1	1	1	1	1	1	1	1	1	1	1	1	1	1	1	1	1	1	1	1	1	1	1	1	1	1	1	1	1	1	1.0000
HRO_G3O_C	0	0	0	0	0	0	0	1	0	1	1	0	0	0	0	0	0	0	0	0	0	0	1	0	1	1	0	0	0	0	0	0	0	0	1.0000
HRO_GLUCT_7C_A	0	0	0	0	1	0	0	0	0	0	0	0	0	0	0	0	0	0	0	0	0	0	0	0	0	0	0	0	0	0	0	0	0	0	1.0000
HRO_GLUCT_7C_B	0	0	0	0	0	0	0	0	0	0	0	0	0	0	0	0	0	0	0	0	0	0	0	0	1	0	1	0	0	0	0	0	0	0	0.4848
HRO_GLUCT_7C_C	1	1	1	1	1	1	1	1	1	1	1	1	1	1	1	1	1	1	1	0	0	1	1	1	1	1	1	1	1	1	1	1	1	1	0.4848
HRO_GLUCT_7C_D	1	0	1	0	0	1	1	1	0	1	1	1	1	0	1	1	1	1	1	1	1	1	1	1	0	0	1	0	1	1	1	1	1	1	0.6880
HRO_GLUCT_7C_E	0	0	1	0	1	1	0	1	1	1	1	0	1	0	1	0	1	1	0	0	0	0	1	1	0	0	0	0	1	0	0	0	0	0	0.0799
HRO_GLUTST_1A_A	1	1	1	1	1	1	1	1	1	0	1	1	1	0	1	1	1	1	1	1	1	1	1	1	0	1	1	1	1	1	1	1	1	1	1.0000
HRO_GLUTST_1C_A	1	1	1	1	1	1	1	1	1	0	1	1	1	0	1	1	1	1	1	1	1	1	1	1	0	1	1	1	1	1	1	1	1	1	1.0000
HRO_JA2_1_A	0	0	0	0	0	1	0	0	0	0	0	0	0	0	0	0	0	1	0	0	0	0	0	0	0	0	1	0	0	0	0	0	0	0	1.0000
HRO_JA2_1_B	0	1	0	0	1	1	0	0	0	0	0	0	0	1	0	0	0	1	0	1	0	0	0	0	1	0	0	0	0	0	0	0	0	0	1.0000
HRO_KIN_1_A	1	1	1	1	1	1	0	1	1	1	1	1	1	1	1	1	1	1	1	1	1	1	1	1	1	1	1	0	1	0	0	1	1	1	0.6012
HRO_KIN_1_B	1	1	1	1	1	1	1	1	1	1	1	1	1	1	1	0	1	1	1	1	1	1	1	1	1	1	1	1	1	1	1	1	1	1	1.0000
HRO_KIN_1_C	1	1	1	1	1	1	1	1	1	1	1	1	1	1	1	1	1	1	1	1	1	1	1	1	1	1	1	1	1	1	1	1	1	1	1.0000
HRO_LEA_A	0	0	0	0	0	1	0	0	0	0	0	0	0	0	0	0	0	1	0	0	0	0	0	0	0	1	0	0	0	1	0	0	0	0	0.6012
HRO_LEA_B	1	0	1	1	1	0	0	0	0	1	1	1	0	1	1	0	1	0	1	0	1	0	1	0	0	0	0	0	0	0	0	1	0	1	0.1663
HRO_LEA_C	1	1	1	1	1	0	1	0	0	0	1	0	0	1	0	0	1	1	0	1	1	0	0	1	0	0	1	1	1	0	1	1	0	0	1.0000
HRO_LEA_D	0	0	0	0	0	0	0	0	0	1	0	0	1	0	0	0	0	0	1	0	0	1	0	0	0	0	0	0	0	0	0	0	0	0	1.0000
HRO_LEA_E	1	1	1	1	1	1	1	1	1	1	0	1	1	1	1	1	1	1	1	1	1	1	1	1	1	1	1	1	1	1	1	1	1	1	1.0000
HRO_LEA_F	1	1	1	0	0	0	0	1	0	0	0	0	0	0	0	0	1	0	0	1	0	1	1	0	1	0	0	0	0	0	0	0	0	1	1.0000
HRO_LIPOX_1B_A	0	0	1	1	0	1	1	1	0	0	1	1	1	0	0	1	0	1	1	0	1	1	0	0	1	0	1	0	1	1	1	1	1	0	0.7283
HRO_LIPOX_1B_B	1	1	1	1	1	1	1	1	1	1	1	1	1	1	1	1	1	1	1	1	1	1	1	1	1	1	1	1	1	1	1	1	1	1	1.0000
HRO_LRRSTPK_1C_A	0	0	0	0	0	0	0	0	0	0	0	1	0	0	0	0	0	0	0	0	0	0	0	0	0	0	0	0	0	0	0	1	0	0	1.0000
HRO_LRRSTPK_1C_B	1	1	0	1	1	1	1	1	1	1	1	1	1	1	1	1	1	1	1	1	1	1	1	1	1	1	1	1	1	1	1	1	1	1	1.0000
HRO_LRRSTPK_1C_C	0	0	0	0	0	0	0	0	0	0	0	0	0	0	0	0	0	0	0	0	0	0	1	0	0	0	0	0	0	0	0	0	0	0	1.0000
HRO_LRRSTPK_1C_D	0	0	0	0	0	0	0	0	0	0	0	1	0	0	0	0	0	0	0	0	0	0	0	0	0	0	0	0	0	1	0	1	0	0	1.0000
HRO_LRRSTPK_1C_E	1	1	1	1	1	1	1	1	1	1	1	1	1	1	1	1	1	1	1	1	1	1	1	1	1	1	1	1	1	1	1	1	1	1	1.0000
HRO_LRRSTPK_1C_F	0	1	1	1	1	1	0	1	1	1	1	1	1	0	1	1	1	1	1	0	0	0	1	1	1	1	1	0	1	1	0	1	1	0	0.4384
HRO_LRRSTPK_1C_G	0	0	0	0	0	0	0	0	0	0	0	1	0	0	0	0	0	0	0	0	0	0	0	0	0	0	0	0	0	0	0	1	0	0	1.0000
HRO_MOCOS_1A_A	1	1	0	1	1	1	1	1	1	1	1	1	1	1	1	1	1	1	1	1	1	1	1	1	1	1	1	1	1	1	1	0	1	1	1.0000
HRO_MOCOS_1A_B	0	0	0	0	0	0	0	0	0	0	0	0	0	0	0	0	1	0	0	0	0	0	1	0	0	0	0	0	0	0	0	0	0	0	1.0000
HRO_MOCOS_1A_C	1	0	1	0	0	0	0	1	1	0	0	1	0	0	0	0	0	0	0	0	0	0	1	0	1	0	0	0	0	1	1	0	0	0	1.0000
HRO_MRPATF_3D_A	1	1	1	0	1	1	1	1	1	0	1	1	1	1	0	1	0	1	1	1	1	1	1	0	0	1	1	1	1	1	0	1	1	0	1.0000
HRO_MTRNAS_1D_A	0	0	0	0	0	0	0	0	0	0	0	0	0	0	1	0	0	0	1	0	0	0	0	0	0	0	0	0	0	0	0	0	0	0	1.0000
HRO_MTRNAS_1D_B	0	0	1	0	0	1	1	0	1	1	1	1	1	0	1	1	1	1	0	1	1	1	0	1	1	1	0	1	1	1	0	0	0	1	1.0000

HRO_MTRNAS_1D_C	1	1	0	1	1	1	1	1	1	1	1	1	1	1	1	1	1	1	1	1	1	1	1	1	1	0	1	1	1	1	1	1	1	1	1.0000
HRO_MTRNAS_1D_D	0	0	1	1	1	0	0	0	0	0	0	0	0	1	0	0	1	0	0	0	0	0	0	0	0	1	1	1	0	0	0	1	1	0	1.0000
HRO_NCED_2_A	0	0	1	1	1	1	1	1	0	1	1	0	1	1	0	1	1	1	1	1	1	1	1	1	1	0	0	1	0	1	1	0	1	0	1.0000
HRO_NCED_2_B	1	1	1	1	1	1	1	1	1	1	1	1	1	1	1	1	1	1	1	1	1	1	1	1	1	1	1	1	1	1	1	1	1	1	1.0000
HRO_NCED_3_A	0	0	0	0	0	0	0	0	0	0	0	0	0	0	0	0	0	0	0	0	0	0	0	0	0	1	0	0	0	0	0	0	0	0	1.0000
HRO_NCED_3_B	1	0	0	0	0	0	0	0	1	0	0	0	0	0	1	0	0	0	0	0	0	0	0	0	0	0	0	0	0	0	0	0	0	0	0.2273
HRO_NCED_3_C	1	1	1	1	1	1	1	1	1	1	1	1	1	1	1	1	1	1	1	1	1	1	1	1	1	1	1	1	1	1	1	1	1	1	1.0000
HRO_NCED_3_D	0	0	0	0	0	1	0	0	0	0	0	0	0	0	0	0	0	0	0	0	0	0	0	0	0	0	0	0	0	0	0	0	0	0	1.0000
HRO_NDPK2_A	1	1	1	1	1	1	1	1	1	1	1	1	1	1	1	1	1	1	1	1	1	1	1	1	1	1	1	1	1	1	1	1	1	1	1.0000
HRO_NDPK2_B	0	1	1	1	1	0	0	1	0	1	1	0	0	1	1	1	1	1	0	0	0	0	1	0	1	1	0	1	1	1	0	1	1	1	1.0000
HRO_P5CR_2_1_A	1	1	1	1	1	1	1	1	0	1	1	0	1	1	1	0	1	0	1	0	1	1	1	1	1	0	1	1	1	1	1	1	1	0	1.0000
HRO_P5CR_2_1_B	0	0	1	1	0	1	1	1	1	1	1	1	1	0	1	1	0	1	1	0	1	1	1	1	1	1	1	1	1	1	0	1	0	1	0.6880
HRO_PARGH_1A_A	1	1	1	1	1	1	1	1	1	1	1	1	1	1	1	1	1	1	1	1	1	1	1	1	1	1	1	1	1	1	1	1	1	1	1.0000
HRO_PARGH_1A_B	0	0	0	0	1	0	0	1	0	0	0	1	0	0	0	0	0	0	1	1	1	1	1	0	0	1	1	1	1	0	0	0	0	1	0.0324
HRO_PARGH_1C_A	0	0	0	0	0	0	0	0	1	1	0	0	0	0	0	0	0	0	1	0	0	0	0	0	0	0	0	0	1	1	1	0	0	0	0.6562
HRO_PARGH_1C_B	1	1	1	1	0	1	1	0	1	1	1	1	1	1	1	1	1	1	0	1	1	1	0	1	1	1	1	0	0	1	1	1	1	1	0.6562
HRO_PARGH_1C_C	0	0	0	0	0	0	0	1	0	0	1	0	0	0	0	0	0	0	0	0	0	0	1	1	0	0	0	0	1	0	0	0	0	0	1.0000
HRO_POD1_2_A	1	1	1	0	1	1	1	1	1	1	1	0	1	1	1	0	1	1	1	1	1	1	1	1	0	1	0	1	1	1	1	1	1	0	1.3438
HRO_POD1_2_B	0	0	0	0	0	0	0	0	0	0	0	0	0	0	0	1	0	0	0	0	0	0	1	0	0	0	1	0	0	0	0	0	0	1	0.6012
HRO_POD1_2_C	0	0	0	0	0	0	0	0	0	0	0	0	0	0	0	0	0	0	0	1	0	0	0	0	0	1	0	0	0	1	0	0	0	0	0.2273
HRO_POD1_2_D	1	1	1	1	1	0	1	1	1	1	1	1	0	1	0	1	1	0	1	1	1	1	1	1	1	0	1	1	1	1	1	1	1	1	1.0000
HRO_POD1_2_E	0	0	1	0	0	1	0	0	0	1	0	0	0	0	1	0	0	1	0	0	1	1	0	0	1	0	0	1	1	1	0	0	1	1	0.1571
HRO_PP2C_A	1	1	1	1	1	1	1	1	1	1	1	1	1	1	1	1	1	1	1	1	1	1	1	1	1	1	1	1	1	1	1	1	1	1	1.0000
HRO_PP2C_B	0	1	0	0	0	0	0	1	0	1	1	0	0	0	0	0	0	1	1	1	0	1	1	0	1	0	1	1	0	1	1	1	0	0	0.0366
HRO_PP2C1_2_A	1	0	0	1	0	1	1	1	1	0	1	1	1	1	1	0	1	1	1	1	1	0	1	1	0	0	0	1	0	0	0	0	1	0	0.2960
HRO_PP2C1_2_B	1	1	1	1	1	0	0	1	1	1	1	1	1	1	1	1	1	1	1	1	1	1	1	1	1	1	1	1	1	1	1	1	1	1	0.4848
HRO_PP2C1_2_C	0	0	0	0	0	0	0	0	0	0	0	0	0	0	0	1	0	0	0	0	0	0	0	0	0	0	1	0	0	0	0	0	0	0	1.0000
HRO_RPK_10D_A	1	1	0	1	1	1	1	1	1	1	1	1	1	1	1	1	1	1	1	1	1	1	1	1	1	1	1	1	1	1	1	1	1	1	1.0000
HRO_RPK_10D_B	0	0	0	0	0	0	0	0	0	0	0	0	0	0	1	0	0	0	0	0	0	0	0	0	0	0	0	0	0	0	0	0	0	0	1.0000
HRO_RPK_10D_C	0	0	0	0	0	0	0	0	0	0	0	0	0	0	0	0	0	0	1	0	0	0	0	0	0	0	0	0	1	1	0	0	0	1	0.1026
HRO_RPK_10D_D	1	1	1	1	1	1	1	1	1	1	1	1	1	1	1	1	1	1	1	1	1	1	1	1	1	1	1	1	1	1	1	1	1	1	1.0000
HRO_SBT4B_1B_A	1	1	1	1	1	1	1	1	1	1	1	1	1	1	1	1	1	1	1	1	1	1	1	1	1	1	1	1	1	1	1	1	1	1	1.0000
HRO_SBT4B_1B_B	1	0	0	0	0	0	0	0	0	0	0	0	0	0	0	1	0	0	0	0	1	0	0	0	1	0	1	0	0	0	0	0	0	0	1.0000
HRO_SIPP_A	0	0	0	0	0	0	0	0	0	0	0	0	0	0	0	0	0	1	0	0	0	0	0	0	0	0	0	0	0	0	0	0	0	0	1.0000
HRO_SIPP_B	1	1	1	1	1	1	1	1	1	0	1	0	1	1	1	1	0	1	1	1	1	1	1	0	1	1	1	1	0	1	0	1	1	0	1.0000
HRO_SIPP_C	0	0	0	0	0	0	0	0	0	0	0	0	0	0	0	0	0	0	1	0	0	0	0	0	0	0	0	0	0	0	0	0	0	0	1.0000
HRO_SIPP_D	1	1	1	1	1	1	1	1	1	1	1	1	1	1	1	0	1	1	1	1	1	1	1	1	1	1	1	1	1	1	1	1	1	1	1.0000
HRO_SIPP_E	0	1	0	1	1	1	1	1	1	1	1	1	1	0	1	1	1	1	0	1	1	1	1	1	1	0	0	1	1	0	1	0	0	1	0.4384
HRO_SLSGPS6_5C_A	0	0	0	0	0	0	0	0	0	0	0	0	0	0	0	0	0	0	0	0	0	1	0	0	0	0	0	0	0	0	0	0	0	0	1.0000
HRO_SLSGPS6_5C_B	1	1	1	1	1	1	1	1	1	0	0	0	1	1	1	1	1	1	0	1	0	0	1	1	1	1	1	1	0	0	1	1	1	1	0.6880
HRO_SLSGPS6_5C_C	0	0	0	0	0	0	0	0	0	1	1	0	0	0	0	0	0	0	1	0	1	1	0	0	0	0	0	0	1	1	0	0	0	0	0.3983
HRO_STPK_A	1	1	1	1	1	1	1	0	1	1	1	0	1	1	1	1	1	1	1	1	0	0	1	1	1	1	1	1	1	1	1	1	1	1	1.0000
HRO_STPK_B	1	1	1	1	1	1	1	1	1	0	1	1	0	1	1	0	1	1	1	1	1	1	1	1	1	1	1	1	1	1	1	1	1	1	0.2273

HRO_STPK_C	1	0	0	0	0	0	1	0	0	1	0	0	0	0	1	1	0	0	0	0	1	0	0	0	1	1	0	1	0	0	1	1	1	1	0.4813
HRO_STPK_D	0	0	0	0	1	0	0	0	1	0	0	0	1	0	0	0	0	0	1	1	0	0	0	0	0	0	0	0	0	0	0	0	0	0	1.0000
HRO_STPK_E	0	0	0	0	0	1	0	0	0	0	1	1	0	0	0	0	1	0	0	0	1	1	0	0	0	0	0	0	1	1	0	0	0	1	1.0000
HRO_STPKPT_1A_A	0	1	0	1	0	1	0	0	0	1	0	0	1	1	0	1	1	1	0	0	0	0	0	1	0	0	0	0	0	1	0	0	0	1	0.2818
HRO_STPKPT_1A_B	1	1	1	1	1	1	1	1	1	1	1	1	1	1	1	1	1	1	1	1	1	1	1	1	1	1	1	1	1	1	1	1	1	1	1.0000
HRO_STPKPT_1A_C	0	0	0	1	1	0	0	0	1	0	0	0	0	0	0	0	0	0	0	0	0	0	0	0	1	0	0	0	0	0	1	0	0	0	1.0000
HRO_STPKPT_1B_A	0	0	1	0	0	0	0	0	0	0	0	0	0	0	1	0	0	0	0	1	0	0	0	0	0	0	0	1	0	0	0	0	0	0	1.0000
HRO_STPKPT_1B_B	1	1	1	1	1	1	1	1	1	1	1	1	1	1	1	1	1	1	1	1	1	1	1	1	1	1	1	1	1	1	1	1	1	1	1.0000
HRO_TNLDR_2A_A	1	1	1	1	1	1	1	1	1	1	1	1	1	1	1	0	1	1	1	1	1	1	1	1	1	1	1	1	1	1	1	1	1	1	1.0000
HRO_TNLDR_2A_B	0	0	0	0	1	0	0	0	0	0	0	0	0	0	0	0	0	0	0	0	0	0	0	0	0	0	0	0	0	0	0	0	0	0	1.0000
HRO_TNLDR_2A_C	1	0	1	0	0	0	1	0	1	1	1	1	1	1	1	1	1	1	1	1	0	0	1	1	1	0	1	0	1	1	0	1	1	1	1.2920
HRO_TPS1_A	1	1	1	1	0	1	1	1	1	1	1	1	0	0	1	1	1	1	1	1	1	1	1	0	1	1	1	1	1	1	1	1	1	1	0.6012
HRO_TPS1_B	1	1	1	1	1	0	1	0	1	1	1	1	1	1	0	0	0	1	1	0	0	1	0	1	0	0	1	1	0	1	1	1	1	0	0.7207
HRO_UDPGGT_1A_A	1	1	1	1	1	1	1	1	1	1	1	1	1	1	1	1	1	1	1	1	1	1	1	1	1	1	1	1	1	1	1	1	1	1	1.0000
HRO_UDPGGT_1A_B	0	0	1	0	1	0	0	0	0	0	0	1	0	1	0	0	0	0	0	1	1	0	1	1	1	0	0	0	0	0	0	1	0	0	0.7080
HRO_UGP_A	0	0	0	0	1	0	0	0	1	0	0	1	0	0	1	0	0	1	0	0	0	0	0	0	0	1	0	0	0	1	1	0	0	0	1.0000
HRO_UGP_B	0	0	1	1	0	0	0	1	0	0	0	0	0	1	1	0	1	0	1	1	1	0	1	0	1	0	1	1	1	0	0	0	0	1	0.4905
HRO_VPSP_1B_A	1	1	1	1	1	1	1	1	1	0	1	1	0	1	0	1	0	1	1	1	1	1	0	1	1	1	1	1	1	0	0	1	1	1	1.0000
HRO_VPSP_1B_B	0	0	1	0	1	1	1	0	1	0	1	0	0	1	0	0	0	1	0	1	1	1	0	1	0	1	1	0	0	0	0	1	0	0	1.0000
HRO_WRKYTF_1B_A	0	0	0	1	0	0	1	0	0	1	1	1	0	0	1	0	1	0	0	0	1	0	0	0	1	0	0	0	1	0	1	0	1	1	1.0000
HRO_WRKYTF_1B_B	1	1	1	1	1	1	1	0	1	1	0	1	0	1	1	0	1	1	0	1	1	0	1	0	1	0	1	1	0	1	0	1	0	1	0.4646
HRO_ZE1_A	1	1	1	1	1	1	1	1	1	1	1	0	1	1	1	0	1	1	1	1	1	1	1	1	1	1	0	1	1	1	1	1	1	1	1.0000
HRO_ZE1_B	0	0	0	0	0	0	0	0	0	0	0	0	0	0	0	0	0	0	0	1	0	0	0	0	0	0	0	0	0	1	0	0	0	0	0.4848
HRO_ZE1_C	0	0	1	0	1	1	1	0	1	0	1	1	0	1	1	0	1	0	1	1	0	1	0	0	1	0	0	1	1	0	0	0	1	1	0.7319
HRO_ZE1_D	0	0	0	0	0	0	0	0	0	0	0	0	0	0	0	1	0	0	0	0	1	0	1	0	0	0	1	0	0	0	0	0	0	0	0.6012

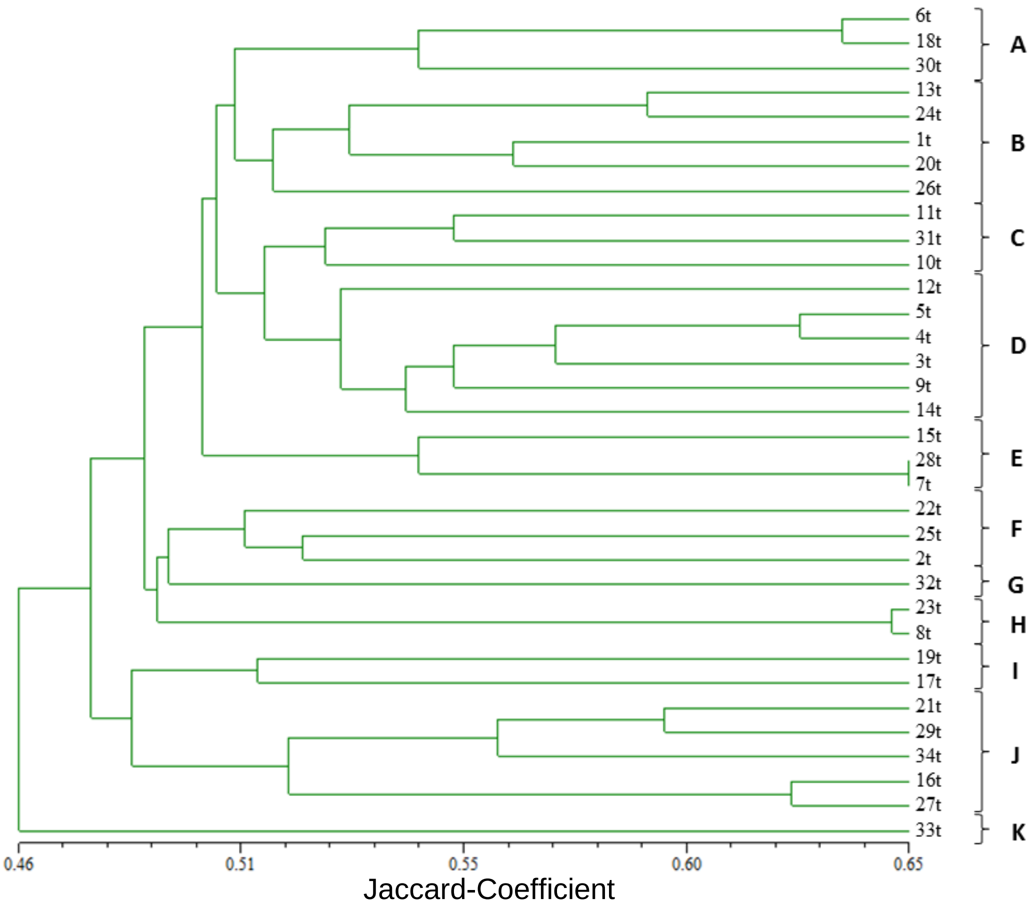

Figure S1. Population structure in the investigated association panel of 34 starch potato cultivars based on 54 linkage group specific SSR primer combinations and 19 AFLP primer combinations. The UPGMA dendrogram based on the Jaccard's coefficient is shown. The potato cultivars are shown with the drought tolerance rank from 1t = most tolerant to 34t = most sensitive.
